# Uncovering the Mechanism of the Xingnaojing Injection against Ischemic Stroke Using a Combined Network Pharmacology Approach and Gut Microbiota Analysis

**DOI:** 10.1155/2022/5886698

**Published:** 2022-05-20

**Authors:** Ganlu Liu, Jingfeng Lin, Lina Zhang, Qiang Gao, Zhenyi Wang, Ze Chang, Ying Gao, Dayong Ma, Zhenyun Han

**Affiliations:** ^1^Beijing University of Chinese Medicine, Beijing 100029, China; ^2^Hangzhou Seventh People's Hospital, Hangzhou 310000, China; ^3^Institute for Brain Disorders, Beijing University of Chinese Medicine, Beijing 100700, China; ^4^Department of Neurology, Dongzhimen Hospital, Beijing University of Chinese Medicine, Beijing 100700, China; ^5^Shenzhen Hospital of Beijing University of Chinese Medicine (Longgang), Shenzhen 518172, China

## Abstract

**Objective:**

To explore the brain protection mechanism of Xingnaojing injection (XNJ) against ischemic stroke (IS) by the network pharmacology approach and gut microbiota analysis.

**Methods:**

We used network pharmacology analysis to identify the active components of XNJ and its potential targets against IS and inflammatory bowel disease (IBD) and carried out network analysis, functional annotation, and pathway enrichment analysis. Then, transient middle cerebral artery occlusion (tMCAO) mice model was used to verify the molecular mechanism of XNJ.

**Results:**

36 active compounds were identified from XNJ, and the effect of XNJ against IS was related to the VEGF signaling pathway, NF-kappa B signaling pathway, and gap junction. The effect of XNJ against IBD was related to the T cell receptor signaling pathway, NF-kappa B signaling pathway, and gap junction. In vitro experiments showed that XNJ significantly improved the neurological function of tMCAO mice, reduced the size of cerebral infarction, decreased the permeability of blood-brain barrier (BBB), downregulated the expressions of TLR4, MyD88, and NF-kappa B in the ischemic site, and upregulated the expressions of occludin and ZO-1 in the colon. High-throughput 16S rDNA gene sequencing showed that XNJ upregulated the levels of *Akkermansia* and downregulated the levels of *Flavobacteriaceae*, *Deferribacteraceae*, and *Deferribacteres*. XNJ increased the concentrations of the short-chain fatty acids (SCFAs) PA (propionate), VA (valerate), IBA (isobutyrate), and IVA (isovalerate) in the feces of the sham germ-free experiment group (SGFEG) mice.

**Conclusion:**

IS causes dysbiosis of some specific bacteria in the gut microbiota. XNJ is an effective treatment for IS, and its mechanism was related to improving intestinal barrier function and regulating intestinal flora and SCFAs. Network pharmacology revealed that XNJ acts through multiple targets and multiple pathways.

## 1. Introduction 

Stroke is the second leading cause of death and disability in the world, and IS, which accounts for approximately 87% of all stroke cases, seriously endangers human health and is a public health problem of global concern [1, 2]. The burden of stroke is heavy in China. The prevalence rate of stroke is increasing year by year, and the mortality rate accounts for about one-third of the world [3, 4]. In recent years, IS has been found to be related to the imbalance of gut microbiota, and altering the gut microbiota has the therapeutic potential [5]. In addition, the gut-brain axis (GBA) has been defined to include a two-way communication network between the brain and the gastrointestinal tract, which connects the central cognitive and emotive centers of the brain with peripheral intestinal processes [6]. This interaction between the gut microbiota and brain is bidirectional, involving neural, endocrine, immune, and humoral links [7]. Previous studies have shown that stroke leads to gut microbiota dysbiosis which may also be a risk factor for stroke and may affect stroke outcomes [8–11].

There have been very limited treatment options for IS so far [12]. The blockages in IS are usually caused by a blood clot that becomes lodged in an artery in the brain. At present, tissue plasminogen activator (tPA) is the only therapeutic approved by the FDA for the treatment of IS. However, the treatment must be administered within 4.5 hours of the onset of stroke symptoms; otherwise, the risk of hemorrhagic transformation increases, which can cause additional damage to the brain [13]. Therefore, it is urgent to develop safe and effective therapies to treat IS. XNJ has been widely used in the treatment of IS in China, and it could improve neurological dysfunction with good clinical efficacy and fewer adverse reactions [14, 15].

XNJ is extracted from the Chinese ancient prescription “An-Gong-Niu-Huang Pill,” which consists of four Chinese herbs (Table 1) [16]. XNJ has been approved by China Food and Drug Administration (CFDA) with very strict quality control standards and is the only Chinese herbal medicine injection approved for stroke as an ambulance first aid choice in China [14, 16]. XNJ has the functions of enlightening the brain, calming pain, clearing heat and detoxification, tranquilizing the mind, and cooling the blood in Chinese medicine and is widely used for stroke in China [17–19]. Basic experimental studies also showed that XNJ could significantly reduce neuronal apoptosis, brain edema, and cerebral infarction area [20]. The following is the therapeutic mechanism. XNJ could regulate oxidative stress, and it could inhibit inflammatory response by promoting the expression of SIRT1, inhibiting the expression of NLRP3, and reducing the expression of TNF-*α*, IL-6, and IL-1*β* [20–22]. XNJ could prevent autophagy in experimental stroke by repressing the p53-DRAM pathway [23], protecting against cerebral ischemia-reperfusion injury via PI3K/Akt-mediated eNOS phosphorylation [16], and alleviating blood-brain barrier disruption by upregulating the expression of tight junction protein, occludin, and ZO-1 [22].

Notably, XNJ was also found to protect intestinal mucosal barrier function and reduce diamine oxidase (DAO) and fructose/mannitol (L/M). The protective effect of XNJ on intestinal mucosa may be to protect the intestinal immune system by inhibiting the adhesion of intestinal bacteria to the intestinal mucosa, maintain the morphological integrity of intestinal mucosal epithelium by reducing the apoptosis of intestinal mucosal cells, and enhance the proliferation of intestinal mucosa cells by providing energy [24]. These conclusions were also supported by experimental studies showing that XNJ prevented endotoxins from penetrating the intestinal mucosa into the circulatory system under infection and stress [25].

Multitarget is considered a characteristic of Chinese herbal medicine [26]. As a compound preparation of traditional Chinese medicine, XNJ not only protects the brain but has protective and repair effects on the intestinal tract. In this study, we proposed a hypothesis that XNJ played an indirect role in brain protection by regulating intestinal flora. Therefore, we conducted network pharmacological analysis and animal experiments to verify this hypothesis.

## 2. Material and Methods

### 2.1. Network Pharmacology Analysis

#### 2.1.1. Identification, Screening, and Analysis of Chemical Constituents in XNJ

The chemical constituents of XNJ were identified and screened by ultra-high-performance liquid chromatography-mass spectrometry (UHPLC-MS). The SCIEX 5600+ mass spectrometer was connected with Nexera UHPLC LC-30A (component name: Hybrid Quadrupole-TOF LC/MS/MS Mass Spectrometer; component ID: Triple TOF 5600+; manufacturer: AB Sciex Instruments. Shimadzu LC30). The determination was performed on a Waters HILIC BEH column (100 × 3 mm, 1.7 *µ*m). The column temperature was 35°C. The flow rate was 0.300 (ml/min). The mobile phase was (A) Quate = “acetonitrile” and (B) Quate = “0.1% CH3COOh-H2O.” Chromatographic conditions were shown in Table 2.

The positive and negative ionization modes of electrospray ionization (ESI) were used for mass spectrometry. ESI source conditions are as follows: ion source gas 1 (Gas 1): 50, ion source gas 2 (Gas 2): 50, curtain gas (CUR): 25, source temperature: 500°C (positive ions) and 450°C (negative ions), ion sapary voltage floating (ISVF) 5500 V (positive ions) and 4400 V (negative ions), TOF MS scan range: 100–1200 Da, product ion scan range: 50–1000 Da, TOF MS scan accumulation time 0.2 s, and product ion scan accumulation time 0.01 s. The secondary mass spectrometry was obtained by information-dependent-acquisition (IDA) with high sensitivity mode (declustering potential (DP): ±60 V; collision energy: 35 ± 15 eV).

#### 2.1.2. Prediction of Compounds and Disease-Related Targets

The chemical formula of the compound was downloaded from the PubChem database (https://pubchem.ncbi.nlm.nih.gov/) and imported into the SwissTargetPrediction database (http://swisstargetprediction.ch/). The target with parameter probability >0 was selected for further analysis.

Disease targets were collected from GeneCards (https://www.genecards.org/) and DisGeNET (https://www.disgenet.org/) databases using “Ischemic stroke” (IS) and “Inflammatory bowel disease” (IBD) as keywords. The target species is limited to “Homo sapiens”.

#### 2.1.3. Network Construction and Analysis

The targets of XNJ components were intersected with the disease targets of IS and IBS, respectively, and then, the components of XNJ and the drug-component-disease target interaction network were established and visualized by Cytoscape 3.7.2 software. In addition, protein-protein interaction (PPI) networks of putative therapeutic targets were established by the String website (https://cn.string-db.org/), with species selection as “*Homo sapiens*” and a confidence score set above 0.7. The data were imported into Cytoscape 3.7.2 software for visual processing to obtain the core targets of the PPI network.

The intersection targets of XNJ components and diseases were imported into the DAVID database (https://david.ncifcrf.gov/) for Gene Ontology (GO) and Kyoto Encyclopedia of Genes and Genomes (KEGG) Pathway Enrichment Analysis. The screening conditions were set as *P* < 0.01. The results were visualized using the bioinformatics website (http://www.bioinformatics.com.cn/).

### 2.2. Experimental Materials

Male C57BL/6 mice (7–8 weeks old), weighing 22–24 g, were provided by SPF Biotechnology Co., Ltd., and the certificate number is No. 1103242011003580. The main reagents and equipment are shown in Table 3.

### 2.3. Experimental Methods

#### 2.3.1. Animal Grouping and Intervention

One hundred and twenty male healthy C57 mice were randomly divided into 7 groups by the random number table method. The groups of mice and their interventions are outlined in Table 4. The mice were housed under standard conditions of light and dark cycles with free access to food and water (temperature 25°C, humidity 13%). All mice were fed sterile common feed in a single cage in the SPF animal center and weighed once a day. All the animal studies were carried out according to the approved protocols and guidelines of Dongzhimen Hospital Ethics Committee, Beijing University of Chinese Medicine.

We depleted the gut microbiome of mice by administration of antibiotics [27, 28]. Mice received ampicillin (1 g/L, Tenglong Medicine, Jiangxi), metronidazole (1 g/L, Shijiazhuang No. 4 Pharmaceutical), neomycin sulfate (1 g/L, Meidiya Biotech Company, Shanxi), and vancomycin (0.5 g/L, LUMMY, Chongqing) and with 5% sucrose in drinking water. All drinking water solutions were prepared and changed every two days for a total of two weeks and blinded for experimenters. Mice that received the antibiotic treatment were referred to as sham germ-free (SGF) mice. Mice in NG, SG, CG, and EG were given normal sterile water. On the last two days of the 14-day period and 2 days before modeling, we injected Xingnaojing (6 mg/kg, Jemincare Medicine, Jiangxi, Chinese approval number: Z32020562) into the SGFEG and EG mice abdominally.

#### 2.3.2. Animal Modeling

Transient middle cerebral artery occlusion (tMCAO) was performed with reference to previously reported procedures [29]. Briefly, after a skin incision along the midline of the mouse neck, the inner and outer muscles of the sternocleidomastoid muscle were separated to expose and isolate the left common, external, and internal carotid arteries. Subsequently, the superior thyroid and occipital arteries were separated. A 6–0 nylon monofilament coated with silicon rubber (Doccol) was inserted into the left external carotid artery and advanced until it obstructed the middle cerebral artery, and the common carotid artery was ligated for 35 min. After that, reperfusion was introduced by filament withdrawal, and the incision was sutured. Throughout the whole procedure, body temperature was maintained at 37 ± 0.5°C. After the mice were awake, the neurological function was scored according to Longa's 5-point system [30].

Zero points meant no neurological deficits. One point meant a mild loss of nerve function: limited extension of the right forelimb and flexion when the tail is lifted. Two points meant moderate neurological deficit: rotating to the paralyzed side (right side) when crawling. Three points meant severe neurological deficit: falling into the paralyzed side (right side) while crawling. Four points meant difficulty in crawling and performance of decreased consciousness. Mices with a score of 2 to 3 were included in the experiment.

### 2.4. Sample Collection and Index Detection

Three days after modeling, the mice were killed by quick decapitation and transferred to an ultraclean worktable for dissection. Then, 75% alcohol was used to clean the abdomens, and an incision was made in the abdominal cavity to completely separate the intestines. One to two mouse feces were collected from the end of the colon and stored in a sterilized EP tube. The feces were rapidly frozen and stored in a freezer at −80°C.

#### 2.4.1. Blood-Brain Barrier (BBB) Permeability Assay [31]

Mice were injected with 2% Evans blue (EB) solution into their tail veins (4 mg/kg). After 30 minutes, the mice were anesthetized with isoflurane and were transcardially perfused with 20–30 ml of heparin sodium normal saline (20 U/(0.9% NaCl) mL) to wash out the remnant dye until fluid from the right auricle became colorless. The left hemisphere was isolated and mashed in a centrifuge tube before being added to formamide (100 mg/mL) for incubation (24 h, 60°C) and being centrifuged at 1500 r for 5 min. The standard solution of 0, 10, 20, 40, 80, 160, 320, and 640 ng/ml was configured. The supernatant was taken and compared with the sample to be tested on the machine (equipment: UV spectrophotometer, detection model: D-8PCS, absorbance, 620 nm). Oringen 7.0 was used for statistical analysis of the data, and the content of EB in each group was calculated according to the standard curve of EB.

#### 2.4.2. Triphenyl Tetrazolium Chloride (TTC) Staining

After the mice were euthanized, the mouse brain was separated quickly to maintain the integrity of the brain and was frozen in a −20 degree refrigerator for about 20 minutes to be sliced. The coronal brain slices at 2-mm intervals were placed in preheated 1% 2, 3, 5-triphenyl tetrazolium chloride (TTC) and incubated for 20–30 min at 37°C. After that, the slices were placed evenly on the background plate containing the ruler and photographed. The cerebral infarct area was measured with Image pro plus 6.0, and the infarct volume was calculated using the following formula: *V* = (*S*1 + *S*2) × *d*/2 (*V*: total volume; *S*1: cranial area of slice; *S*2: caudal area of slice; d: the thickness of slice). The percentage of the infarct volume was calculated using the following equation: ipsilateral Infarct volume/contralateral hemispheric volume × 100%. Each brain was coronally sliced into 6 2-mm slices with a brain matrix on ice.

#### 2.4.3. Histopathological Examination

The tissue was fixed, dehydrated, and transparent and then embedded in paraffin and sectioned. Then, it was stained with hematoxylin and eosin (H&E) and observed under a microscope and photographed in 100× and 400× images (inverted microscope, Nikon CI-S; imaging system, Nikon DS-U3).

#### 2.4.4. Quantitative Real-Time Reverse Transcription PCR

Total RNA was extracted from tissue using a Total RNA Extraction Kit (R1200, Solarbio, Beijing, China). Reverse transcription and quantitative PCR were conducted using Universal RT-PCR Kit (M-MLV, free Taq polymerase, RP1105, Solarbio, Beijing, China) and RT-PCR QuantiTect SYBR Green kit (RP1110, Solarbio, Beijing, China). The relative expression level of the target gene was determined by the 2^−ΔΔ*Ct*^ method (Livak method), and GAPDH was utilized as an internal control. The RT-qPCR primers are listed in Table 5.

#### 2.4.5. Western Blot

Total proteins from the ischemic cortical area brain tissues and the colon tissues were collected in equal amounts of cell lysate, and the separated proteins in the supernatant were subsequently transferred. For immunoblotting, the following primary antibodies were used: occludin (1 : 1,000; Abcam, ab216327), MMP-9 (1 : 1,000; Abcam, ab38898), MyD88 (1 : 1,000; Gene Tex, GTX112987), NF-kB p65 (1 : 1,000; Abcam, ab16502), Z0-1(1 : 500; Abcam, ab96587), and TLR4 (1 : 500; Abcam, ab13556). After the incubation, the membrane was washed with TBST three times, each time for 10 minutes. The membrane was then incubated with goat anti-rabbit (1 : 5000, Biosynthesis, bs-0295G-HRP) and goat anti-mouse IgG antibodies (Biosynthesis, bs-0296G-HRP) at room temperature (25°C) for 1 hour and then washed 3 times with TBST. Finally, the enhancement solution of ECL reagent and stable peroxidase solution were mixed in a ratio of 1 : 1 and then dropped onto the PVDF membrane. The reaction lasted for several minutes until the fluorescence band was obvious. The grayscale value of the protein band was analyzed by using Image J. All experiments were performed three times.

#### 2.4.6. Enzyme-Linked Immunosorbent Assay (ELISA)

The plasma of the mouse was collected and measured using the Mouse Lipopolysaccharides (LPS) ELISA Kit (Jianglaibio, JL20691). All ELISA procedures were performed according to the manufacturer's instructions, and the absorbance was measured with a microplate reader (BioTek, Synergy H4) at 450 nm. Each determination was made in triplicate.

#### 2.4.7. Mouse Droppings Sequencing Experiment

Mouse fecal samples were removed from the −80°C freezer and then immediately sent to Tianhao Biotech Company (Shanghai) on dry ice for sequencing. The sequencing procedure was as follows: an excrement gene DNA extraction kit was used to extract mouse fecal DNA, and after the quality of the DNA was verified by 1% agarose gel electrophoresis, polymerase chain reaction (PCR) was performed. Three identical PCR experiments were performed for each sample. The v3-v4 region of the 16S rRNA gene was selected to be amplified to synthesize a fusion primer with dislocation bases. The number of amplification cycles of the samples was guaranteed to be consistent, and the majority of samples were amplified to show products at the appropriate concentration. The three PCR products of the same sample were mixed and detected by 2% agarose gel electrophoresis, and then, the PCR products were recovered using an AxyPrep DNA gel extraction kit. The PCR products were quantified by an enzyme marker and mixed in proportion. After a MiSeq PE library was constructed, Illumina MiSeq PE 300 platform was used for high-throughput sequencing.

### 2.5. Statistical Analysis

#### 2.5.1. Measurement Data

SPSS 19.0 was used for statistics. Measurement data were expressed as mean ± standard deviation (SD), and the ANOVA was used for comparison between groups. Data that did not conform to the normal distribution were represented by M (IQR), and the nonparametric test was used for comparison between groups. *p* < 0.05 was considered statistically significant.

### 2.6. Biological Information Analysis

TrimGalore software was used to remove the bases with a terminal mass of quality less than Q20 and to remove the adapter sequences that might be included. Then, short sequences with a length of less than 100 bp were removed. Using FLASH2 software to splice the paired sequences obtained by double-terminal sequencing to obtain the merged sequence, the low-quality sequences were further removed after merging. Then, mothur software was used to find and remove primers in the sequence, and Usearch software was used to remove sequences with which the total base error rate was greater than Q20 or the sequence length was less than 100 bp to obtain clean reads with high quality and reliability, which were then used for subsequent bioinformatics analysis. Qiime (v1.8.0) software was used for operational taxonomic unit (OTU) clustering; species annotations with greater than 97% similarity were assorted to an OTU. The representative OTU sequences were compared with the corresponding microbial database to determine the species classification of each sample, and OTUs were annotated at all levels.

#### 2.6.1. Community Composition Histogram and Diversity Analysis

Alpha diversity analysis, community composition analysis, and beta diversity analysis were carried out based on the clustering results. We used R software (version 3.6.2) to perform alpha diversity analysis, community composition analysis, and Beta diversity analysis. We used the R packages microbiome (version 1.8.0) and microbiomeSeq (version 0.1) to complete the analysis.

#### 2.6.2. SCFA's Preparation and Extraction

Fecal samples were weighed, 20 mg of each sample was placed in a 2-ml EP tube, and 1 mL of phosphoric acid (0.5% v/v) was added to each EP tube. The tubes were then vortexed for 10 min and ultrasonicated for 5 min. Then, 0.1 mL of supernatant was added to each 1.5 mL centrifugal tube, 0.5 mL of MTBE (containing the internal standard) was added, and the tubes were then vortexed for 3 minutes and ultrasonicated for 5 minutes. After that, the tubes were centrifuged for 10 minutes at 12,000 r/min and 4°C. After centrifugation, 0.2 mL of supernatant was absorbed into the sampling bottle for GC-MS/MS analysis.

## 3. Results

### 3.1. Identification of the Components in XNJ

**Figure 1:**
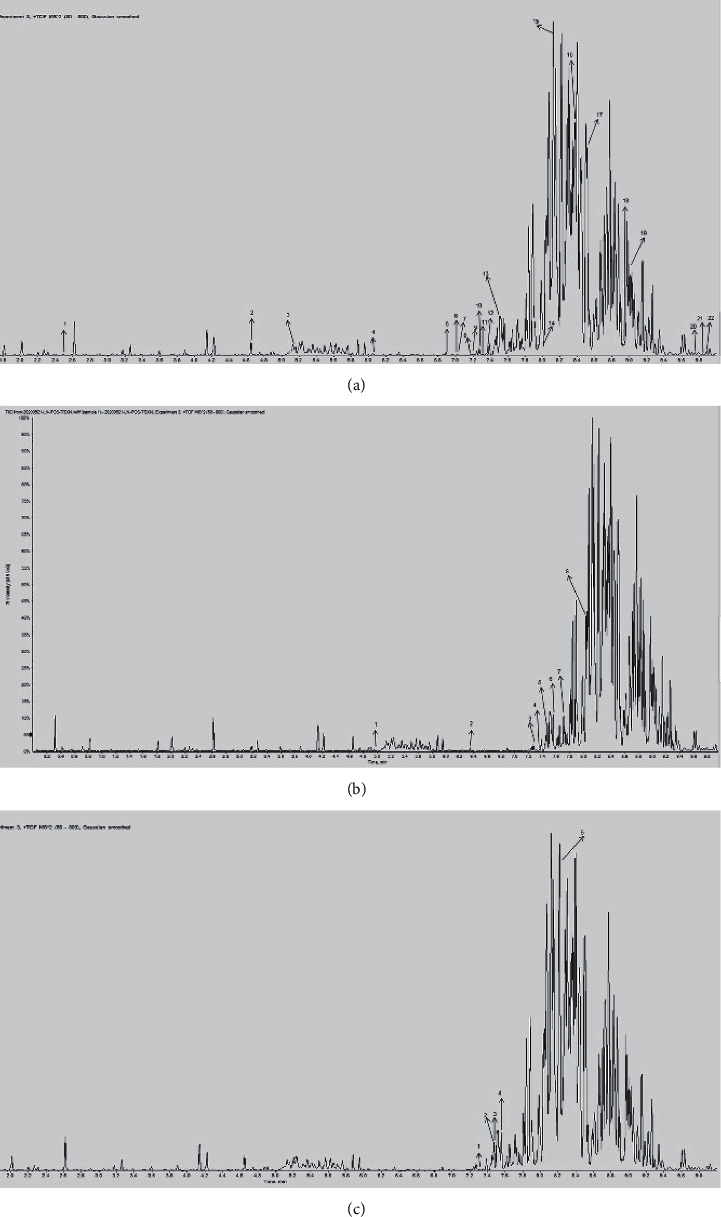
Total ion chromatogram of XNJ in positive ion mode. (a) *Radix Curcumae*; (b) *Moschus*; (c) *Borneolum Syntheticum*; (d) *Fructus gardenia*.

**Figure 2:**
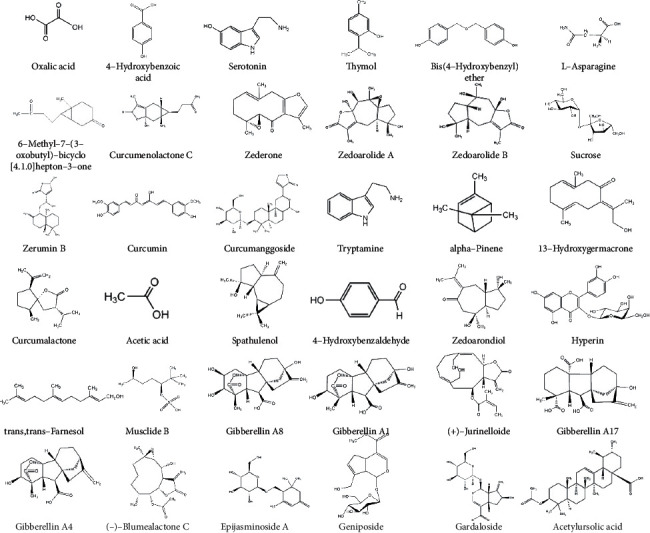
Chemical structures of the 36 components.

The UHPLC-MS spectra of SZJNF of XNJ are shown in Figure 1 and Supplementary Table S1. A total of 36 components were identified, including 23 ingredients of *Radix Curcumae*, 8 ingredients of *Moschus*, 1 ingredient of *Borneolum Syntheticum*, and 4 ingredients of *Fructus gardeniae*. The chemical structure of the compound was verified from Drug Bank database and PubChem database (Figure 2).

### 3.2. Construction of the Drug-Component-Target Network

**Figure 3:**
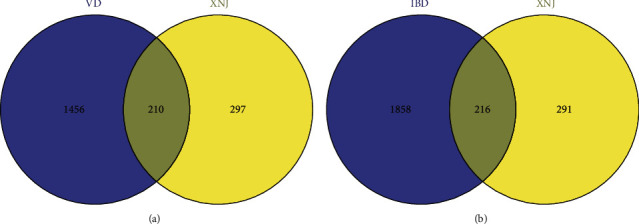
Venn diagram. (a) Venn diagram of related targets of ingredients of XNJ in the treatment of IS; (b) Venn diagram of related targets of ingredients of XNJ in the treatment of IBD.

A total of 507 compound-related targets were screened after the exclusion of duplicates (Supplementary Table S2). In addition, 1667 IS-related targets and 2074 IBD-related targets were identified from two databases (DisGeNET and GeneCard, Supplementary Table S3). From 507 predicted targets of the 36 components in XNJ and 1667 IS-related targets, 210 shared targets were identified as potential therapeutic targets of XNJ against IS (Figure 3(a), Supplementary Table S4). From 507 predicted targets of the 36 components in XNJ and 2047 IBD-related targets, 216 shared targets were identified as potential therapeutic targets of XNJ against IBD (Figure 3(b), Supplementary Table S4).

**Figure 4:**
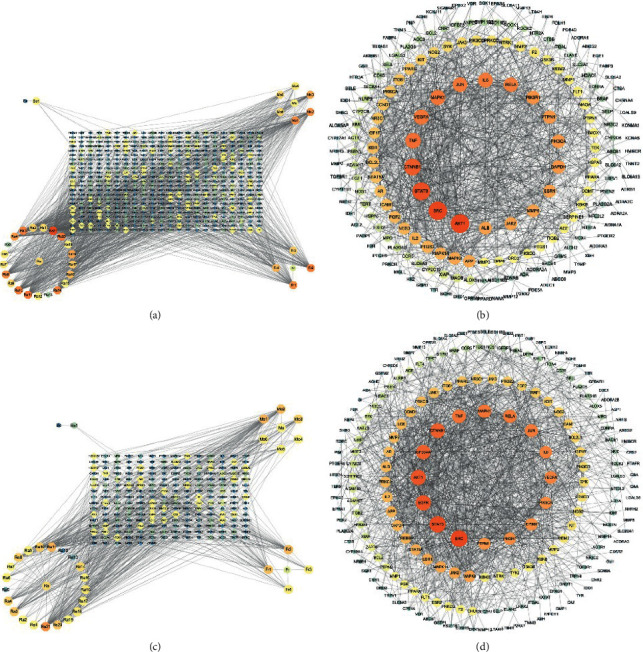
Drugs-active components-target network and PPI network. (a) Drugs-active components-target network diagram of XNJ in the treatment of IS; (b) PPI network of XNJ-IS; (c) drugs-active components-target network diagram of XNJ in the treatment of IBD; (d) PPI network of XNJ-IBD. Note: (Bo) Borneolum Syntheticum, (Mo) Moschus, (Ra) Radix Curcumae, (Fr) Fructus gardenia, (Bo1) (-)-Blumealactone (C), (Mo1) Gibberellin A1, (Mo2) Gibberellin A4, (Mo3) Gibberellin A8, (Mo4) Gibberellin A17, (Mo5) Hyperin, (Mo6) trans, trans-Farnesol, (Ra1) 4-Hydroxybenzaldehyde, (Ra2) 4-Hydroxybenzoic acid, (Ra3) 6-Methyl-7-(3-oxobutyl)-bicyclo[4.1.0]heptan-3-one, (Ra4) 13-Hydroxygermacrone, (Ra5) acetic acid, (Ra6) alpha-Pinene, (Ra7) Bis(4-Hydroxybenzyl) ether, (Ra8) Curcumalactone, (Ra9) Curcumanggoside, (Ra10) Curcumenolactone (C), (Ra11) Curcumin, (Ra12) L-Asparagine, (Ra13) Oxalic acid, (Ra14) Serotonin, (Ra15) Spathulenol, (Ra16) Sucrose, (Ra17) Thymol, (Ra18) Tryptamine, (Ra19) Zederone, (Ra20) Zedoarondiol, (Ra21) Zerumin (B), (Fr1) Acetylursolic acid, (Fr2) Epijasminoside (A), (Fr3) Gardaloside, and(Fr4) Geniposide.

The drug-compact-target network consisting of 36 components and 210 common targets associated with IS was constructed, with a total of 543 nodes and 1226 edges (Figure 4(a)). In particular, 6-Methyl-7-(3-oxobutyl)-bicyclo[4.1.0]heptan-3-one, Curcumenolactone C, Zedoarondiol, Zerumin B, Acetylursolic acid, Epijasminoside A, Gibberellin A1, and Gibberellin A4 were important active substances, and the top 10 potential targets were HSD11B1, PTGS2, PTPN1, Ra17, NR3C1, NR3C2, PRKCA, SLC6A4, ADRA2C, and PTGES. The PPI network consists of 197 nodes and 979 interactive edges (Figure 4(b)). The drug-compact-target network consisting of 36 components and 216 common targets associated with IBD was constructed, with a total of 252 nodes and 581 edges (Figure 4(c)). The top 10 potential targets were HSD11B1, CA2, PTGS2, CYP19A1, PGR, NR3C1, NR3C2, PRKCA, EGFR, and SLC6A4. The PPI network consists of 206 nodes and 1296 interactive edges (Figure 4(d)).

### 3.3. Functional and Pathway Enrichment Analysis

**Figure 5:**
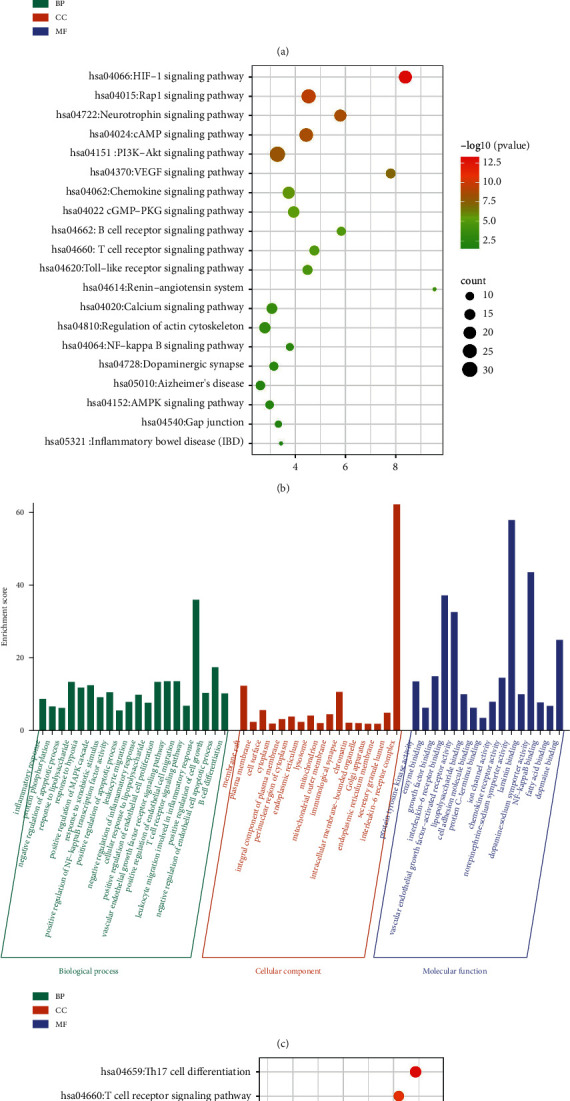
Functional and pathway enrichment analysis. (a) GO functional enrichment of XNJ targets related to IS; (b) KEGG pathway analysis of XNJ targets related to IS; (c) GO functional enrichment of XNJ targets related to IBD; (d) KEGG pathway analysis of XNJ targets related to IBD.

The functional bioinformatics analysis was performed to better elucidate the molecular mechanism of XNJ (Figure 5). Among the common targets of XNJ-IS, a total of 908 GO functions was enriched in the common targets (including 659 biological process items, 91 cell component items, and 158 molecular function items). In addition, there were 117 enrichment target pathways, which mainly related to the VEGF signaling pathway, NF-kappa B signaling pathway, gap junction, and inflammatory bowel disease. Among the common targets of XNJ-IBD, a total of 1166 GO functions were enriched in the common targets (including 869 biological process items, 103 cell component items, and 194 molecular function items). In addition, there were 162 enrichment target pathways, which mainly related to the T cell receptor signaling pathway, NF-kappa B signaling pathway, gap junction, and epithelial cell signaling in *Helicobacter pylori* infection.

### 3.4. XNJ Reduced Cerebral Infarction Area and Alleviated Pathological Damage of Brain Tissue in Stroke Mice

**Figure 6:**
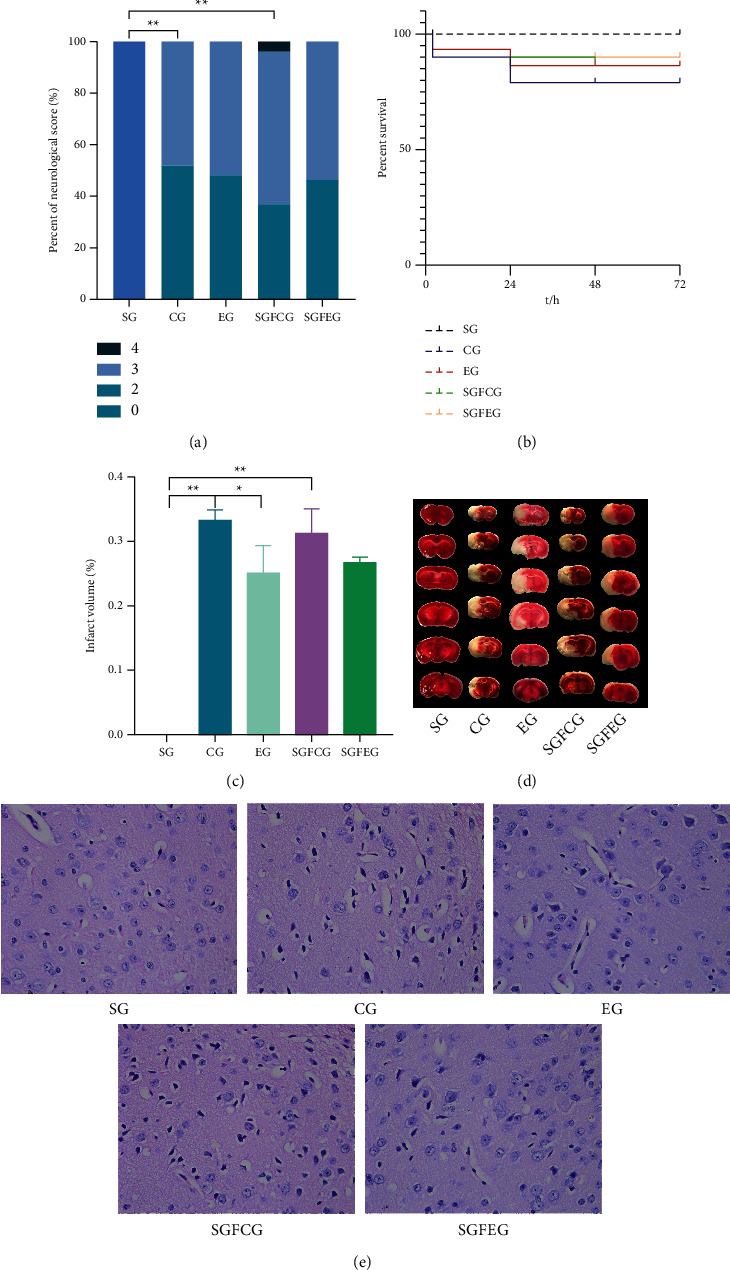
XNJ improved nerve function, alleviated cerebral infarction, and reduced the apoptosis of nerve cells of tMCAO mice. (a) Neurological scores; (b) survival probability; (c) cerebral infarct volume percentage; (d) TTC staining; (e) HE staining.

The neurological function scores of mice were evaluated 2 hours after modeling, and the results showed that the neurological function scores of mice in each group were higher than those of SG (*p* < 0.01, Figure 6(a)), indicating that modeling was successful. Survival analysis showed that there was no significant difference in the survival curve of mice in each group (*p* > 0.05, Figure 6(b)). The mortality of mice in each group was 0% (SG), 19.35% (CG), 12.90% (EG), 12.90% (SGFCG), and 9.68% (SGFEG). Compared with SG, the area of cerebral infarction in mice in the CG and SGFCG was significantly increased (*p* > 0.01, Figures 6(c), 6(d)). Compared with CG, the area of cerebral infarction in mice in the EG was significantly reduced (*p* < 0.05, Figures 6(c), 6(d)). Compared with SGFCG, the cerebral infarction area in the SGFEG was not significantly reduced (*p* > 0.05, Figures 6(c)and 6(d)). HE staining showed that the morphology of neurons in the ischemic cerebral cortex of CG and SGFCG was incomplete, with the disorder of arrangement, degeneration, loss of neurons, nuclear pyknosis, and enlarged space around cells, while the morphology of neurons in EG and SGFEG was significantly improved, with more complete morphology, more orderly arrangement, and less degeneration of neurons (Figure 6(e)).

### 3.5. XNJ Reduced Inflammation in the Brains of Stroke Mice

**Figure 7:**
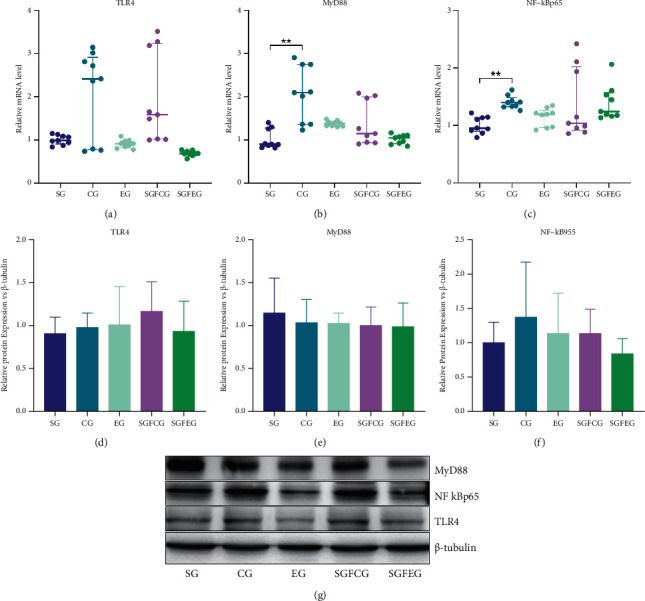
XNJ reduced inflammation in the brains of stroke mice. (a) Relative mRNA level of TLR4; (b) relative mRNA level of MyD88; (c) relative mRNA level of NF-kBp65; (d) relative protein expression of TLR4 vs. *β*-tubulin; (e) relative protein expression of MyD88 vs. *β*-tubulin; (f) relative protein expression of NF-kBp65 vs. *β*-tubulin; (g) representative blots of the protein levels.

RT-PCR and Western blot were used to detect the expression of inflammatory factors in the cerebral cortex of the ischemic side of mice. The results showed that the mRNA expressions of TLR4 (*p* > 0.05, Figure 7(a)), MyD88 (*p* > 0.05, Figure 7(b)), and NF-KBP65 (*p* > 0.05, Figure 7(c)) in the ischemic cortex of EG showed a decreasing trend compared with CG, and the protein expressions of MyD88 (*p* > 0.05, Figure 7(e)) and NF-KBP65 (*p* > 0.05, Figure 7(f)) were consistent with this trend. Compared with SGFCG, the mRNA and protein expressions of TLR4, MyD88, and NF-KBP65 showed a decreasing trend in SGFEG (*p* > 0.05, Figures 7(a)–7(f)).

### 3.6. XNJ Improved BBB Function in Stroke Mice

**Figure 8:**
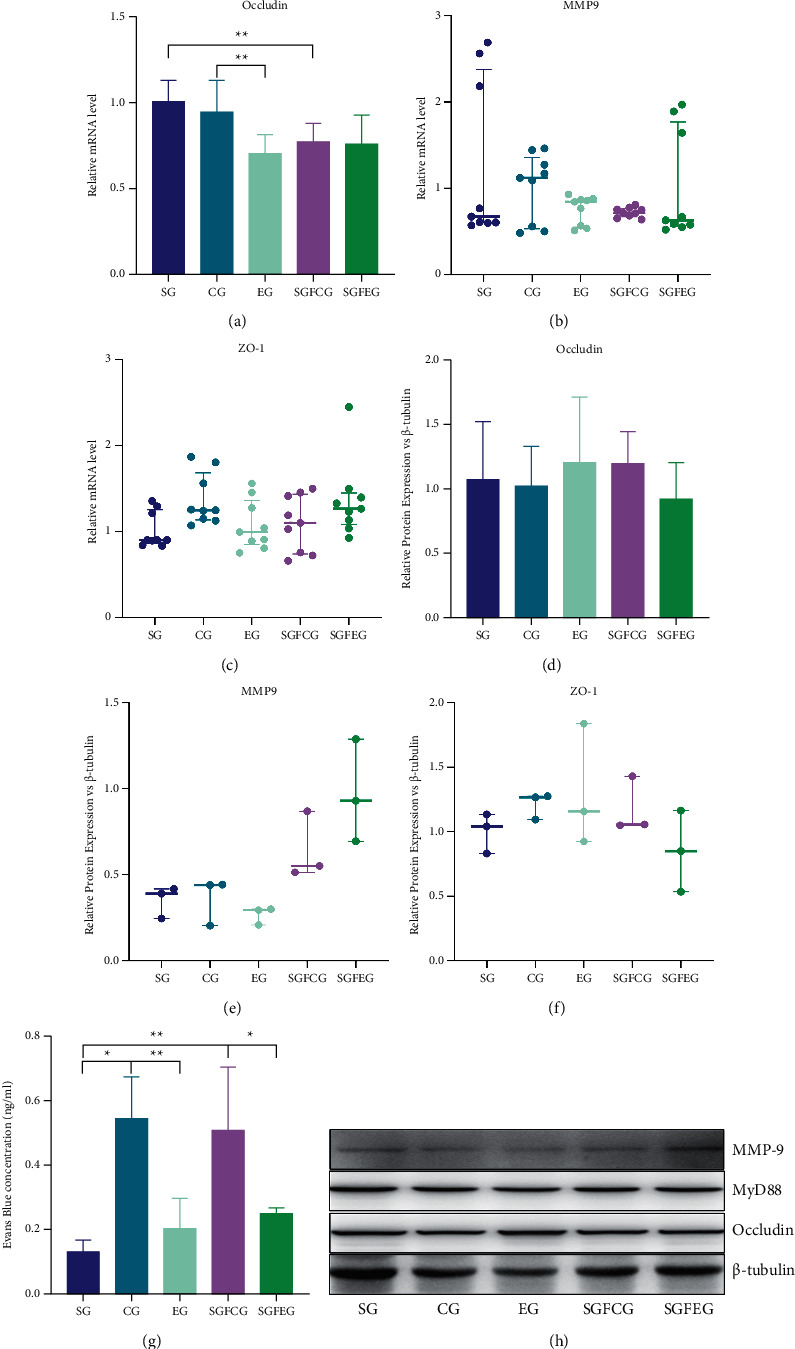
XNJ improved blood-brain barrier function in stroke mice. (a) Relative mRNA level of occludin; (b) relative mRNA level of MMP9; (c) relative mRNA level of ZO-1; (d) relative protein expression of occludin vs. *β*-tubulin; (e) relative protein expression of MMP9 vs. *β*-tubulin; (f) relative protein expression of ZO-1 vs. *β*-tubulin; (g) Evans blue concentration; (h) representative blots of the protein levels.

Compared with CG, the expression of occludin mRNA in the EG was significantly decreased (*p* < 0.01, Figure 8(a)), while the expression of occludin protein was increased (*p* > 0.05, Figure 8(d)). The mRNA and protein expressions of MMP9 (*p* > 0.05, Figures 8(b), 8(e)) and ZO-1 (*p* > 0.05, Figures 8(c), 8(f)) showed a decreasing trend. Compared with SGFCG, the mRNA and protein expression of occludin in SGFEG showed a decreasing trend (*p* > 0.05, Figures 8(a), 8(d)). BBB permeability assay showed that the concentration of EB in EG (*p* < 0.01, Figures 8(g)) and SGFEG (*p* < 0.05, Figure 8(g)) was significantly lower than that in the corresponding model group.

### 3.7. XNJ Affected the Immune Response in the Gut of the Stroke Mice

**Figure 9:**
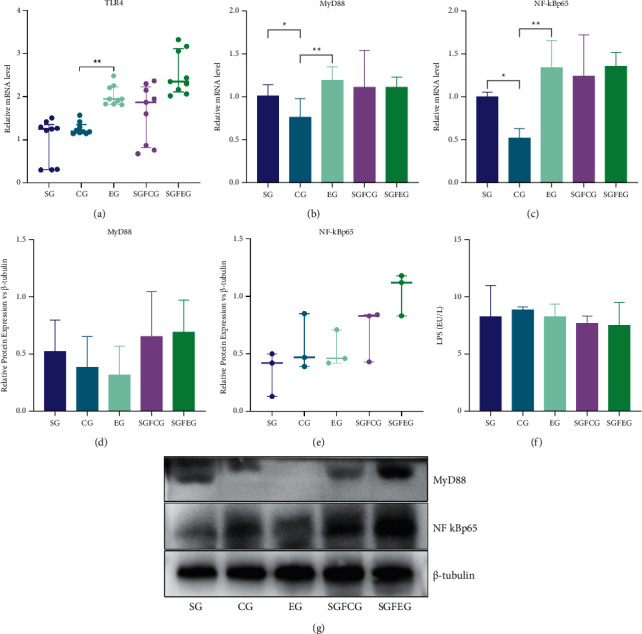
XNJ affected the immune response in the gut of the stroke mice. (a) Relative mRNA level of TLR4; (b) relative mRNA level of MyD88; (c) relative mRNA level of NF-kBp65; (d) relative protein expression of MyD88 vs. *β*-tubulin; (e) relative protein expression of NF-kBp65 vs. *β*-tubulin; (f) the content of plasma LPS; (g) representative blots of the protein levels.

Compared with CG, the mRNA expressions of TLR4, MyD88, and NF-kBp65 in the colon of EG were significantly increased (*p* < 0.01, Figures 9(a)–9(e)), while the expression of these factors was not significantly different between SGFCG and SGFEG (*p* > 0.05, Figures 9(a)–9(e)). Compared with the corresponding model group, the content of plasma LPS in EG and SGFEG showed a decreasing trend (*p* > 0.05, Figure 9(f)).

### 3.8. XNJ Improved Intestinal Mucosal Barrier Function in Stroke Mice

**Figure 10:**
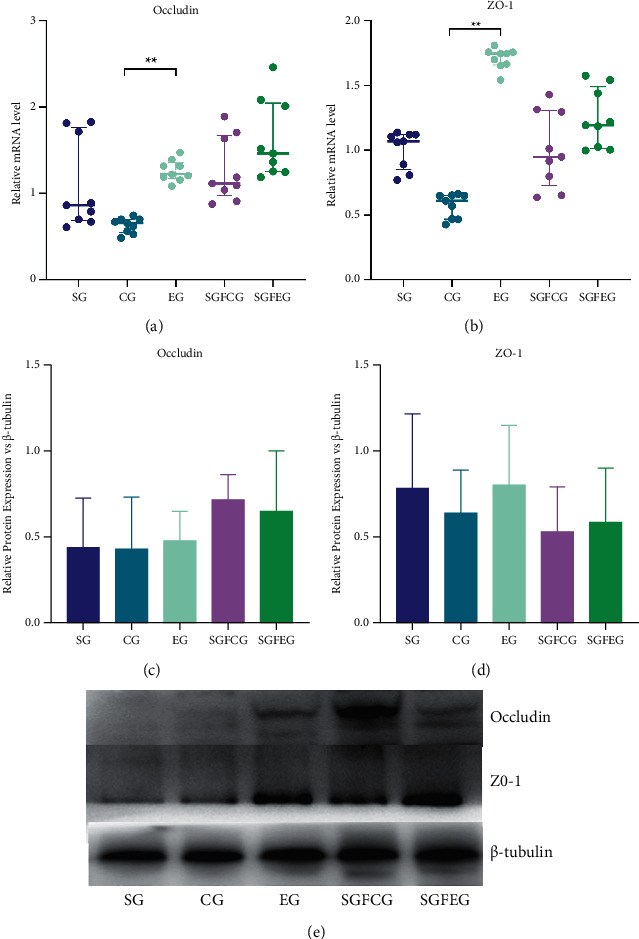
XNJ improved intestinal mucosal barrier function in stroke mice. (a) Relative mRNA level of occludin; (b) relative mRNA level of ZO-1; (c) relative protein expression of occludin vs. *β*-tubulin; (d) relative protein expression of ZO-1 vs. *β*-tubulin; (f) representative blots of the protein levels.

Compared with CG, the mRNA expression of occludin (*p* < 0.01, Figure 10(a)) and ZO-1 (*p* < 0.01, Figure 10(b)) in the colon of the EG was significantly increased, and the protein expression also showed a similar trend, while there was no significant difference between SGFCG and SGFEG (*p* > 0.05, Figure 10).

### 3.9. Effects of XNJ on Intestinal Microflora of Stroke Mice

#### 3.9.1. Data Quality Control and Statistics

The data quality is shown in Table 6.

The Q20% of raw reads was 98.05%, and the Q30% of raw reads was 94.19%. After removing the adapter sequences and primers from the sequences and filtering low-quality sequences, we obtained clean reads. The Q20% of the raw reads was 99.21%, and the Q30% of raw reads was 96.63%, which met the quality requirements.

#### 3.9.2. Alpha Diversity

**Figure 11:**
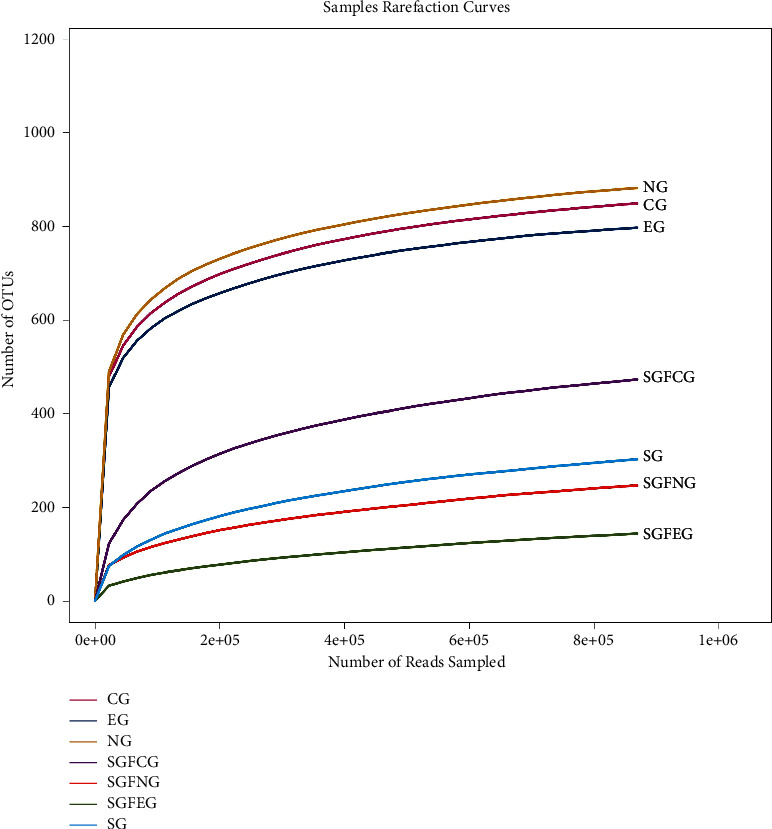
Rarefaction curves for all groups.

Rarefaction curve analysis (Figure 11) showed that as the number of extracted sequences increased, it reached a stable level, indicating that the sequencing depth had covered rare new phylotypes and most of the diversity. In addition, the numbers of OTUs were different between groups. The sham germ-free groups (SGFCG, SGFEG, and SGFNG) had fewer kinds of OTUs than the other groups, which demonstrated that it is possible to construct sham germ-free models treated with antibiotics.

**Figure 12:**
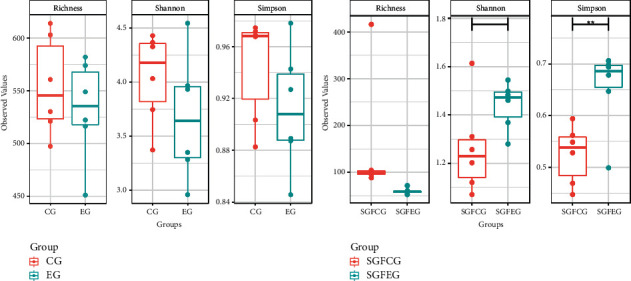
Richness, Simpson, and Shannon indexes between the XNJ and control groups (^∗∗^*p* < 0.01 between the two groups).

**Figure 13:**
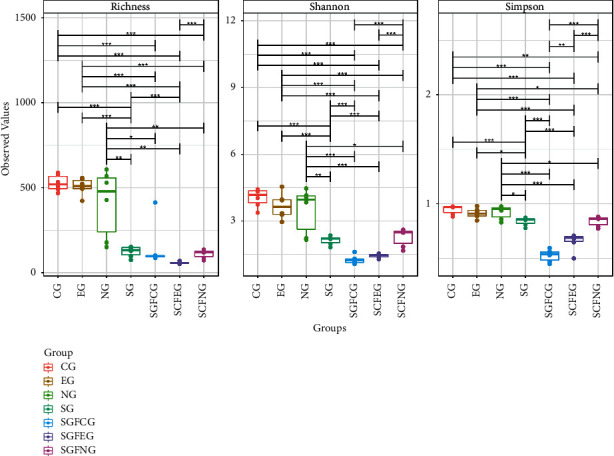
Richness, Simpson, and Shannon indexes in all groups (^*∗*^*p* < 0.05, ^∗∗^*p* < 0.01, ^∗∗∗^*p* < 0.001 between the two groups).

Gut microbiota diversity and richness were evaluated by the species richness index, Simpson index, and Shannon index. The results between the CG, SGFCG, EG, and SGFEG are shown in Figure 12, and the results of all of the groups are shown in Figure 13.

The differences in diversity and richness between the CG and EG were not significant. The Simpson index of the SGFEG was significantly greater than that of the SGFCG, but the richness of the SGFCG was greater than that of the SGFEG. When all groups were compared, there were significant differences between the sham germ-free group and the other groups, with lower alpha diversity in the sham germ-free group. Moreover, there were no significant differences in alpha diversity between the model and the normal group.

#### 3.9.3. Beta Analysis

**Figure 14:**
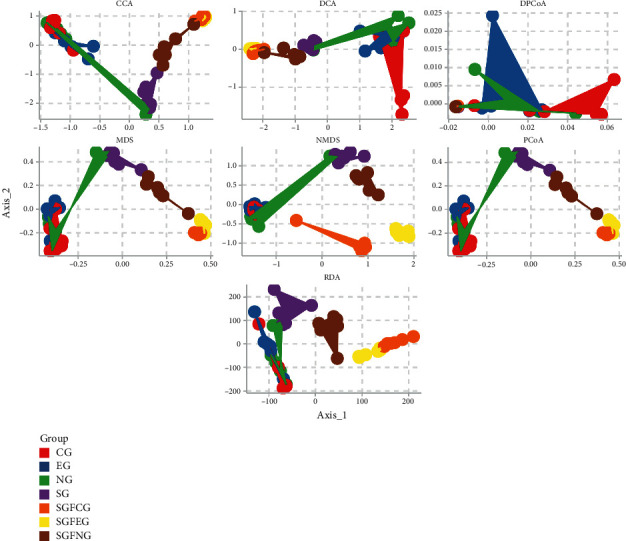
PCoA, DPCoA, DCA, CCA, NMDS, MDS, and RDA for all groups.

We used several different algorithms to try to separate groups. PCoA, DPCoA, DCA, CCA, NMDS, MDS, and RDA were performed to compare the overall microbiota structures in each group (Figure 14).

CG and EG clusters could be divided into two groups by DPCoA. For most algorithms, the SGFCG and SGFEG could be divided. These results suggested that XNJ injection changed the overall gut microbiota composition between the CG, SGFCG, EG, and SGFEG and that these groups could be divided by some algorithms.

**Figure 15:**
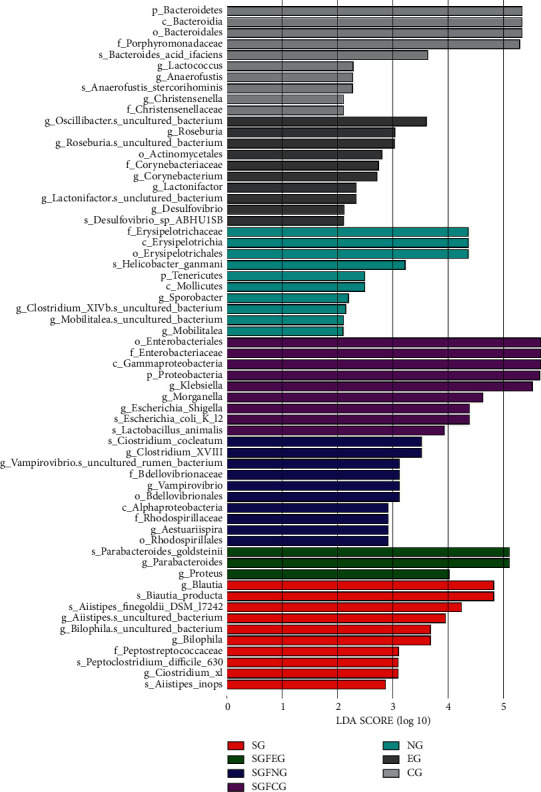
LEfSe analysis at the genus level.

We also performed LEfSe analysis to identify the specific bacterial genera that were characteristic among the 7 groups. The results are shown in Figure 15.

#### 3.9.4. Community Composition Analysis

We compared community composition between the SGFCG and SGFEG, EG, and CG at the phylum, family, and genus levels to identify the primary types of gut microbiota that were differentially expressed between the CG, SGFCG, EG, and SGFEG.

**Figure 16:**
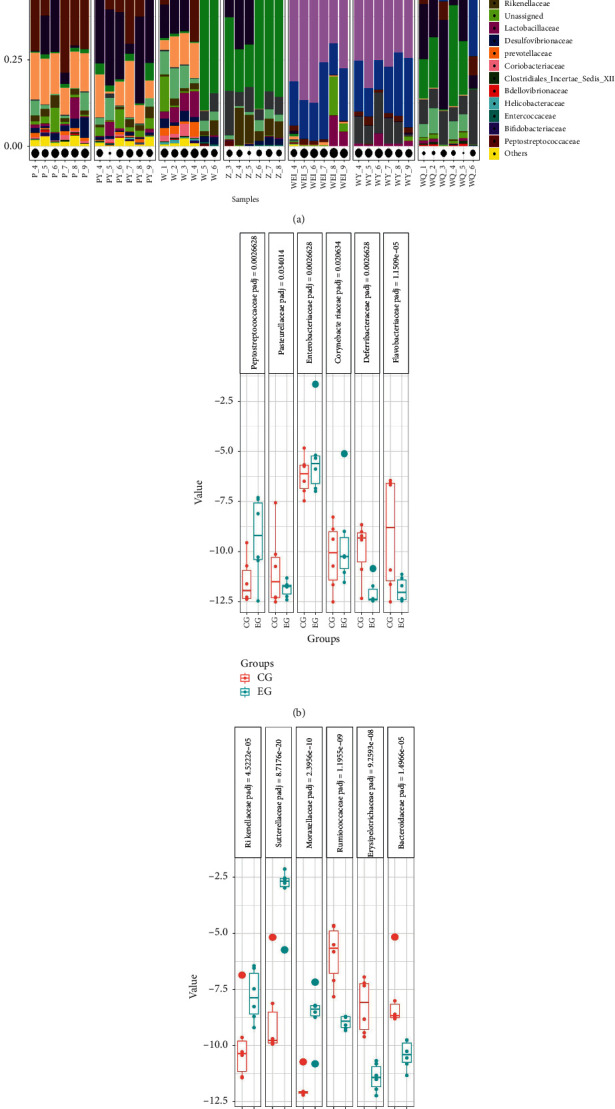
Community composition at the family level and significantly different bacteria with adjusted *p* values. (a) Community compositions at the family level in all groups; (b) significantly different bacteria between the CG and EG; (c) significantly different bacteria between the SGFCG and SGFEG (we set the *p* adjusted threshold between the SGFCG and SGFEG to 0.001 and the *p* adjusted threshold between the EG and CG to 0.05).

At the family level, as indicated in Figure 16(a), while the EG and CG mice and the SGFCG and SGFEG mice presented very similar gut microbiota compositions, XNJ significantly affected the relative abundances of certain families. The differences in the gut microbiota at the family level are shown in Figures 16(b) and 16(c). Specifically, there were significant differences in *Bacteroidaceae*, *Rikenellaceae*, *Ruminococcaceae*, *Erysipelotrichaceae*, *Moraxellaceae*, and *Sutterellaceae* between the SGFCG and SGFEG, with *Sutterellaceae* being a dominant microbiota family in the SGFCG and SGFEG. *Enterobacteriaceae*, *Peptostreptococcaceae*, *Flavobacteriaceae*, and *Deferribacteraceae* were significantly different between the CG and EG, while none of these families were dominant in CG and EG. *Enterobacteriaceae*, *Lachnospiraceae*, *Porphyromonadaceae*, *Verrucomicrobiaceae*, *Bacteroidaceae*, and *Ruminococcaceae* were dominant families in all of the samples. We also found that there were robust differences between germ-free mice and normal mice. In addition to the above results, we found that XNJ regulated the abundance of *Sutterellaceae* in the SGFCG and SGFEG.

**Figure 17:**
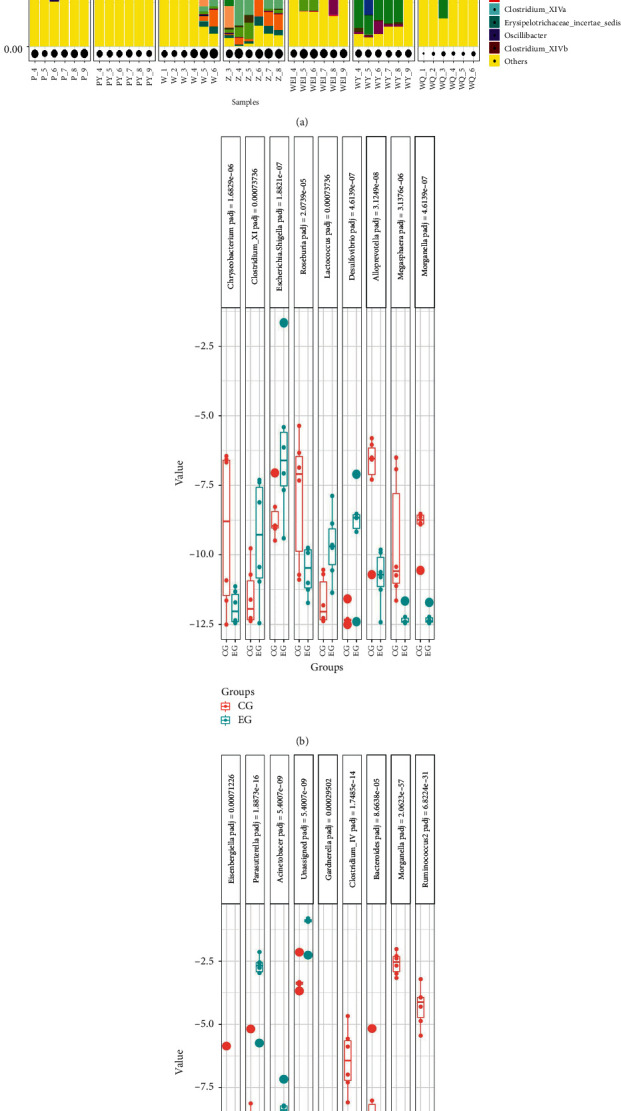
Community composition at the genus level and significantly different bacteria with *p* adjusted values. (a) Community compositions at the genus level of all groups; (b) significantly different bacteria between the CG and EG; (c) significantly different bacteria between the SGFCG and SGFEG (we set the *p* adjusted threshold between the SGFCG and SGFEG to 0.001 and the *p* adjusted threshold between the EG and CG to 0.001).

At the genus level, the different kinds of gut microbiota were as follows (Figures 17(b) and 17(c)). The components of the gut microbiota at the genus level are shown in Figure 7(a). There were robust differences between *Morganella and Parasutterella* between the SGFCG and SGFEG. *Morganella* levels decreased after Xingnaojing injection, while *Parasutterella* levels increased. *Akkermansia* was the main component of the gut microbiota in the EG, and there were some differences between the CG and EG (*p* adjust = 0.45). *Morganella* was downregulated in both groups.

**Figure 18:**
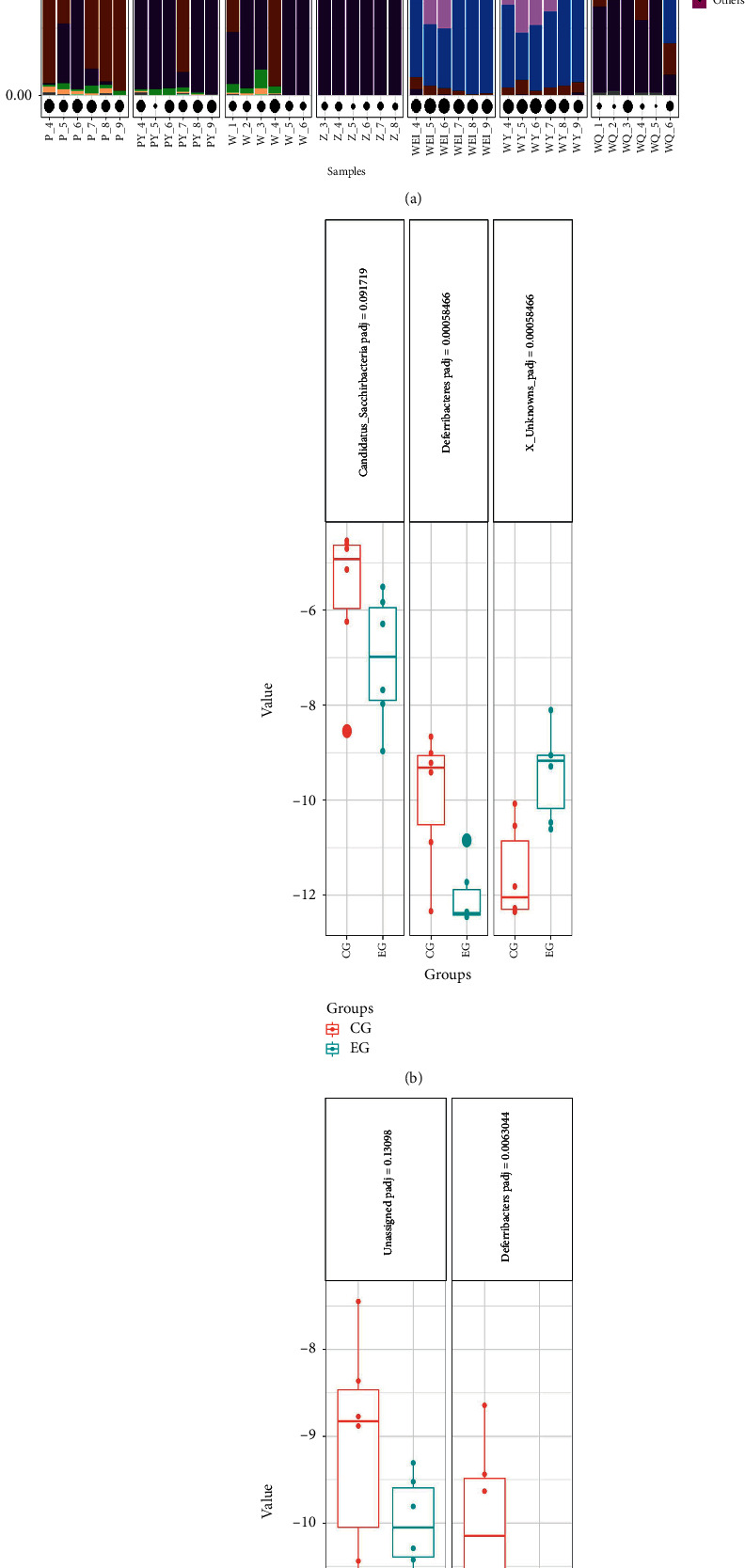
Community composition at the phylum level and significantly different bacteria with *p* adjusted values. (a) Community compositions at the phylum level in all groups; (b) significantly different bacteria between the CG and EG; (c) significantly different bacteria between the SGFCG and SGFEG (we set the *p* adjusted threshold between the SGFCG and SGFEG to 0.15 and the *p* adjusted threshold between the EG and CG to 0.15).

At the phylum level, two kinds of bacteria were different between the CG and EG, while one kind of bacteria was different between the SGFCG and SGFEG (*p* adjusted <0.15). *Firmicutes*, *Bacteroidetes*, and *Verrucomicrobia* were the most common components of the gut microbiota in CG and EG. In the SGFCG and SGFEG, *Proteobacteria*, *Firmicutes*, and *Bacteroidetes* were most common. *Deferribacteres* levels were decreased in both groups (Figure 18).

#### 3.9.5. Functional Prediction

**Figure 19:**
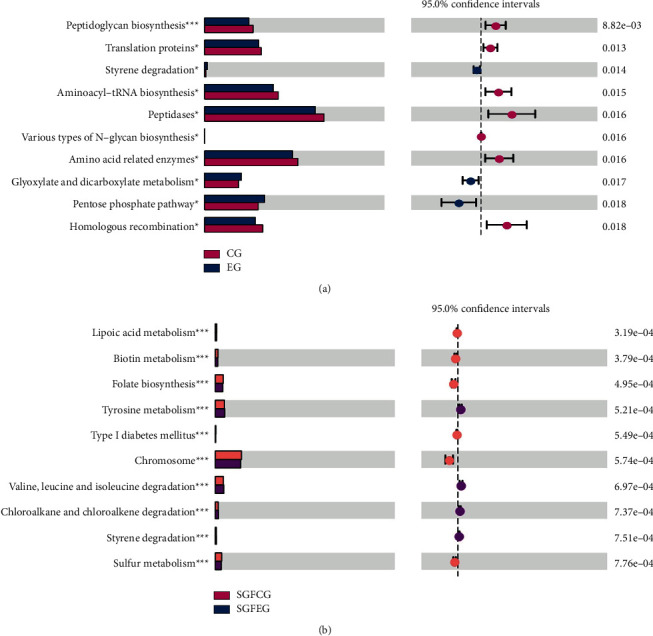
Differential enrichment of KEGG pathways. (a) Differential enrichment of KEGG pathways between the CG and EG and (b) differential enrichment of KEGG pathways between the SGFCG and SGFEG (^*∗*^*p* < 0.05, ^∗∗^*p* < 0.001, and ^∗∗∗^*p* < 0.0001 between the two groups).

We used PICRUSt to predict the functions of gut bacteria, and differentially enriched KEGG pathways between the XNJ (EG and SGFEG) and control groups (CG and SGFCG) were identified (Figure 19). We found that peptidoglycan biosynthesis was the most different between CG and EG. Lipoic acid metabolism, antigen processing and presentation, progesterone-mediated oocyte maturation, and transporters were the most different between the SGFCG and SGFEG.

#### 3.9.6. Analysis of SCFAs in the Feces

**Figure 20:**
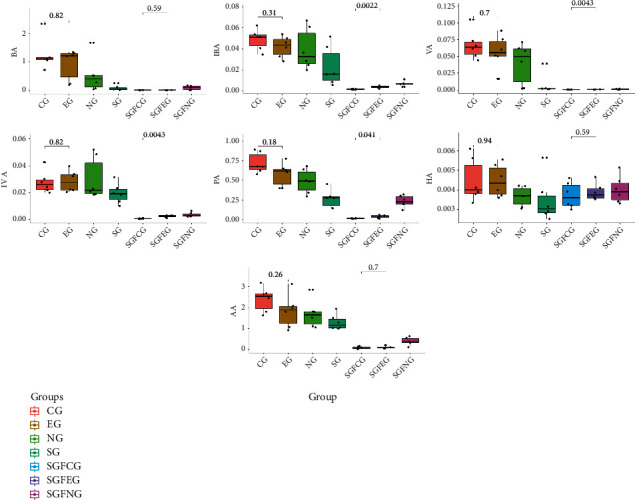
Plots of the SCFA levels in the feces in all groups (VA: valeric acid/valerate, IVA: isovaleric acid/isovalerate, IBA: isobutyric acid/isobutyrate, HA: caproic acid/caproate, AA: acetic acid/acetate, PA: propionic acid/propionate, and BA: butyric acid/butyrate); *p* values between the CG and EG and between the SGFCG and SGFEG are indicated in the plots.

We evaluated the levels of SCFAs in feces for all groups (Figure 20). We found that the SCFA levels in the sham germ-free groups were lower than those in the other groups. Moreover, XNJ increased the levels of PA, VA, IBA, and IVA in the SGFEG (*T*-test, *p* value <0.05). SCFA levels were not significantly different between the EG and CG.

## 4. Discussion

Ischemic stroke (IS) is the most common type of stroke and is caused by thrombotic or embolic occlusion and stenosis of a cerebral artery due to sudden loss of blood circulation to an area of the brain, resulting in a corresponding loss of neurologic function. It has a high incidence, high disability rate, and high mortality rate [32, 33]. It has been confirmed that IS is closely related to intestinal flora [8–11]. Previous studies have shown that XNJ, which is widely used for stroke in China, could protect intestinal mucosal barrier function and affect immune response [24, 25]. In this study, we first conducted a network pharmacological analysis to discover relevant pathways for the effects of XNJ on IS and IBD, and then, we continued to conduct relevant experimental validation. The results showed that XNJ improved the neurological function of tMCAO mice, changed the intestinal flora and its metabolites of tMCAO mice, and showed that the brain-protective effect of XNJ was partly affected by affecting intestinal flora.

In the section on animal experiments, we generated a middle cerebral artery occlusion (tMCAO) mouse model as a model of IS to explore the mechanism underlying the effect of XNJ on IS from the perspective of the intestinal microbiota. A sham germ-free mouse model was established by antibiotic treatment. Germ-free (GF) mouse models are generally considered to be the gold standard for studies of microbiota [34]. The use of antibiotic treatments to construct a sham germ-free group is an alternate method that is now relatively well established for studies of gut microbiota [35]. This approach does have the disadvantage of causing other damage in animals since it is usually impossible to narrow down the effects of antibiotics to specific changes in the composition of the intestinal microbiota. However, compared with GF mice, antibiotic treatment could manipulate the intestinal microbiota of animals without inducing developmental changes [36]. Depleting the gut microbiota with a mixture of broad-spectrum antibiotics to mimic the sterile gut of sterile (GF) mice has become an appropriate strategy for exploring the effects of the gut microbiota under pathological conditions [37, 38]. Although the mice on antibiotics are not completely cleared of bacteria, the bacterial load was significantly reduced [34]. In addition, studies have shown that ad libitum administration of antibiotics depletes the gut microbiota more effectively than direct oral gavage [39]. That could control variables and result in more obvious results, which was consistent with the previous research results of our team [28]. In this study, *α* diversity and SCFAs content in the sham germ-free group (SGFCG, SGFEG, and SGFNG) were low, which demonstrated that it is possible to construct sham germ-free models treated with antibiotics.

The results of network pharmacological analysis show that the pathway of XNJ treatment IS is mainly related to the VEGF signaling pathway, NF-kappa B signaling pathway, gap junction, etc. Rong Ma also found that XNJ inhibited the inflammatory response by reducing the expression of TNF-*α*, IL-6, and IL-1*β* [20], and Xiao et al. found that XNJ relieved BBB destruction by upregulating the expression of junction protein, occludin, and ZO-1 [22]. The experimental part of this study also showed that XNJ significantly improved the neural function of tMCAO mice, reduced the area of cerebral infarction, reduced inflammation-related factors such as TLR4, MyD88, NF-kappa B in ischemic site, and significantly reduced BBB permeability.

It is worth noting that through network pharmacological analysis, we found that XNJ also has an impact on IBD, and the main pathway involved the T cell receptor signaling pathway, NF-kappa B signaling pathway, gap junction, etc. Guo and Liu found that XNJ reduced DAO and L/M and protected intestinal mucosal function [24]. Wang et al. found that XNJ prevented endotoxins from penetrating the intestinal mucosa into the circulatory system [25]. This study also showed that after removing most of the intestinal flora of mice with antibiotics, the cerebral protective effect of XNJ was weakened, and the area of cerebral infarction in mice was not significantly reduced. XNJ improved the intestinal mucosal barrier function by significantly increasing the expression of occludin and ZO-1 in the intestines of tMCAO mice, while this effect was weakened in the sham germ-free group. The above results suggest that the intestinal flora affected the protective effect of XNJ on the brain.

Regarding alpha diversity, we found that the difference in diversity was not significant in the CG and EG but that the diversity of the EG was lower than that of the CG overall. Previous studies have indicated that IS patients and IS models exhibit no significant differences in alpha diversity compared with the control group [10, 40, 41]. A previous study of the effect of the combination of the Chinese medicines Puerariae Lobatae Radix and Chuanxiong Rhizoma on the mechanism of gut microbiota in cerebral ischemic stroke found that the injection group had a significantly decreased diversity compared with that of the control group [42, 43] and that the trends in alpha diversity were in accordance with those observed between the CG and EG. However, in the SGFCG and SGFEG, the diversity of the XNJ group was greater than that of the control group, which contradicted the results obtained for the CG and EG, indicating that XNJ can decrease the high diversity in the CG and increase the low diversity in the SGFCG. We hypothesized that XNJ can bidirectionally regulate the alpha diversity of the gut microbiota, which should be further researched.

At the family level, we found in the EG, XNJ reduced the level of *Flavobacteriaceae* and *Deferribacteraceae*, both of which were pathogenic. Among them, *Flavobacteriaceae* was found to be significantly increased in diabetic rats [44], while *Deferribacteraceae* could induce colitis [45]. XNJ increased the level of *Sutterellaceae* in the SGFEG compared with the SGFCG. *Sutterellaceae* is present in abundance in the healthy human colon and is a member of the indigenous intestinal microbiota and can be isolated from both the intestinal tract and infections of gastrointestinal origin [46]. The specific role of *Sutterellaceae* species in the human gut is unclear. However, the *Sutterella* species has been shown to have mild proinflammatory abilities and is closely associated with inflammatory bowel disease [47]. The ability of Sutterella spp. to adhere to intestinal epithelial cells suggests that they may have immunomodulatory effects [47].

At the genus level, XNJ increased the level of *Akkermansia* in EG. Currently, there are few studies on *Akkermansia* genus, but studies have found that *Akkermansia muciniphila* is a promising probiotic [48], which could enhance the integrity of intestinal epithelial cells and the thickness of mucous layer, thus promoting intestinal health [49, 50]. In addition, the level of *Parasutterella* increased in the SGFEG. *Parasutterella* was found to be associated with irritable bowel syndrome and chronic intestinal inflammation [51].

At the phylum level, *Deferribacteres* were decreased in both the EG and SGFEG. In Runzhi Chen's study on Puerariae Lobatae Radix and Chuanxiong Rhizoma [42], the Chinese medicines could also regulate the gut microbiota by reducing *Deferribacteres*, a phylum of gram-negative bacteria that makes energy through anaerobic respiration [52]. Another study showed a link between the accumulation of *Deferribacteres* and colitis [53].

In conclusion, XNJ increased probiotics and decreased pathogenic bacteria in EG, while this trend was not obvious in the SGFEG, and even worse, the level of some pathogenic bacteria increased in the SGFEG.

Analysis of SCFAs in the feces showed that XNJ increased the levels of PA (propionate), VA (valerate), IBA (isobutyrate), and IVA (isovalerate) in the feces of the SGFEG. SCFAs are generated by bacterial fermentation of polysaccharides [54], and the composition of an individual's microbiota and the presence of keystone species also influence fiber fermentation [55]. Thus, we concluded the XNJ increased the community diversity in the SGFEG to induce this phenomenon. Rose et al. [56] suggested that bacterial metabolites (SCFAs) may affect mitochondrial function to elicit mitochondrial dysfunction-induced cecal gut dysmotility and that mitochondrial dysfunction can cause gut dysmotility. For example, butyrate is converted into acetyl-CoA, which is then utilized in the citric cycle for NADH production, and NADH is an important substance for mitochondrial function [57]. Therefore, we believe that XNJ can improve IS prognosis, partly by regulating SCFA levels in the intestine.

Because of the short nature of the experiment, this study only clarified the correlation between intestinal flora and the prognosis of IS, but their cause-and-effect relationship has not been proven, and further, fecal bacteria transplantation experiments are needed to elucidate the specific mechanism. Second, the functions of selected different bacteria should be further observed, possibly by planting specific bacteria to explore their functions.

## 5. Conclusion

In conclusion, IS causes dysbiosis of some specific bacteria in the gut microbiota. XNJ is an effective treatment for IS, and its mechanism was related to improving intestinal barrier function and regulating intestinal flora and SCFAs. Network pharmacology revealed that XNJ acts through multiple targets and multiple pathways.

## Figures and Tables

**Table 1:** Components of Xingnaojing injection.

Traditional Chinese medicine name	*Moschus*	*Radix Curcumae*	*Borneolum Syntheticum*	*Fructus gardeniae*
Binomial nomenclature names	Not plant	*Curcuma aromatica Salisb*	Not plant	*Gardenia jasminoides J.Ellis*
Part	Not applicable	Root	Not applicable	Fruit
Compatibility proportion	7.5	30	1	30

**Table 2:** Chromatographic conditions of positive ions.

Time (min)	Parameter
0	A : 5% B : 95%
5	A : 70% B : 30%
7	A : 100% B : 0%
9	A : 100% B : 0%
10	A : 5% B : 95%
12	A : 5% B : 95%

**Table 3:** Main reagents and equipment.

Name	Company	Cat#	Lot#
Activated carbon powder	Shanghai Hushi Laboratorial Equipment Co., Ltd.	10006619	20190612
TTC powder	Beijing Solarbio Science and Technology Co., Ltd.	T8100	20190314
Vancomycin hydrochloride	Sigma	E8010	1112044
Metronidazole	Sigma		
Evans blue	Sigma	443-48-1	M1574
Neomycin sulfate	Sigma	1405-10-3	N6386
Ampicillin sodium salt	Sigma	69-52-3	A9158
FITC	Ruixi Biotech Co., Ltd	R-FD-001-4K	RA0190902
Isoflurane	Ruiwode Lift Technology Co., Ltd.		20190711
Anesthesia respirator	Beijing Zhongshidichuang Science and Technology Development Co., Ltd.	ZS-MV-IV	2.02*E* + 13
Feed for growth and reproduction of mice	SPF Biotechnology Co., Ltd.		0527SF0604A
Universal tissue fixator (neutral)	Wuhan Servicebio Technology Co., Ltd.	G101	
Gum acacia powder	Sinopharm Chemical Reagent Co., Ltd.	69012484	20181212

**Table 4:** The 7 groups and their interventions.

Group	Abbreviation	*n*	Intervention	Processing time	Surgical approach
Normal group	NG	6	Normal saline injection	5 days	No surgery
Sham surgery group	SG	12	Normal saline injection	5 days	Isolation of the artery with no occlusion
tMCAO group (control group)	CG	24	Normal saline injection	5 days	tMCAO
Experiment group	EG	24	XNJ (10 ml XNJ and 20 ml normal saline)	5 days	tMCAO
Sham germ-free normal group	SGFNG	6	Normal saline injection	5 days	No surgery
Sham germ-free control group	SGFCG	24	Normal saline injection	5 days	tMCAO
Sham germ-free experiment group	SGFEG	24	XNJ (10 ml XNJ and 20 ml normal saline)	5 days	tMCAO

*Notes*. The mice were injected abdominally once a day for 5 days (156 ul/10 g), from 2 days before modeling to 3 days after modeling.

**Table 5:** Sequences of RT-qPCR primers.

Gene	Primer	Primer sequence (5′–3′)
MMP9	Forward	5′-TGTGCGTTATGGTTCAGGTCAGAC-3′
	Reverse	5′-GACTGCCAGGAAGACACTTGGTTATC-3′

MyD88	Forward	5′-AGGACAAACGCCGGAACTTTT-3′
	Reverse	5′-CTGTTCTAGTTGCCGGATCATC-3′

Occludin	Forward	5′-GCTATGGCTATGGCGGATATACAGAC-3′
	Reverse	5′-ACTAAGGAAGCGATGAAGCAGAAGG-3′

NF*κ*Bp65	Forward	5′-GAAGCACAGATACCACCAAGACACA-3′
	Reverse	5′-AGGTCAGCCTCATAGTAGCCATCC-3′

ZO-1	Forward	5′-GACCTTGAGCAGCCGTCATACAG-3′
	Reverse	5′-CCGTAGGCGATGGTCATAGTTCC-3′

TLR4	Forward	5′-AAATGCACTGAGCTTTAGTGGT-3′
	Reverse	5′-TGGCACTCATAATGATGGCAC-3′

GAPDH	Forward	5′-AGAAGGTGGTGAAGCAGGCATCT-3′
	Reverse	5′-CGGCATCGAAGGTGGAAGAGTG-3′

**Table 6:** Mean data quality of the samples.

	Raw_reads	Q20 (%)	Q30 (%)	Merge	Merge (%)	Primer	Clean_reads	Clean_reads (%)	Q20 (%)	Q30 (%)
Samples	233087.8	98.05	94.19	230179	98.75	229118	214464.2	92.02	99.21	96.63

## Data Availability

The data sets used or analyzed during the current study are available from the corresponding authors upon reasonable request.

## Abbreviations

XNJ: Xingnaojing injection
tMCAO: Transient Middle cerebral artery occlusion
SCFAs: Short-chain fatty acids
IS: Ischemic stroke
GBA: Gut-brain axis
OTU: Operational taxonomic unit
VA: Valeric acid/valerate
IVA: Isovaleric acid/isovalerate
IBA: Isobutyric acid/isobutyrate
HA: Caproic acid/caproate
AA: Acetic acid/acetate
PA: Propionic acid/propionate
BA: Butyric acid/butyrate.

## Supplementary Materials

Supplementary Materials Supplementary Table S1: active ingredients of XNJ identified by UHPLC-MS. Supplementary Table S2: 507 compound-related targets. Supplementary Table S3: 1667 IS-related targets and 2074 IBD-related targets. Supplementary Table S4: 210 shared targets were identified as potential therapeutic targets of XNJ against IS. .
