# Supplementary material for: Uncovering the Mechanism of the Xingnaojing Injection against Ischemic Stroke Using a Combined Network Pharmacology Approach and Gut Microbiota Analysis
Source: Evid Based Complement Alternat Med. 2022 May 20;2022:5886698. doi: 10.1155/2022/5886698 (PMC9142292; doi:10.1155/2022/5886698)
Supplement: Supplementary Materials — Supplementary Table S1: active ingredients of XNJ identified by UHPLC-MS. Supplementary Table S2: 507 compound-related targets. Supplementary Table S3: 1667 IS-related targets and 2074 IBD-related targets. Supplementary Table S4: 210 shared targets were identified as potential therapeutic targets of XNJ against IS. . [file 5886698.f1.zip › 5886698.f1/S1 Active ingredients of XNJ identified by UHPLC-MS..docx]

**Supplementary Tables**

**Supplementary Table S1.** Active ingredients of XNJ identified by UHPLC-MS.

| RT (min) | Molecular formula | Precursor m/z | Reference m/z | Adduct | MS/ MS spectrum | Identification | Herb |
| --- | --- | --- | --- | --- | --- | --- | --- |
| 5.008633 | C21H20O12 | 465.1264 | 465.1028 | [M+H]+ |  | Hyperin | Moschus |
| 5.194617 | C2H2O4 | 90.97627 | 91.00261 | [M+H]+ |  | Oxalic acid | Radix curcumae |
| 6.231233 | C7H6O3 | 139.0472 | 139.039 | [M+H]+ | 69.03288:127 93.05135:127 93.05406:127 94.03245:257 121.03493:127 121.03959:260 121.04269:127 121.04424:130 121.0458:130 121.05045:127 121.10323:127 138.67609:127 139.01855:127 139.0435:127 139.04849:383 139.05515:127 | 4-Hydroxybenzoic acid | Radix curcumae |
| 6.986367 | C10H12N2O | 177.0862 | 177.1023 | [M+H]+ | 116.07328:127 131.05309:127 132.08719:127 134.10059:127 134.1104:255 135.08922:127 143.09357:127 159.9753:127 177.06331:127 | Serotonin | Radix curcumae |
| 7.022033 | C10H14O | 151.0938 | 151.1118 | [M+H]+ |  | Thymol | Radix curcumae |
| 7.217367 | C14H14O3 | 231.0794 | 231.1016 | [M+H]+ | 166.76456:127 173.08539:127 200.13879:127 214.14575:127 231.06911:140 231.07768:386 231.07983:133 231.08197:393 231.08412:552 231.08626:525 231.08841:678 231.09056:1077 231.0927:140 231.09485:269 231.09698:133 231.09914:130 231.10127:390 231.10342:253 231.10558:532 231.112:129 231.12488:130 231.41023:133 232.46307:140 233.16917:127 233.3351:129 236.26228:127 238.19846:129 259.02475:127 | Bis(4-Hydroxybenzyl) ether | Radix curcumae |
| 7.274517 | C4H8N2O3 | 133.0837 | 133.0608 | [M+H]+ |  | L-Asparagine | Radix curcumae |
| 7.285017 | C12H18O2 | 195.1182 | 195.138 | [M+H]+ | 77.04572:256 89.05977:256 89.0611:638 89.06243:258 89.06509:259 89.06643:130 89.06776:130 89.06909:129 89.07308:129 89.07442:127 89.07841:130 89.07974:129 89.08773:130 89.08907:259 89.09039:129 133.08141:127 133.08304:254 133.08466:130 133.09117:253 133.09769:127 133.15465:129 151.09233:127 151.101:127 156.41214:127 195.05768:127 195.08527:127 195.12271:127 | 6-Methyl-7-(3-oxobutyl)-bicyclo[4.1.0]heptan-3-one | Radix curcumae |
| 7.40135 | C9H20O5S | 241.1462 | 241.1104 | [M+H]+ | 91.10716:127 91.11794:127 133.65979:127 136.3894:127 139.12497:127 139.13162:127 158.94551:127 158.95619:127 181.0486:127 196.13303:127 223.10071:127 223.10703:127 223.20398:127 241.10535:127 241.16011:127 | Musclide B | Moschus |
| 7.40135 | C15H20O4 | 265.1391 | 265.1435 | [M+H]+ |  | Curcumenolactone C | Radix curcumae |
| 7.411684 | C15H18O3 | 247.1287 | 247.1329 | [M+H]+ | 230.07918:127 247.22737:253 | Zederone | Radix curcumae |
| 7.468184 | C15H20O6 | 297.1436 | 297.1333 | [M+H]+ | 85.12457:127 111.04004:127 127.1084:127 132.08235:127 148.03664:127 149.02361:127 149.03221:127 150.02422:380 150.03113:127 151.10796:127 154.98793:127 177.22858:127 211.10904:127 248.98741:127 253.15218:127 265.01248:127 265.02167:127 265.02396:127 267.00751:127 279.07596:127 279.11838:127 280.13522:127 280.14703:127 280.14938:127 281.03564:127 281.05219:127 281.06165:127 281.06638:127 281.06876:127 281.2225:127 297.19821:127 299.05936:127 299.07153:127 | Zedoarolide A | Radix curcumae |
| 7.468184 | C19H24O7 | 365.1667 | 365.1595 | [M+H]+ | 189.10001:127 286.12494:127 303.14557:127 303.17749:256 326.90305:127 326.92093:127 346.06802:256 346.07065:258 347.14758:390 347.15021:127 347.15549:129 347.15811:131 347.16074:132 347.16336:132 347.16599:131 347.17126:129 347.17389:264 347.17651:260 347.17914:524 347.18176:648 347.18439:390 347.18701:658 347.19492:393 347.19754:131 347.20279:262 347.21069:131 347.21857:263 347.22119:131 347.2291:129 347.23697:127 347.73404:127 350.50992:127 350.6156:127 350.75034:264 363.98132:127 363.99747:127 365.11002:265 365.11542:127 365.1235:261 365.14508:271 365.15854:275 365.16394:137 365.17203:275 365.17743:676 365.18011:129 365.1828:820 365.18552:1253 365.1882:1363 365.19089:423 365.1936:1781 365.19629:684 365.19901:285 365.20169:556 365.20438:542 365.20709:553 365.21246:272 365.21518:144 365.22327:130 365.23944:134 365.33112:127 365.67099:144 366.46188:127 369.72363:127 372.80096:137 390.9187:127 413.16605:127 416.28348:137 | Gibberellin A8 | Moschus |
| 7.515183 | C17H24O6 | 325.1958 | 325.1646 | [M+H]+ | 111.04894:127 114.06648:256 114.07402:127 157.5771:127 158.09113:127 158.18871:127 159.73985:127 187.16089:127 187.16667:127 202.10147:127 202.10948:129 202.11349:256 202.11551:256 202.11751:127 202.12553:127 204.0779:127 231.14401:127 284.05939:127 316.62317:129 324.97974:127 325.15271:127 325.20615:127 | (-)-Blumealactone C | Borneol |
| 7.515183 | C16H26O7 | 331.1404 | 331.1751 | [M+H]+ | 98.97729:127 98.99414:127 99.75362:127 125.03959:260 174.99127:257 196.0798:127 207.0556:256 223.08817:256 252.98846:267 283.18747:127 287.13284:257 331.13062:256 331.13577:137 331.13834:130 331.1409:130 331.14603:141 331.14859:385 331.15115:129 331.15372:674 331.15631:534 331.15887:827 331.16144:415 331.164:415 331.16656:397 331.16913:550 331.17169:137 331.17429:267 331.17685:419 331.17941:401 331.18454:133 331.1871:137 331.19223:137 331.19482:130 331.19995:130 336.62424:141 | Epijasminoside A | Fructus gardeniae |
| 7.530517 | C15H22O5 | 283.1678 | 283.154 | [M+H]+ | 89.05846:129 89.06112:258 89.06378:127 89.06778:127 89.06911:129 93.12208:129 133.08307:130 133.08469:130 133.08632:522 133.08957:262 133.0912:392 133.09283:131 133.09445:131 133.1026:127 133.10748:130 171.03293:127 177.10468:134 177.11218:127 177.11406:262 177.11781:399 177.12158:134 177.1366:127 177.16664:134 195.1405:131 227.20351:127 227.20564:127 239.15744:127 253.15005:127 253.1568:258 282.36185:127 283.16611:127 283.17325:258 283.1756:131 283.17798:384 283.18036:127 283.18274:258 283.18747:131 283.19461:127 283.22784:127 | Zedoarolide B | Radix curcumae |
| 7.566167 | C17H24O10 | 389.1652 | 389.1443 | [M+H]+ | 73.04946:127 73.05187:127 73.05791:127 73.06032:127 73.0772:127 89.05986:127 89.06918:127 89.07184:127 90.05857:127 96.80666:127 105.91356:127 115.05264:127 115.05415:127 115.05567:253 116.05513:253 116.05968:127 116.06273:127 123.07843:127 125.04115:127 133.08475:127 133.0929:253 133.09778:127 133.0994:127 134.08762:127 134.53726:127 149.05469:127 159.08441:253 159.09686:127 160.08427:127 160.08784:127 160.09499:127 160.09856:127 160.10034:127 164.92761:127 169.03139:261 178.11443:127 178.11819:380 203.10367:127 203.11171:127 203.11774:127 223.06711:127 246.13947:256 246.14169:127 247.13432:127 248.11342:127 248.15787:127 250.96169:127 266.98923:385 266.99387:259 267.01459:127 267.1322:264 267.98837:259 267.99301:129 267.99762:135 268.00455:270 268.00687:127 268.00916:127 268.01147:127 268.0184:127 268.02533:127 269.79291:135 269.98993:127 271.6756:127 271.9873:127 282.05377:127 282.06799:127 284.9924:127 286.00317:127 286.03183:129 286.03421:127 287.06833:260 328.19504:127 328.21802:127 336.18954:127 336.19989:127 336.20508:127 345.15518:127 345.17093:262 345.1814:127 348.83719:127 349.35123:257 355.07578:260 355.09439:127 355.31775:260 356.03616:257 356.05746:259 356.06808:533 356.07077:532 356.07343:801 356.07608:266 356.07874:800 356.08142:266 356.08408:535 356.08673:927 356.08939:397 356.09207:263 356.09473:531 356.09738:134 356.10004:134 356.10272:130 356.10538:263 356.11069:266 356.11337:133 356.11603:132 356.11868:130 356.12134:130 357.07513:133 358.39072:133 359.81573:262 371.12469:266 371.13284:264 371.18723:133 372.08749:130 372.09293:127 372.09839:130 372.1011:131 372.10654:261 372.10925:392 372.11197:260 372.11472:260 372.11743:130 372.12015:258 372.12286:388 372.12558:253 372.13919:130 372.14191:130 372.14465:127 372.14737:130 372.15552:256 372.1637:127 372.16641:130 372.22357:131 372.22629:357 372.23175:127 372.2399:256 372.24261:127 372.24808:256 372.25079:129 372.25351:127 372.2644:127 372.26712:129 372.2753:283 372.28345:129 374.93689:127 385.59772:127 385.85541:127 386.43484:127 386.54855:127 387.92007:253 387.93674:127 387.95065:127 388.64847:127 389.05188:285 389.05466:127 389.06577:127 389.07968:127 389.10751:343 389.13257:127 389.13815:341 389.15485:328 389.1604:231 389.16318:153 389.16599:328 389.16876:554 389.17154:1663 389.17432:1723 389.17709:1150 389.1799:2239 389.18268:5505 389.18546:4406 389.18823:4827 389.19101:6820 389.19382:3336 389.19659:6116 389.19937:3998 389.20215:3751 389.20493:2159 389.20773:1180 389.21051:699 389.21329:1376 389.21606:488 389.21884:529 389.22165:153 389.22443:367 389.22998:324 389.23276:305 389.23834:171 389.24112:215 389.2439:215 389.25504:153 389.37195:159 389.40536:231 389.51117:217 389.539:127 389.54181:127 389.57523:514 389.578:127 389.6142:153 389.64761:127 389.75626:127 390.07104:127 390.47241:171 390.4975:127 390.78751:127 390.80145:127 391.16412:127 391.18088:127 391.79501:127 391.80338:127 391.81454:127 391.93466:127 392.09668:127 392.32019:127 392.37888:127 392.44037:127 392.44595:127 392.76465:127 392.95761:127 392.9744:127 393.0415:127 393.36603:127 393.66272:127 394.04349:127 394.66269:155 395.54327:127 397.98822:127 400.77957:127 405.58136:127 406.32614:127 416.15958:127 471.19141:127 499.52637:127 | Geniposide | Fructus gardeniae |
| 7.596833 | C20H26O6 | 363.1499 | 363.1802 | [M+H]+ |  | (+)-Jurinelloide | Moschus |
| 7.607 | C19H24O6 | 349.1726 | 349.1646 | [M+H]+ | 51.2053:127 51.54508:127 115.05421:127 149.02892:127 158.09129:127 305.15341:128 305.15588:127 305.15836:258 305.1608:127 305.16327:127 305.16574:257 305.17065:258 305.1756:258 305.18051:127 316.11401:127 331.14877:127 331.15646:127 331.16159:260 331.16415:262 331.16675:394 331.16931:262 331.17188:389 331.17444:553 331.177:131 331.17957:262 331.18213:131 331.96313:131 332.0274:127 344.08148:131 348.0209:127 348.04724:127 348.05515:131 348.05777:127 348.06039:127 348.0683:333 348.0762:127 348.08673:127 348.40268:127 348.5686:127 348.8584:127 348.9928:127 349.03235:366 349.15359:354 349.1615:238 349.16415:238 349.16678:187 349.16943:427 349.17206:238 349.17468:1249 349.17734:2091 349.17996:3596 349.18262:4081 349.18524:5777 349.18787:9079 349.19052:5203 349.19315:10680 349.1958:8346 349.19843:5633 349.20105:5290 349.2037:5258 349.20633:4167 349.20895:2714 349.21161:2005 349.21423:1198 349.21689:422 349.21951:695 349.22214:337 349.22479:240 349.22742:189 349.23007:429 349.2327:626 349.23532:169 349.27753:240 349.35925:169 349.383:187 349.47003:228 349.51486:127 349.51752:211 349.52014:211 349.5228:211 349.52542:687 349.52805:337 349.5307:127 349.53333:127 349.54126:127 349.63095:127 349.64941:127 349.65469:127 349.66522:127 349.66788:127 349.6705:633 349.67316:211 349.68369:127 349.72064:127 349.80771:189 349.82617:127 349.94495:127 349.97397:127 349.98453:127 349.98718:211 349.99246:127 350.0162:127 350.05051:127 350.05579:127 350.069:127 350.1297:207 350.13498:127 350.19571:127 350.3436:127 350.35416:127 350.41489:127 350.42017:127 350.43073:127 350.54166:127 350.79794:127 350.84286:127 350.91422:127 350.93536:127 350.99088:127 351.07547:127 351.15741:127 351.18915:127 351.21558:127 351.28699:127 351.33456:127 351.40598:127 351.47211:127 351.51178:127 351.52237:127 351.52765:127 351.57526:127 351.59644:127 351.72607:127 351.7843:127 352.02249:127 352.05954:127 352.07013:127 352.24222:127 352.28989:127 352.30313:127 352.44879:127 352.86743:127 353.09271:127 353.1113:127 353.43747:127 353.72131:127 353.79562:127 354.16458:127 354.66925:127 354.79678:127 355.74615:127 356.16141:127 356.16675:127 356.34781:127 356.37979:127 357.92358:127 359.14978:127 359.60715:127 361.19009:127 361.73727:127 675.32135:127 675.80536:127 764.40558:127 770.53809:127 788.47638:127 | Gibberellin A1 | Moschus |
| 7.612 | C12H22O11 | 343.1121 | 343.1235 | [M+H]+ | 69.0342:127 87.05427:127 87.06216:127 89.05733:127 99.0462:127 101.00895:256 111.04021:127 113.57297:127 114.05462:127 114.05613:127 114.05914:129 114.06065:127 114.06216:127 114.06517:256 114.06818:253 114.06969:129 114.0712:127 114.07421:127 119.0349:127 129.06137:127 131.07426:127 131.07912:127 133.08485:127 133.09299:129 133.0995:127 135.09596:127 143.07011:127 153.03235:127 153.31871:256 158.08597:127 158.08951:256 158.09129:127 158.09306:129 158.10193:129 158.10902:127 175.09398:127 177.11415:127 179.04773:127 179.16479:127 196.12921:127 196.14305:127 197.06293:512 197.07085:256 201.45625:127 202.10759:129 202.1096:253 202.1116:129 202.1136:382 202.11761:259 202.12364:256 202.12766:129 202.12965:127 202.13968:129 202.14169:127 202.14369:127 207.08401:127 207.09215:127 221.1096:127 229.10655:127 229.37785:127 233.22295:127 239.15523:127 243.43976:127 251.11137:253 251.12032:127 251.61467:127 257.08231:127 261.14975:127 290.16254:127 290.16974:127 290.17456:127 290.17694:127 301.10013:127 301.14664:129 302.08752:127 302.15375:127 302.16354:127 302.17581:253 306.86346:127 308.79922:127 317.14551:127 325.10431:127 326.1507:127 326.16089:127 326.16342:253 326.16599:127 326.20419:127 326.43869:127 326.66559:127 326.8186:127 327.20642:127 327.99548:127 330.19659:127 343.15268:127 343.20755:127 343.22324:127 343.24155:127 411.24353:127 412.21689:127 447.19696:127 457.18436:127 475.18985:127 475.19601:127 475.26059:127 501.2016:127 501.22369:127 507.3988:127 519.22888:127 519.23853:127 519.24817:127 545.29602:127 563.25867:127 563.27539:127 563.27875:127 | Sucrose | Radix curcumae |
| 7.6775 | C16H22O9 | 359.1473 | 359.1337 | [M+2H]2+ | 67.31128:63 69.03413:63 69.04351:63 71.05678:63 73.07004:63 80.94288:63 81.0089:63 81.01271:63 83.05853:63 84.06061:63 87.04234:63 87.04629:63 88.04427:63 88.9082:63 89.05859:63 89.06657:63 99.0433:63 103.0367:63 104.04738:63 109.00974:127 115.06477:63 129.09012:63 131.0677:63 133.07986:63 133.08638:63 133.08963:63 133.09288:63 133.0994:63 133.10103:127 134.10231:63 134.10884:63 147.06155:63 148.07445:63 151.09933:63 154.05145:63 154.06197:63 156.07359:63 173.09085:63 177.10654:63 177.11592:63 177.1178:63 177.11967:63 178.12:63 184.09496:63 186.08156:63 188.11172:63 196.05995:63 199.10208:63 203.92674:63 218.09671:63 220.04292:63 229.15982:63 230.11336:63 230.13477:63 230.61873:63 231.27052:63 236.13628:63 236.14494:63 240.59071:63 241.08774:127 252.14296:63 259.11304:63 259.12439:63 259.14029:63 259.14938:63 261.14505:63 262.11951:63 262.14236:63 263.76215:63 266.94287:63 269.00302:63 281.08755:63 281.16327:63 281.16562:63 284.12344:63 284.18765:63 284.19479:63 287.15649:63 287.16846:63 288.116:63 288.16391:63 296.19928:63 298.1669:63 299.12021:63 303.13538:63 303.14029:63 303.15012:63 303.15256:63 303.15503:63 303.15747:63 303.16238:63 303.18204:63 304.16302:63 305.16776:63 305.1727:63 305.17517:63 306.17416:63 307.19211:63 313.168:63 323.25485:63 324.1687:63 324.17886:63 324.18646:63 324.21695:63 325.0126:63 325.11435:63 326.7955:63 328.21255:63 338.56552:63 339.06674:63 340.80698:63 341.02057:127 341.02579:63 341.03101:63 341.03622:127 341.03882:127 341.04141:63 341.05707:63 341.06226:63 341.10916:63 341.11697:63 342.1105:63 342.1366:63 342.16269:63 342.18097:63 342.18619:63 342.19925:63 342.20184:63 342.2149:63 342.2175:63 342.22012:63 342.73447:63 343.77222:63 344.12021:63 344.42911:63 347.17865:127 347.18127:63 347.1839:63 347.1918:63 347.19443:63 347.21021:63 348.56543:63 349.18997:63 349.19525:63 357.26135:63 359.11179:63 359.11981:63 359.13049:63 359.13852:63 359.1412:127 359.1492:63 359.15457:63 359.1626:127 359.17328:63 359.17862:63 359.18933:63 359.28024:63 359.28827:63 359.29361:63 359.2963:127 359.30698:63 359.32303:63 359.64404:63 359.86615:63 375.19873:63 375.22058:63 389.17938:63 391.19989:63 391.20267:127 391.20547:127 391.21106:63 392.23306:63 395.15842:63 411.17755:63 415.20691:63 435.2009:63 435.21561:63 435.25388:63 435.29507:63 435.31274:63 435.93701:63 445.22385:63 459.23178:63 477.22784:63 477.24634:63 477.2525:63 477.26791:63 477.271:63 477.28333:63 500.19116:63 503.25342:63 518.65015:63 521.25604:127 521.25928:63 521.26245:127 521.27216:63 521.2818:63 521.29144:127 521.29468:63 522.26154:63 522.27118:63 522.29053:63 522.32275:63 523.32281:63 541.22144:63 547.28754:127 547.29083:63 547.31726:63 548.29138:63 565.29614:63 565.30286:63 565.30957:63 565.31958:127 585.20947:63 585.24359:63 585.25726:63 591.33472:63 592.2923:63 609.30621:63 609.36194:127 609.40027:63 629.28326:63 633.62628:63 673.29028:63 695.28217:63 695.28961:63 695.3045:63 695.35284:63 696.26465:63 | Gardaloside | Fructus gardeniae |
| 7.753334 | C20H26O7 | 379.1804 | 379.1751 | [M+2H]2+ |  | Gibberellin A17 | Moschus |
| 8.05865 | C19H24O5 | 333.1977 | 333.1697 | [M+2H]2+ |  | Gibberellin A4 | Moschus |
| 8.121984 | C20H30O4 | 335.2092 | 335.2217 | [M+H]+ | 111.01083:259 113.04619:127 123.06481:258 133.07709:132 133.07872:132 133.08034:262 133.08197:131 133.0836:653 133.08522:264 133.09987:127 133.1015:127 133.10475:131 137.06308:127 145.5824:127 151.0981:127 165.05493:127 177.10893:127 177.11082:127 177.1127:132 177.13147:127 178.11676:127 186.09558:127 199.12254:127 221.13733:127 221.15831:127 230.1118:127 240.15384:127 252.13686:127 261.12973:127 305.15854:127 318.19461:127 318.21725:127 318.68808:127 331.17459:127 331.18744:127 333.16226:127 335.15588:127 349.18536:127 349.78937:127 371.18201:127 373.16348:127 375.19394:127 375.20486:127 375.22128:127 393.22073:127 397.25693:257 412.22888:127 415.24213:127 433.20541:127 433.20837:127 433.2113:258 459.29901:256 477.21933:131 477.22549:131 477.22858:131 477.24091:129 477.24399:129 477.26556:129 478.25238:129 487.57834:127 501.29691:127 503.33011:256 519.23907:256 520.36169:131 521.24066:130 521.24713:130 521.25037:130 521.25677:130 521.26324:129 521.26648:259 521.26965:129 521.27936:129 521.28577:130 521.289:382 522.16553:256 547.32465:127 565.27679:256 565.28687:259 565.29352:127 565.29688:129 565.30023:258 565.32373:129 565.72974:127 653.32843:129 653.33563:129 653.33923:130 653.34283:384 653.34644:129 653.35004:130 653.35724:130 653.36084:259 653.36444:131 653.3681:129 653.39691:131 | Zerumin B | Radix curcumae |
| 8.158816 | C21H20O6 | 369.1054 | 369.1333 | [M+H]+ |  | Curcumin | Radix curcumae |
| 8.402133 | C26H40O10 | 513.3009 | 513.2695 | [M+H]+ |  | Curcumanggoside | Radix curcumae |
| 8.508966 | C10H12N2 | 161.1265 | 161.1074 | [M+H]+ |  | Tryptamine | Radix curcumae |
| 8.998266 | C10H16 | 137.1048 | 137.1325 | [M+H]+ | 121.06314:127 137.09418:130 137.10078:127 137.10243:380 137.1041:130 137.10574:130 137.10904:389 137.1107:129 138.47867:130 | alpha-Pinene | Radix curcumae |
| 9.806566 | C15H22O2 | 235.1615 | 235.1693 | [M+H]+ |  | 13-Hydroxygermacrone | Radix curcumae |
| 9.885067 | C15H24O2 | 237.1778 | 237.1849 | [M+H]+ |  | Curcumalactone | Radix curcumae |
| 2.522367 | C2H4O2 | 59.01869 | 59.01383 | [M-H]- |  | Acetic acid | Radix curcumae |
| 4.677484 | C15H24O | 219.1854 | 219.1754 | [M-H]- |  | Spathulenol | Radix curcumae |
| 6.35935 | C15H26O | 221.1599 | 221.1911 | [M-H]- |  | trans,trans-Farnesol | Moschus |
| 8.402583 | C32H50O4 | 497.3567 | 497.3636 | [M-H]- | 62.0003:127 129.12621:257 225.18922:256 433.37149:510 435.10629:256 451.3371:129 451.3551:259 451.35809:130 451.36108:256 451.3671:130 451.37009:776 451.37308:130 451.37607:129 451.37909:258 451.38208:129 451.38507:389 451.39108:256 451.39407:259 451.39706:257 451.40005:389 451.40607:127 451.40906:129 451.41806:130 451.42105:130 451.42703:129 457.2468:129 489.38315:259 494.64758:258 495.79056:127 497.0481:256 497.37216:130 497.37531:127 497.38477:130 497.38791:259 497.39105:130 497.40048:130 497.40677:388 497.40994:256 497.41623:387 497.41937:130 497.42252:129 497.42566:257 497.43195:256 497.43512:127 497.52637:129 497.96396:127 498.22849:127 | Acetylursolic acid | Fructus gardeniae |
| 9.10755 | C7H6O2 | 121.0371 | 121.0295 | [M-H]- |  | 4-Hydroxybenzaldehyde | Radix curcumae |
| 9.92225 | C15H24O3 | 251.139 | 251.1652 | [M-H]- |  | Zedoarondiol | Radix curcumae |
