# Supplementary material for: Uncovering the Mechanism of the Xingnaojing Injection against Ischemic Stroke Using a Combined Network Pharmacology Approach and Gut Microbiota Analysis
Source: Evid Based Complement Alternat Med. 2022 May 20;2022:5886698. doi: 10.1155/2022/5886698 (PMC9142292; doi:10.1155/2022/5886698)
Supplement: Supplementary Materials — Supplementary Table S1: active ingredients of XNJ identified by UHPLC-MS. Supplementary Table S2: 507 compound-related targets. Supplementary Table S3: 1667 IS-related targets and 2074 IBD-related targets. Supplementary Table S4: 210 shared targets were identified as potential therapeutic targets of XNJ against IS. . [file 5886698.f1.zip › 5886698.f1/S2 compound names-targets.docx]

| compound names | targets |
| --- | --- |
| Gibberellin A1 | HSD11B1 |
| Gibberellin A1 | PTGS2 |
| Gibberellin A1 | LTB4R |
| Gibberellin A1 | IDO1 |
| Gibberellin A1 | HMGCR |
| Gibberellin A1 | AMPD2 |
| Gibberellin A1 | ACE |
| Gibberellin A1 | ABCB1 |
| Gibberellin A1 | NR3C1 |
| Gibberellin A1 | GSR |
| Gibberellin A1 | PREP |
| Gibberellin A1 | PRSS1 |
| Gibberellin A1 | CTRC |
| Gibberellin A1 | IMPDH1 |
| Gibberellin A1 | AKR1B1 |
| Gibberellin A1 | SLC22A12 |
| Gibberellin A1 | CPA1 |
| Gibberellin A1 | PTGS1 |
| Gibberellin A1 | MMP3 |
| Gibberellin A1 | PARP1 |
| Gibberellin A1 | LANCL2 |
| Gibberellin A1 | SLC2A1 |
| Gibberellin A1 | ADCY1 |
| Gibberellin A1 | LTA4H |
| Gibberellin A1 | AMPD3 |
| Gibberellin A1 | MME |
| Gibberellin A1 | NR3C2 |
| Gibberellin A1 | IMPDH2 |
| Gibberellin A1 | CTSA |
| Gibberellin A1 | SLC6A4 |
| Gibberellin A1 | PRKCA |
| Gibberellin A1 | ITGB1 |
| Gibberellin A1 | ITGA4 |
| Gibberellin A1 | ITGAL |
| Gibberellin A1 | ICAM1 |
| Gibberellin A1 | ITGB2 |
| Gibberellin A1 | PPP1CC |
| Gibberellin A1 | CTNNB1 |
| Gibberellin A1 | CSNK2A1 |
| Gibberellin A1 | ECE1 |
| Gibberellin A1 | EGFR |
| Gibberellin A1 | PIM1 |
| Gibberellin A1 | SRC |
| Gibberellin A1 | PIM2 |
| Gibberellin A1 | ACLY |
| Gibberellin A1 | MAPK8 |
| Gibberellin A1 | EDNRB |
| Gibberellin A1 | PYGL |
| Gibberellin A1 | PYGM |
| Gibberellin A1 | ANPEP |
| Gibberellin A1 | IGF1R |
| Gibberellin A1 | TEK |
| Gibberellin A1 | PTGES |
| Gibberellin A1 | TTR |
| Gibberellin A1 | PTGDR2 |
| Gibberellin A1 | HSD11B2 |
| Gibberellin A1 | CREBBP |
| Gibberellin A1 | WEE1 |
| Gibberellin A4 | HSD11B1 |
| Gibberellin A4 | CPA1 |
| Gibberellin A4 | PTGS2 |
| Gibberellin A4 | AMPD2 |
| Gibberellin A4 | PREP |
| Gibberellin A4 | PRSS1 |
| Gibberellin A4 | LTB4R |
| Gibberellin A4 | CTRC |
| Gibberellin A4 | PTGES |
| Gibberellin A4 | IDO1 |
| Gibberellin A4 | MAPK8 |
| Gibberellin A4 | AMPD3 |
| Gibberellin A4 | NR3C1 |
| Gibberellin A4 | PTGDR2 |
| Gibberellin A4 | NR3C2 |
| Gibberellin A4 | HMGCR |
| Gibberellin A4 | PRKCA |
| Gibberellin A4 | AMPD1 |
| Gibberellin A4 | ITGAL |
| Gibberellin A4 | ICAM1 |
| Gibberellin A4 | ITGB2 |
| Gibberellin A4 | OPRM1 |
| Gibberellin A4 | PSEN2 |
| Gibberellin A4 | PSENEN |
| Gibberellin A4 | NCSTN |
| Gibberellin A4 | APH1A |
| Gibberellin A4 | PSEN1 |
| Gibberellin A4 | APH1B |
| Gibberellin A4 | PPP1CC |
| Gibberellin A4 | MME |
| Gibberellin A4 | EDNRA |
| Gibberellin A4 | POLB |
| Gibberellin A4 | LTA4H |
| Gibberellin A4 | UGT2B7 |
| Gibberellin A4 | CTSA |
| Gibberellin A4 | POLA1 |
| Gibberellin A4 | SLC2A1 |
| Gibberellin A4 | ADCY1 |
| Gibberellin A4 | ECE1 |
| Gibberellin A4 | FLT1 |
| Gibberellin A4 | KDR |
| Gibberellin A4 | ITGB1 |
| Gibberellin A4 | ITGA4 |
| Gibberellin A4 | AKR1B1 |
| Gibberellin A4 | EPHX2 |
| Gibberellin A4 | ROCK2 |
| Gibberellin A4 | PYGL |
| Gibberellin A4 | MMP3 |
| Gibberellin A4 | PYGM |
| Gibberellin A4 | AR |
| Gibberellin A4 | GSR |
| Gibberellin A4 | ACE2 |
| Gibberellin A4 | CCR9 |
| Gibberellin A4 | PARP1 |
| Gibberellin A4 | PTGER4 |
| Gibberellin A8 | PTGS2 |
| Gibberellin A8 | AMPD3 |
| Gibberellin A8 | AMPD2 |
| Gibberellin A8 | SLC2A1 |
| Gibberellin A8 | ADCY1 |
| Gibberellin A8 | PREP |
| Gibberellin A8 | ABCB1 |
| Gibberellin A8 | HMGCR |
| Gibberellin A8 | AR |
| Gibberellin A8 | NR3C2 |
| Gibberellin A8 | SELP |
| Gibberellin A8 | SLC22A12 |
| Gibberellin A8 | IMPDH2 |
| Gibberellin A8 | GSR |
| Gibberellin A8 | PRKCA |
| Gibberellin A8 | PRSS1 |
| Gibberellin A8 | MME |
| Gibberellin A8 | HSD11B1 |
| Gibberellin A8 | CTRC |
| Gibberellin A8 | CTSA |
| Gibberellin A8 | CSNK2A1 |
| Gibberellin A8 | ACE |
| Gibberellin A8 | PPP5C |
| Gibberellin A8 | PPP1CA |
| Gibberellin A8 | PPP1CC |
| Gibberellin A8 | CES2 |
| Gibberellin A8 | FBP1 |
| Gibberellin A8 | IDO1 |
| Gibberellin A8 | LTA4H |
| Gibberellin A8 | TERT |
| Gibberellin A8 | IMPDH1 |
| Gibberellin A8 | F2 |
| Gibberellin A8 | LTB4R |
| Gibberellin A8 | TRPV4 |
| Gibberellin A8 | SELL |
| Gibberellin A8 | SELE |
| Gibberellin A8 | NR3C1 |
| Gibberellin A8 | WEE1 |
| Gibberellin A8 | ANPEP |
| Gibberellin A8 | ECE1 |
| Gibberellin A8 | POLB |
| Gibberellin A8 | GLRA2 |
| Gibberellin A8 | ITGB1 |
| Gibberellin A8 | ITGA4 |
| Gibberellin A17 | HSD11B1 |
| Gibberellin A17 | SAE1 |
| Gibberellin A17 | UBA2 |
| Gibberellin A17 | AKR1B10 |
| Gibberellin A17 | NR1H4 |
| Gibberellin A17 | PTPN1 |
| Gibberellin A17 | POLB |
| Gibberellin A17 | PTGES |
| Gibberellin A17 | TOP2A |
| Gibberellin A17 | CDC25C |
| Gibberellin A17 | CDC25A |
| Gibberellin A17 | MME |
| Gibberellin A17 | AR |
| Gibberellin A17 | CES2 |
| Gibberellin A17 | FNTA |
| Gibberellin A17 | FNTB |
| Hyperin | AKR1B1 |
| Hyperin | CA2 |
| Hyperin | CA7 |
| Hyperin | CA12 |
| Hyperin | CA4 |
| Hyperin | NOX4 |
| Hyperin | ADRA2C |
| Hyperin | ACHE |
| Hyperin | NQO2 |
| Hyperin | RPS6KA3 |
| Hyperin | NMUR2 |
| Hyperin | ADRA2A |
| Hyperin | PTGS2 |
| Hyperin | CD38 |
| Hyperin | PDE5A |
| Hyperin | TNF |
| Hyperin | IL2 |
| Hyperin | ADORA1 |
| Hyperin | XDH |
| Hyperin | ALOX5 |
| Hyperin | SLC29A1 |
| Hyperin | TERT |
| trans,trans-Farnesol | SQLE |
| trans,trans-Farnesol | CNR1 |
| trans,trans-Farnesol | PGR |
| trans,trans-Farnesol | CNR2 |
| trans,trans-Farnesol | PRKCA |
| trans,trans-Farnesol | NR1H2 |
| trans,trans-Farnesol | TBXAS1 |
| trans,trans-Farnesol | JAK2 |
| trans,trans-Farnesol | HSD11B1 |
| trans,trans-Farnesol | CES2 |
| trans,trans-Farnesol | AKR1C3 |
| trans,trans-Farnesol | F2R |
| trans,trans-Farnesol | PIM1 |
| trans,trans-Farnesol | PIM3 |
| trans,trans-Farnesol | RXRA |
| trans,trans-Farnesol | G6PD |
| trans,trans-Farnesol | HSD11B2 |
| trans,trans-Farnesol | CCND1 |
| trans,trans-Farnesol | CDK4 |
| trans,trans-Farnesol | GSK3B |
| trans,trans-Farnesol | MGLL |
| 4-Hydroxybenzaldehyde | ERN1 |
| 4-Hydroxybenzaldehyde | CA2 |
| 4-Hydroxybenzaldehyde | CA7 |
| 4-Hydroxybenzaldehyde | CA1 |
| 4-Hydroxybenzaldehyde | CA3 |
| 4-Hydroxybenzaldehyde | CA6 |
| 4-Hydroxybenzaldehyde | CA12 |
| 4-Hydroxybenzaldehyde | CA14 |
| 4-Hydroxybenzaldehyde | CA9 |
| 4-Hydroxybenzaldehyde | CA4 |
| 4-Hydroxybenzaldehyde | CA5B |
| 4-Hydroxybenzaldehyde | CA5A |
| 4-Hydroxybenzaldehyde | CA13 |
| 4-Hydroxybenzaldehyde | COMT |
| 4-Hydroxybenzaldehyde | ACHE |
| 4-Hydroxybenzaldehyde | ALDH5A1 |
| 4-Hydroxybenzaldehyde | TYR |
| 4-Hydroxybenzaldehyde | ABAT |
| 4-Hydroxybenzaldehyde | FUT7 |
| 4-Hydroxybenzoic acid | CA2 |
| 4-Hydroxybenzoic acid | CA7 |
| 4-Hydroxybenzoic acid | CA1 |
| 4-Hydroxybenzoic acid | CA3 |
| 4-Hydroxybenzoic acid | CA6 |
| 4-Hydroxybenzoic acid | CA12 |
| 4-Hydroxybenzoic acid | CA14 |
| 4-Hydroxybenzoic acid | CA9 |
| 4-Hydroxybenzoic acid | CA4 |
| 4-Hydroxybenzoic acid | CA5B |
| 4-Hydroxybenzoic acid | CA5A |
| 4-Hydroxybenzoic acid | CA13 |
| 4-Hydroxybenzoic acid | FUT7 |
| 4-Hydroxybenzoic acid | DAO |
| 4-Hydroxybenzoic acid | SRD5A2 |
| 4-Hydroxybenzoic acid | ESR2 |
| 4-Hydroxybenzoic acid | SQLE |
| 4-Hydroxybenzoic acid | AKR1C2 |
| 4-Hydroxybenzoic acid | AKR1C1 |
| 4-Hydroxybenzoic acid | ERN1 |
| 4-Hydroxybenzoic acid | HDAC6 |
| 4-Hydroxybenzoic acid | HDAC8 |
| 4-Hydroxybenzoic acid | LDHA |
| 4-Hydroxybenzoic acid | LDHB |
| 6-Methyl-7-(3-oxobutyl)-bicyclo[4.1.0]heptan-3-one | CYP19A1 |
| 6-Methyl-7-(3-oxobutyl)-bicyclo[4.1.0]heptan-3-one | CA2 |
| 6-Methyl-7-(3-oxobutyl)-bicyclo[4.1.0]heptan-3-one | CA1 |
| 6-Methyl-7-(3-oxobutyl)-bicyclo[4.1.0]heptan-3-one | CA4 |
| 6-Methyl-7-(3-oxobutyl)-bicyclo[4.1.0]heptan-3-one | NR1I3 |
| 6-Methyl-7-(3-oxobutyl)-bicyclo[4.1.0]heptan-3-one | CES1 |
| 6-Methyl-7-(3-oxobutyl)-bicyclo[4.1.0]heptan-3-one | CES2 |
| 6-Methyl-7-(3-oxobutyl)-bicyclo[4.1.0]heptan-3-one | AR |
| 6-Methyl-7-(3-oxobutyl)-bicyclo[4.1.0]heptan-3-one | PARP1 |
| 6-Methyl-7-(3-oxobutyl)-bicyclo[4.1.0]heptan-3-one | PTGS2 |
| 6-Methyl-7-(3-oxobutyl)-bicyclo[4.1.0]heptan-3-one | NOS2 |
| 6-Methyl-7-(3-oxobutyl)-bicyclo[4.1.0]heptan-3-one | SLC6A2 |
| 6-Methyl-7-(3-oxobutyl)-bicyclo[4.1.0]heptan-3-one | SLC6A4 |
| 6-Methyl-7-(3-oxobutyl)-bicyclo[4.1.0]heptan-3-one | SLC6A3 |
| 6-Methyl-7-(3-oxobutyl)-bicyclo[4.1.0]heptan-3-one | CHRM1 |
| 6-Methyl-7-(3-oxobutyl)-bicyclo[4.1.0]heptan-3-one | MTNR1A |
| 6-Methyl-7-(3-oxobutyl)-bicyclo[4.1.0]heptan-3-one | MTNR1B |
| 6-Methyl-7-(3-oxobutyl)-bicyclo[4.1.0]heptan-3-one | P2RX7 |
| 6-Methyl-7-(3-oxobutyl)-bicyclo[4.1.0]heptan-3-one | EPHX2 |
| 6-Methyl-7-(3-oxobutyl)-bicyclo[4.1.0]heptan-3-one | PARP2 |
| 6-Methyl-7-(3-oxobutyl)-bicyclo[4.1.0]heptan-3-one | SRD5A1 |
| 6-Methyl-7-(3-oxobutyl)-bicyclo[4.1.0]heptan-3-one | BRD4 |
| 6-Methyl-7-(3-oxobutyl)-bicyclo[4.1.0]heptan-3-one | CAPN1 |
| 6-Methyl-7-(3-oxobutyl)-bicyclo[4.1.0]heptan-3-one | ESR2 |
| 6-Methyl-7-(3-oxobutyl)-bicyclo[4.1.0]heptan-3-one | ESR1 |
| 6-Methyl-7-(3-oxobutyl)-bicyclo[4.1.0]heptan-3-one | SRD5A2 |
| 6-Methyl-7-(3-oxobutyl)-bicyclo[4.1.0]heptan-3-one | MAOA |
| 6-Methyl-7-(3-oxobutyl)-bicyclo[4.1.0]heptan-3-one | MAOB |
| 6-Methyl-7-(3-oxobutyl)-bicyclo[4.1.0]heptan-3-one | SIGMAR1 |
| 6-Methyl-7-(3-oxobutyl)-bicyclo[4.1.0]heptan-3-one | CTSK |
| 6-Methyl-7-(3-oxobutyl)-bicyclo[4.1.0]heptan-3-one | DPP4 |
| 6-Methyl-7-(3-oxobutyl)-bicyclo[4.1.0]heptan-3-one | HDAC6 |
| 6-Methyl-7-(3-oxobutyl)-bicyclo[4.1.0]heptan-3-one | CTSS |
| 6-Methyl-7-(3-oxobutyl)-bicyclo[4.1.0]heptan-3-one | CYP1A2 |
| 6-Methyl-7-(3-oxobutyl)-bicyclo[4.1.0]heptan-3-one | NQO2 |
| 6-Methyl-7-(3-oxobutyl)-bicyclo[4.1.0]heptan-3-one | CTSB |
| 6-Methyl-7-(3-oxobutyl)-bicyclo[4.1.0]heptan-3-one | CYP11B1 |
| 6-Methyl-7-(3-oxobutyl)-bicyclo[4.1.0]heptan-3-one | CYP11B2 |
| 6-Methyl-7-(3-oxobutyl)-bicyclo[4.1.0]heptan-3-one | ACE |
| 6-Methyl-7-(3-oxobutyl)-bicyclo[4.1.0]heptan-3-one | MALT1 |
| 6-Methyl-7-(3-oxobutyl)-bicyclo[4.1.0]heptan-3-one | SYK |
| 6-Methyl-7-(3-oxobutyl)-bicyclo[4.1.0]heptan-3-one | ABCG2 |
| 6-Methyl-7-(3-oxobutyl)-bicyclo[4.1.0]heptan-3-one | DHODH |
| 6-Methyl-7-(3-oxobutyl)-bicyclo[4.1.0]heptan-3-one | HTR2C |
| 6-Methyl-7-(3-oxobutyl)-bicyclo[4.1.0]heptan-3-one | OPRM1 |
| 6-Methyl-7-(3-oxobutyl)-bicyclo[4.1.0]heptan-3-one | OPRD1 |
| 6-Methyl-7-(3-oxobutyl)-bicyclo[4.1.0]heptan-3-one | F2 |
| 6-Methyl-7-(3-oxobutyl)-bicyclo[4.1.0]heptan-3-one | PRSS1 |
| 6-Methyl-7-(3-oxobutyl)-bicyclo[4.1.0]heptan-3-one | CHRM2 |
| 6-Methyl-7-(3-oxobutyl)-bicyclo[4.1.0]heptan-3-one | PTGS1 |
| 6-Methyl-7-(3-oxobutyl)-bicyclo[4.1.0]heptan-3-one | KDR |
| 6-Methyl-7-(3-oxobutyl)-bicyclo[4.1.0]heptan-3-one | GABRB3 |
| 6-Methyl-7-(3-oxobutyl)-bicyclo[4.1.0]heptan-3-one | GABRG2 |
| 6-Methyl-7-(3-oxobutyl)-bicyclo[4.1.0]heptan-3-one | GABRA5 |
| 6-Methyl-7-(3-oxobutyl)-bicyclo[4.1.0]heptan-3-one | GABRA1 |
| 6-Methyl-7-(3-oxobutyl)-bicyclo[4.1.0]heptan-3-one | GABRB2 |
| 6-Methyl-7-(3-oxobutyl)-bicyclo[4.1.0]heptan-3-one | GABRA4 |
| 6-Methyl-7-(3-oxobutyl)-bicyclo[4.1.0]heptan-3-one | CTSC |
| 6-Methyl-7-(3-oxobutyl)-bicyclo[4.1.0]heptan-3-one | GSK3B |
| 6-Methyl-7-(3-oxobutyl)-bicyclo[4.1.0]heptan-3-one | GPR139 |
| 6-Methyl-7-(3-oxobutyl)-bicyclo[4.1.0]heptan-3-one | TRPV1 |
| 6-Methyl-7-(3-oxobutyl)-bicyclo[4.1.0]heptan-3-one | PREP |
| 6-Methyl-7-(3-oxobutyl)-bicyclo[4.1.0]heptan-3-one | FAP |
| 6-Methyl-7-(3-oxobutyl)-bicyclo[4.1.0]heptan-3-one | HMOX1 |
| 6-Methyl-7-(3-oxobutyl)-bicyclo[4.1.0]heptan-3-one | NAAA |
| 13-Hydroxygermacrone | PRKCG |
| 13-Hydroxygermacrone | PRKCD |
| 13-Hydroxygermacrone | PSEN2 |
| 13-Hydroxygermacrone | PSENEN |
| 13-Hydroxygermacrone | NCSTN |
| 13-Hydroxygermacrone | APH1A |
| 13-Hydroxygermacrone | PSEN1 |
| 13-Hydroxygermacrone | APH1B |
| 13-Hydroxygermacrone | PRKCB |
| 13-Hydroxygermacrone | PRKCE |
| 13-Hydroxygermacrone | PRKCQ |
| 13-Hydroxygermacrone | JAK3 |
| 13-Hydroxygermacrone | JAK1 |
| 13-Hydroxygermacrone | JAK2 |
| 13-Hydroxygermacrone | TYK2 |
| 13-Hydroxygermacrone | HSD17B2 |
| 13-Hydroxygermacrone | AKR1C3 |
| 13-Hydroxygermacrone | RASGRP1 |
| 13-Hydroxygermacrone | EPHX1 |
| 13-Hydroxygermacrone | IL6ST |
| 13-Hydroxygermacrone | TYMS |
| 13-Hydroxygermacrone | KCNH2 |
| 13-Hydroxygermacrone | LTA4H |
| 13-Hydroxygermacrone | HMOX1 |
| 13-Hydroxygermacrone | ABCC9 |
| 13-Hydroxygermacrone | PER2 |
| 13-Hydroxygermacrone | DRD2 |
| 13-Hydroxygermacrone | PIM1 |
| 13-Hydroxygermacrone | PIM3 |
| 13-Hydroxygermacrone | CHRM4 |
| 13-Hydroxygermacrone | CHRM5 |
| 13-Hydroxygermacrone | CHRM2 |
| 13-Hydroxygermacrone | CHRM1 |
| 13-Hydroxygermacrone | CHRM3 |
| 13-Hydroxygermacrone | CYP2C9 |
| 13-Hydroxygermacrone | EDNRA |
| 13-Hydroxygermacrone | CCNB3 |
| 13-Hydroxygermacrone | CDK1 |
| 13-Hydroxygermacrone | CCNB1 |
| 13-Hydroxygermacrone | CCNB2 |
| 13-Hydroxygermacrone | GSK3B |
| 13-Hydroxygermacrone | TRPV3 |
| 13-Hydroxygermacrone | CHRNA3 |
| 13-Hydroxygermacrone | CHRNB4 |
| 13-Hydroxygermacrone | NPY5R |
| 13-Hydroxygermacrone | VDR |
| 13-Hydroxygermacrone | EGFR |
| 13-Hydroxygermacrone | PYGL |
| 13-Hydroxygermacrone | CYP19A1 |
| Acetic acid | PTPN1 |
| Acetic acid | GSTP1 |
| alpha-Pinene | PPARA |
| alpha-Pinene | CNR2 |
| alpha-Pinene | ACHE |
| alpha-Pinene | FAAH |
| alpha-Pinene | TRPV1 |
| alpha-Pinene | AR |
| alpha-Pinene | CYP19A1 |
| alpha-Pinene | ESR1 |
| alpha-Pinene | CHRM2 |
| alpha-Pinene | SLC6A2 |
| alpha-Pinene | SLC6A4 |
| alpha-Pinene | CYP2C19 |
| alpha-Pinene | BCHE |
| alpha-Pinene | PTPN1 |
| Bis(4-Hydroxybenzyl) ether | ESR2 |
| Bis(4-Hydroxybenzyl) ether | ESR1 |
| Bis(4-Hydroxybenzyl) ether | CA2 |
| Bis(4-Hydroxybenzyl) ether | CA1 |
| Bis(4-Hydroxybenzyl) ether | CA9 |
| Bis(4-Hydroxybenzyl) ether | CA14 |
| Bis(4-Hydroxybenzyl) ether | CA7 |
| Bis(4-Hydroxybenzyl) ether | CA12 |
| Bis(4-Hydroxybenzyl) ether | DYRK1A |
| Bis(4-Hydroxybenzyl) ether | CLK1 |
| Bis(4-Hydroxybenzyl) ether | DYRK1B |
| Bis(4-Hydroxybenzyl) ether | SLC22A12 |
| Bis(4-Hydroxybenzyl) ether | ADORA1 |
| Bis(4-Hydroxybenzyl) ether | ADORA2A |
| Bis(4-Hydroxybenzyl) ether | ADORA2B |
| Bis(4-Hydroxybenzyl) ether | MCL1 |
| Curcumalactone | F2 |
| Curcumalactone | PRSS1 |
| Curcumalactone | PTGS2 |
| Curcumalactone | CTRC |
| Curcumalactone | HSD11B1 |
| Curcumalactone | CYP19A1 |
| Curcumalactone | CYP17A1 |
| Curcumalactone | PREP |
| Curcumalactone | CTSK |
| Curcumalactone | CTSL |
| Curcumalactone | CTSB |
| Curcumalactone | CTSH |
| Curcumalactone | PTPN1 |
| Curcumalactone | EPHX1 |
| Curcumalactone | ADRA2A |
| Curcumalactone | ADRA2C |
| Curcumalactone | ADRA2B |
| Curcumalactone | SLC6A4 |
| Curcumalactone | POLB |
| Curcumalactone | TAAR1 |
| Curcumalactone | HTR1A |
| Curcumalactone | SLC6A3 |
| Curcumalactone | PLA2G6 |
| Curcumalactone | ALDH2 |
| Curcumalactone | ADRA1A |
| Curcumalactone | AR |
| Curcumalactone | PGR |
| Curcumalactone | KCNH2 |
| Curcumalactone | NOS2 |
| Curcumalactone | QPCT |
| Curcumalactone | FAAH |
| Curcumalactone | BCHE |
| Curcumalactone | LIPE |
| Curcumalactone | ABCC1 |
| Curcumalactone | TOP2A |
| Curcumalactone | PDE10A |
| Curcumalactone | GRM5 |
| Curcumalactone | PTGES |
| Curcumalactone | CCR4 |
| Curcumalactone | KCNMA1 |
| Curcumalactone | CYP11B1 |
| Curcumalactone | PIK3CD |
| Curcumalactone | PIK3R1 |
| Curcumalactone | CYP11B2 |
| Curcumalactone | P2RX7 |
| Curcumalactone | NOS1 |
| Curcumalactone | NOS3 |
| Curcumalactone | NR1I2 |
| Curcumalactone | IDH1 |
| Curcumalactone | HRH3 |
| Curcumalactone | HRH4 |
| Curcumalactone | GABRA2 |
| Curcumalactone | GABRB2 |
| Curcumalactone | GABRG2 |
| Curcumalactone | CHRNA4 |
| Curcumalactone | GSK3B |
| Curcumalactone | TRPA1 |
| Curcumalactone | TGFBR1 |
| Curcumanggoside | GLRA1 |
| Curcumanggoside | GLRA2 |
| Curcumanggoside | STAT3 |
| Curcumanggoside | RORC |
| Curcumanggoside | ATP1A1 |
| Curcumanggoside | OPRM1 |
| Curcumanggoside | SLC5A2 |
| Curcumanggoside | SLC5A1 |
| Curcumanggoside | PRKCE |
| Curcumanggoside | PRKCH |
| Curcumanggoside | PRKCQ |
| Curcumanggoside | OPRK1 |
| Curcumanggoside | TYMS |
| Curcumanggoside | PPM1B |
| Curcumanggoside | PPP2R5A |
| Curcumanggoside | PTPN1 |
| Curcumanggoside | AMPD3 |
| Curcumanggoside | RPS6KA5 |
| Curcumanggoside | PTPA |
| Curcumanggoside | SELL |
| Curcumanggoside | SELP |
| Curcumanggoside | NTRK1 |
| Curcumenolactone C | PLA2G1B |
| Curcumenolactone C | CYP19A1 |
| Curcumenolactone C | RPS6KA5 |
| Curcumenolactone C | PGR |
| Curcumenolactone C | AR |
| Curcumenolactone C | ATP12A |
| Curcumenolactone C | PTPN1 |
| Curcumenolactone C | SLC6A2 |
| Curcumenolactone C | PARP1 |
| Curcumenolactone C | CDC25A |
| Curcumenolactone C | MDM2 |
| Curcumenolactone C | SIGMAR1 |
| Curcumenolactone C | CDC25B |
| Curcumenolactone C | ADRA2C |
| Curcumenolactone C | OPRM1 |
| Curcumenolactone C | OPRK1 |
| Curcumenolactone C | CDC25C |
| Curcumenolactone C | TNNC1 |
| Curcumenolactone C | TNNT2 |
| Curcumenolactone C | TNNI3 |
| Curcumenolactone C | DRD1 |
| Curcumenolactone C | KCNA5 |
| Curcumenolactone C | CHRM4 |
| Curcumenolactone C | CHRM2 |
| Curcumenolactone C | JAK1 |
| Curcumenolactone C | PPARD |
| Curcumenolactone C | JAK2 |
| Curcumenolactone C | BRD4 |
| Curcumenolactone C | PIM1 |
| Curcumenolactone C | PIM3 |
| Curcumenolactone C | MAPK8 |
| Curcumenolactone C | MAPK10 |
| Curcumenolactone C | JAK3 |
| Curcumenolactone C | ADORA2A |
| Curcumenolactone C | TYK2 |
| Curcumenolactone C | NR3C2 |
| Curcumenolactone C | DCTPP1 |
| Curcumenolactone C | AURKA |
| Curcumenolactone C | PLA2G2A |
| Curcumenolactone C | CYP51A1 |
| Curcumenolactone C | PDE10A |
| Curcumenolactone C | KIF11 |
| Curcumenolactone C | P2RX7 |
| Curcumenolactone C | TRPV3 |
| Curcumenolactone C | TTK |
| Curcumenolactone C | LRRK2 |
| Curcumenolactone C | STK3 |
| Curcumenolactone C | STK26 |
| Curcumenolactone C | CYP11B1 |
| Curcumenolactone C | CYP11B2 |
| Curcumenolactone C | HMOX1 |
| Curcumenolactone C | PTGS2 |
| Curcumenolactone C | PIK3CA |
| Curcumenolactone C | CHRM5 |
| Curcumenolactone C | DRD4 |
| Curcumenolactone C | DRD3 |
| Curcumenolactone C | CHRM3 |
| Curcumenolactone C | MAP3K14 |
| Curcumenolactone C | CDK5R1 |
| Curcumenolactone C | CDK5 |
| Curcumenolactone C | PSEN2 |
| Curcumenolactone C | PSENEN |
| Curcumenolactone C | NCSTN |
| Curcumenolactone C | APH1A |
| Curcumenolactone C | PSEN1 |
| Curcumenolactone C | APH1B |
| Curcumenolactone C | MAPK14 |
| Curcumin | MAOA |
| Curcumin | APP |
| Curcumin | EP300 |
| Curcumin | PTGES |
| Curcumin | TLR9 |
| Curcumin | BACE1 |
| Curcumin | TOP2A |
| Curcumin | NFE2L2 |
| Curcumin | ALOX5 |
| Curcumin | PTGS1 |
| Curcumin | GLO1 |
| Curcumin | IKBKG |
| Curcumin | IKBKB |
| Curcumin | CHUK |
| Curcumin | EGFR |
| Curcumin | HSD17B3 |
| Curcumin | AKT1 |
| Curcumin | STAT3 |
| Curcumin | HSD11B1 |
| Curcumin | CA7 |
| Curcumin | CA6 |
| Curcumin | CA12 |
| Curcumin | CA14 |
| Curcumin | CA9 |
| Curcumin | CA5A |
| Curcumin | GSK3B |
| Curcumin | ABCC1 |
| Curcumin | RAF1 |
| Curcumin | BRAF |
| Curcumin | CHEK1 |
| Curcumin | WEE1 |
| Curcumin | PDK1 |
| Curcumin | CA2 |
| Curcumin | CA1 |
| Curcumin | TOP1 |
| Curcumin | GCGR |
| Curcumin | AURKB |
| Curcumin | MMP13 |
| Curcumin | ADAM17 |
| Curcumin | RPS6KB1 |
| Curcumin | AURKA |
| Curcumin | TYR |
| Curcumin | ALOX5AP |
| Curcumin | SERPINE1 |
| Curcumin | CDK2 |
| Curcumin | CCNA1 |
| Curcumin | CCNA2 |
| Curcumin | MMP14 |
| Curcumin | NOX4 |
| Curcumin | CFD |
| Curcumin | AGTR1 |
| Curcumin | BMP1 |
| Curcumin | MELK |
| Curcumin | ALPL |
| Curcumin | GRIK1 |
| Curcumin | THRA |
| Curcumin | THRB |
| Curcumin | MMP8 |
| Curcumin | ADAM10 |
| Curcumin | IMPDH2 |
| Curcumin | CXCR2 |
| Curcumin | CELA1 |
| Curcumin | PREP |
| Curcumin | SGK1 |
| Curcumin | DPP4 |
| Curcumin | DPP7 |
| Curcumin | MMP9 |
| Curcumin | IMPDH1 |
| Curcumin | MMP3 |
| Curcumin | MMP7 |
| Curcumin | BCL2 |
| L-Asparagine | GABBR2 |
| L-Asparagine | GABBR1 |
| L-Asparagine | ABAT |
| L-Asparagine | SLC6A1 |
| L-Asparagine | GABRA1 |
| L-Asparagine | GABRB2 |
| L-Asparagine | GABRG2 |
| L-Asparagine | GABRA3 |
| L-Asparagine | GABRA2 |
| L-Asparagine | GABRR1 |
| L-Asparagine | SLC6A11 |
| L-Asparagine | SLC6A13 |
| Oxalic acid | FYN |
| Oxalic acid | LCK |
| Serotonin | HTR2B |
| Serotonin | HTR4 |
| Serotonin | HTR1B |
| Serotonin | HTR1D |
| Serotonin | DRD1 |
| Serotonin | HTR1A |
| Serotonin | HTR1E |
| Serotonin | HTR2A |
| Serotonin | HTR2C |
| Serotonin | SLC6A4 |
| Serotonin | DRD3 |
| Serotonin | ADORA3 |
| Serotonin | HTR7 |
| Serotonin | HTR6 |
| Serotonin | HTR5A |
| Serotonin | HTR3A |
| Serotonin | SLC6A3 |
| Serotonin | MTNR1A |
| Serotonin | MTNR1B |
| Serotonin | ADRA2A |
| Serotonin | ADRA2C |
| Serotonin | SLC6A2 |
| Serotonin | SIGMAR1 |
| Serotonin | FLT4 |
| Serotonin | IGF1R |
| Serotonin | KDR |
| Serotonin | ADRB1 |
| Serotonin | HRH1 |
| Serotonin | LTA4H |
| Serotonin | DRD2 |
| Serotonin | MPO |
| Serotonin | OPRK1 |
| Serotonin | HRH3 |
| Serotonin | ANPEP |
| Serotonin | HRH2 |
| Serotonin | FAAH |
| Serotonin | TRPV1 |
| Serotonin | ADRA2B |
| Serotonin | QDPR |
| Serotonin | PNMT |
| Serotonin | ADRA1B |
| Serotonin | ACHE |
| Serotonin | AR |
| Serotonin | NR3C2 |
| Serotonin | NR3C1 |
| Serotonin | PGR |
| Serotonin | CHRNA3 |
| Serotonin | CHRNB4 |
| Serotonin | AOC3 |
| Serotonin | MAOB |
| Serotonin | KAT2B |
| Serotonin | TRPM8 |
| Serotonin | MAOA |
| Serotonin | PLA2G2A |
| Serotonin | OPRM1 |
| Serotonin | AADAT |
| Serotonin | KYAT3 |
| Serotonin | NQO2 |
| Serotonin | DPP7 |
| Serotonin | CHRNA4 |
| Serotonin | ADRA1A |
| Serotonin | EGFR |
| Spathulenol | UGT2B7 |
| Spathulenol | HSD11B1 |
| Spathulenol | IDO1 |
| Spathulenol | PTGS1 |
| Spathulenol | PGR |
| Spathulenol | NR1H3 |
| Spathulenol | ICMT |
| Sucrose | CDK1 |
| Sucrose | HSP90AA1 |
| Sucrose | VEGFA |
| Sucrose | PSEN2 |
| Sucrose | PSENEN |
| Sucrose | NCSTN |
| Sucrose | APH1A |
| Sucrose | PSEN1 |
| Sucrose | APH1B |
| Sucrose | FGF1 |
| Sucrose | HPSE |
| Sucrose | FGF2 |
| Sucrose | LGALS4 |
| Sucrose | LGALS3 |
| Sucrose | LGALS8 |
| Sucrose | HTR2B |
| Sucrose | ADRA2A |
| Sucrose | ADRA2C |
| Sucrose | ADRA2B |
| Sucrose | DRD1 |
| Sucrose | DRD2 |
| Sucrose | ADRA1D |
| Sucrose | HTR2A |
| Sucrose | HTR2C |
| Sucrose | DRD3 |
| Sucrose | CYP2D6 |
| Sucrose | HTR6 |
| Sucrose | ADRA1A |
| Sucrose | HTR1B |
| Sucrose | FOLH1 |
| Sucrose | RORC |
| Sucrose | TRPV1 |
| Sucrose | STAT3 |
| Thymol | TRPA1 |
| Thymol | PTGS1 |
| Thymol | GABRA1 |
| Thymol | GABRB2 |
| Thymol | GABRG2 |
| Thymol | HTR2B |
| Thymol | GABRB3 |
| Thymol | SLC6A2 |
| Thymol | HTR2C |
| Thymol | TYR |
| Thymol | CA2 |
| Thymol | SLC6A3 |
| Thymol | CHRM2 |
| Thymol | CHRM1 |
| Thymol | ALB |
| Thymol | FLT3 |
| Thymol | ACHE |
| Thymol | JAK1 |
| Thymol | JAK2 |
| Thymol | PRKCA |
| Thymol | AURKA |
| Thymol | CDK2 |
| Thymol | CCNA1 |
| Thymol | CCNA2 |
| Thymol | ESRRG |
| Thymol | SLC6A4 |
| Tryptamine | HTR1B |
| Tryptamine | HTR1D |
| Tryptamine | HTR2C |
| Tryptamine | SLC6A4 |
| Tryptamine | SLC6A3 |
| Tryptamine | MPO |
| Tryptamine | HTR6 |
| Tryptamine | HTR2B |
| Tryptamine | HTR2A |
| Tryptamine | HTR1A |
| Tryptamine | ADRA2A |
| Tryptamine | ADRB1 |
| Tryptamine | HTR1E |
| Tryptamine | SLC6A2 |
| Tryptamine | HTR7 |
| Tryptamine | HTR5A |
| Tryptamine | HTR4 |
| Tryptamine | ADORA3 |
| Tryptamine | DRD3 |
| Tryptamine | ADRA1A |
| Tryptamine | DRD2 |
| Tryptamine | SIGMAR1 |
| Tryptamine | TRPM8 |
| Tryptamine | ADRA2C |
| Tryptamine | CA3 |
| Tryptamine | CA6 |
| Tryptamine | CA5B |
| Tryptamine | CA5A |
| Tryptamine | CA13 |
| Tryptamine | HRH1 |
| Tryptamine | MTNR1A |
| Tryptamine | MTNR1B |
| Tryptamine | DRD1 |
| Tryptamine | KIF11 |
| Tryptamine | ICMT |
| Tryptamine | DPP7 |
| Tryptamine | DPP4 |
| Tryptamine | NISCH |
| Tryptamine | DNMT1 |
| Tryptamine | FLT4 |
| Tryptamine | IGF1R |
| Tryptamine | EGFR |
| Tryptamine | KDR |
| Tryptamine | LTA4H |
| Zederone | ADORA2B |
| Zederone | MTNR1A |
| Zederone | MTNR1B |
| Zederone | AR |
| Zederone | SLC6A3 |
| Zederone | ELANE |
| Zederone | EPHX1 |
| Zederone | PTAFR |
| Zederone | APP |
| Zedoarondiol | NR3C1 |
| Zedoarondiol | CYP19A1 |
| Zedoarondiol | PTGS2 |
| Zedoarondiol | AR |
| Zedoarondiol | ATP12A |
| Zedoarondiol | HSD11B1 |
| Zedoarondiol | FKBP1A |
| Zedoarondiol | NR3C2 |
| Zedoarondiol | PTAFR |
| Zedoarondiol | LRRK2 |
| Zedoarondiol | HSD11B2 |
| Zedoarondiol | BRD4 |
| Zedoarondiol | BRD2 |
| Zedoarondiol | PGR |
| Zedoarondiol | CDC7 |
| Zedoarondiol | SIGMAR1 |
| Zedoarondiol | TAAR1 |
| Zedoarondiol | APP |
| Zedoarondiol | CYP11B1 |
| Zedoarondiol | CYP11B2 |
| Zedoarondiol | CA2 |
| Zedoarondiol | CA7 |
| Zedoarondiol | CA1 |
| Zedoarondiol | CA3 |
| Zedoarondiol | CA6 |
| Zedoarondiol | CA12 |
| Zedoarondiol | CA14 |
| Zedoarondiol | CA9 |
| Zedoarondiol | CA4 |
| Zedoarondiol | CA13 |
| Zedoarondiol | CA5B |
| Zedoarondiol | NPC1L1 |
| Zedoarondiol | IL6 |
| Zedoarondiol | GLUL |
| Zedoarondiol | GPBAR1 |
| Zedoarondiol | FABP1 |
| Zedoarondiol | CDK5R1 |
| Zedoarondiol | CDK5 |
| Zedoarondiol | JAK3 |
| Zedoarondiol | JAK1 |
| Zedoarondiol | JAK2 |
| Zedoarondiol | TYK2 |
| Zedoarondiol | ABCC9 |
| Zedoarondiol | KCNJ11 |
| Zedoarondiol | FABP4 |
| Zedoarondiol | PPARA |
| Zedoarondiol | FABP3 |
| Zedoarondiol | FABP5 |
| Zedoarondiol | PTPN1 |
| Zedoarondiol | CD81 |
| Zedoarondiol | PTPN11 |
| Zedoarondiol | AKR1B10 |
| Zedoarondiol | HMOX1 |
| Zedoarondiol | NR1H4 |
| Zedoarondiol | FAAH |
| Zedoarondiol | CYP51A1 |
| Zedoarondiol | LIPA |
| Zedoarondiol | NR1H3 |
| Zedoarondiol | PTPRF |
| Zedoarondiol | PTPN2 |
| Zedoarondiol | PLA2G1B |
| Zedoarondiol | ACP1 |
| Zedoarondiol | G6PD |
| Zedoarondiol | CYP17A1 |
| Zedoarondiol | PDE10A |
| Zedoarondiol | SCD |
| Zedoarondiol | AOC3 |
| Zedoarondiol | KCNA5 |
| Zedoarondiol | SERPINA6 |
| Zedoarondiol | SHBG |
| Zerumin B | RPS6KA5 |
| Zerumin B | CDC25A |
| Zerumin B | OPRM1 |
| Zerumin B | OPRK1 |
| Zerumin B | JAK1 |
| Zerumin B | JAK2 |
| Zerumin B | JAK3 |
| Zerumin B | TYK2 |
| Zerumin B | PGR |
| Zerumin B | PIK3CG |
| Zerumin B | PIK3CA |
| Zerumin B | COMT |
| Zerumin B | CCNB3 |
| Zerumin B | CDK1 |
| Zerumin B | CCNB1 |
| Zerumin B | CCNB2 |
| Zerumin B | AR |
| Zerumin B | PSEN2 |
| Zerumin B | PSENEN |
| Zerumin B | NCSTN |
| Zerumin B | APH1A |
| Zerumin B | PSEN1 |
| Zerumin B | APH1B |
| Zerumin B | GABRB3 |
| Zerumin B | GABRG2 |
| Zerumin B | GABRA5 |
| Zerumin B | RASGRP1 |
| Zerumin B | PIM1 |
| Zerumin B | PIM3 |
| Zerumin B | MAPK14 |
| Zerumin B | HMGCR |
| Zerumin B | EGFR |
| Zerumin B | ICAM1 |
| Zerumin B | SELE |
| Zerumin B | ADA |
| Zerumin B | CDC25B |
| Zerumin B | PRKCA |
| Zerumin B | IDO1 |
| Zerumin B | PTGS1 |
| Zerumin B | PYGL |
| Zerumin B | CYP19A1 |
| Zerumin B | TRPA1 |
| Zerumin B | LIPE |
| Zerumin B | RBP4 |
| Zerumin B | NPY5R |
| Zerumin B | PPP2CA |
| Zerumin B | NOS1 |
| Zerumin B | CA2 |
| Zerumin B | KCNH2 |
| Zerumin B | CA1 |
| Zerumin B | CA9 |
| Zerumin B | THRB |
| Zerumin B | TGFBR1 |
| Zerumin B | CHRNA3 |
| Zerumin B | CHRNB4 |
| Zerumin B | SYK |
| Zerumin B | ZAP70 |
| Zerumin B | STAT5A |
| Zerumin B | PRKCD |
| Zerumin B | CYP11B1 |
| Zerumin B | CXCR2 |
| Zerumin B | CYP11B2 |
| Zerumin B | CSNK1D |
| Zerumin B | CX3CR1 |
| Zerumin B | CSNK1E |
| Zerumin B | ROCK2 |
| Zerumin B | ROCK1 |
| Zerumin B | DUT |
| Zerumin B | GBA |
| Zerumin B | MAPK11 |
| Zerumin B | MAP4K4 |
| Zerumin B | MAOB |
| Zerumin B | PRKCB |
| Zerumin B | PRKCE |
| Zerumin B | PRKCH |
| Zerumin B | PRKCQ |
| Zerumin B | BRD4 |
| Zerumin B | CHRM4 |
| Zerumin B | CHRM5 |
| Zerumin B | CHRM2 |
| Zerumin B | CHRM1 |
| Zerumin B | CHRM3 |
| Zerumin B | CYP2C9 |
| Zerumin B | KCNA5 |
| Zerumin B | PLA2G2A |
| Zerumin B | EPHX2 |
| Zerumin B | XIAP |
| Zerumin B | S1PR1 |
| Zerumin B | CHEK1 |
| Zerumin B | PLA2G1B |
| Zerumin B | HLCS |
| Zerumin B | PDE2A |
| Zerumin B | PARP1 |
| Zerumin B | IL6ST |
| Zerumin B | GRM5 |
| Zerumin B | PER2 |
| Zerumin B | PDE10A |
| Zerumin B | STAT3 |
| Zerumin B | EPHX1 |
| Zerumin B | MKNK1 |
| Zerumin B | TYRO3 |
| Zerumin B | MAPK8 |
| Zerumin B | MPO |
| Zerumin B | CCR5 |
| Zerumin B | GPR88 |
| Zerumin B | MAPK1 |
| Zerumin B | PRKACA |
| Zerumin B | SCN9A |
| Zerumin B | RPS6KB1 |
| Zerumin B | ADRA2C |
| Acetylursolic acid | PTPN1 |
| Acetylursolic acid | HSD11B1 |
| Acetylursolic acid | PTPN2 |
| Acetylursolic acid | PTGS2 |
| Acetylursolic acid | HSD17B2 |
| Acetylursolic acid | HSD11B2 |
| Acetylursolic acid | PTGES |
| Acetylursolic acid | FNTA |
| Acetylursolic acid | FNTB |
| Acetylursolic acid | CYP19A1 |
| Acetylursolic acid | CYP17A1 |
| Acetylursolic acid | CES2 |
| Acetylursolic acid | SHBG |
| Acetylursolic acid | CYP2C19 |
| Acetylursolic acid | AKR1C3 |
| Acetylursolic acid | AKR1C2 |
| Acetylursolic acid | EPAS1 |
| Acetylursolic acid | F2 |
| Acetylursolic acid | PRSS1 |
| Acetylursolic acid | HMGCR |
| Acetylursolic acid | NR3C1 |
| Acetylursolic acid | PLA2G2A |
| Acetylursolic acid | SERPINA6 |
| Acetylursolic acid | PDE4D |
| Acetylursolic acid | AR |
| Acetylursolic acid | AKR1B10 |
| Acetylursolic acid | POLB |
| Acetylursolic acid | PLA2G1B |
| Acetylursolic acid | CDC25B |
| Acetylursolic acid | RORC |
| Acetylursolic acid | PTPRF |
| Acetylursolic acid | ACP1 |
| Acetylursolic acid | NOS2 |
| Acetylursolic acid | ATP12A |
| Acetylursolic acid | NR1H3 |
| Acetylursolic acid | ALOX5 |
| Acetylursolic acid | BCL2L1 |
| Acetylursolic acid | PRKCA |
| Acetylursolic acid | PRKCD |
| Acetylursolic acid | PRKCQ |
| Acetylursolic acid | PREP |
| Acetylursolic acid | PTGIR |
| Acetylursolic acid | NR3C2 |
| Acetylursolic acid | PGR |
| Acetylursolic acid | PTGDR2 |
| Acetylursolic acid | NLRP3 |
| Acetylursolic acid | ALOX5AP |
| Acetylursolic acid | EDNRB |
| Acetylursolic acid | AGTR1 |
| Acetylursolic acid | PTGER2 |
| Acetylursolic acid | MC4R |
| Acetylursolic acid | MC1R |
| Acetylursolic acid | MC5R |
| Acetylursolic acid | PPARG |
| Acetylursolic acid | PPARA |
| Acetylursolic acid | PPARD |
| Acetylursolic acid | POLA1 |
| Acetylursolic acid | JUN |
| Acetylursolic acid | FAAH |
| Epijasminoside A | IL2 |
| Epijasminoside A | SLC5A2 |
| Epijasminoside A | HSD11B2 |
| Epijasminoside A | HSD11B1 |
| Epijasminoside A | OGA |
| Epijasminoside A | SLC29A1 |
| Epijasminoside A | SLC5A1 |
| Epijasminoside A | F2 |
| Epijasminoside A | ADK |
| Epijasminoside A | ADA |
| Epijasminoside A | TK1 |
| Epijasminoside A | STAT3 |
| Epijasminoside A | HSPA8 |
| Epijasminoside A | GLI1 |
| Epijasminoside A | CDK2 |
| Epijasminoside A | CCNA1 |
| Epijasminoside A | CCNA2 |
| Epijasminoside A | ADORA2A |
| Epijasminoside A | HDAC1 |
| Epijasminoside A | ADORA3 |
| Epijasminoside A | GSTP1 |
| Epijasminoside A | GSTM2 |
| Epijasminoside A | FUCA1 |
| Epijasminoside A | SLC28A2 |
| Epijasminoside A | EGFR |
| Epijasminoside A | PNP |
| Epijasminoside A | MAPK10 |
| Epijasminoside A | GBA |
| Epijasminoside A | GAA |
| Epijasminoside A | HSPA5 |
| Epijasminoside A | FOLH1 |
| Epijasminoside A | HK2 |
| Epijasminoside A | HK1 |
| Epijasminoside A | TYR |
| Epijasminoside A | PPP1CC |
| Epijasminoside A | PPP2CA |
| Epijasminoside A | PPP2R5A |
| Epijasminoside A | SLC5A4 |
| Epijasminoside A | AHCYL1 |
| Epijasminoside A | GAPDH |
| Epijasminoside A | JAK3 |
| Epijasminoside A | MME |
| Epijasminoside A | PYGL |
| Epijasminoside A | GRK1 |
| Epijasminoside A | AHCY |
| Epijasminoside A | FLT1 |
| Epijasminoside A | KIT |
| Epijasminoside A | PTPN1 |
| Epijasminoside A | GLB1 |
| Epijasminoside A | AR |
| Epijasminoside A | AMPD3 |
| Epijasminoside A | PYGM |
| Epijasminoside A | MMP9 |
| Epijasminoside A | NR3C1 |
| Epijasminoside A | MMP13 |
| Epijasminoside A | MMP1 |
| Epijasminoside A | MMP7 |
| Epijasminoside A | MMP12 |
| Epijasminoside A | MMP8 |
| Epijasminoside A | BCL2L1 |
| Epijasminoside A | CPT1A |
| Epijasminoside A | MGAM |
| Epijasminoside A | HAO2 |
| Epijasminoside A | CPT1B |
| Epijasminoside A | SI |
| Epijasminoside A | UPP1 |
| Epijasminoside A | CXCR2 |
| Gardaloside | FUCA1 |
| Gardaloside | ADORA1 |
| Gardaloside | ADORA2A |
| Gardaloside | TYR |
| Gardaloside | LGALS3 |
| Gardaloside | LGALS9 |
| Gardaloside | CA2 |
| Gardaloside | CA1 |
| Gardaloside | CA12 |
| Gardaloside | CA9 |
| Gardaloside | TREH |
| Gardaloside | AKR1C3 |
| Gardaloside | FOLH1 |
| Gardaloside | ADORA3 |
| Gardaloside | CA14 |
| Gardaloside | MGAM |
| Gardaloside | SI |
| Gardaloside | EPHX2 |
| Gardaloside | HK2 |
| Gardaloside | HK1 |
| Gardaloside | HPRT1 |
| Gardaloside | CA7 |
| Gardaloside | CA4 |
| Gardaloside | CA13 |
| Gardaloside | CA5A |
| Gardaloside | IGFBP3 |
| Gardaloside | CDA |
| Geniposide | ADORA1 |
| Geniposide | ADORA2A |
| Geniposide | CA2 |
| Geniposide | CA1 |
| Geniposide | CA12 |
| Geniposide | CA9 |
| Geniposide | CA14 |
| Geniposide | ADORA3 |
| Geniposide | LGALS3 |
| Geniposide | LGALS9 |
| Geniposide | SLC5A2 |
| Geniposide | SLC29A1 |
| Geniposide | ADORA2B |
| Geniposide | CA7 |
| Geniposide | CA13 |
| Geniposide | EPHX2 |
| Geniposide | ADK |
| Geniposide | HK2 |
| Geniposide | HK1 |
| Geniposide | AKR1B1 |
| Geniposide | FUCA1 |
| Geniposide | TYR |
| Geniposide | HSPA8 |
| Geniposide | ADA |
| Geniposide | TYMP |
| Geniposide | OGA |
| Geniposide | LGALS4 |
| Geniposide | GBA |
| Geniposide | AKR1C3 |
| Geniposide | LGALS8 |
| Geniposide | EIF4H |
| Geniposide | PABPC1 |
| Geniposide | MMP2 |
| Geniposide | SLC28A2 |
| Geniposide | FOLH1 |
| Geniposide | HSPA5 |
| Geniposide | SLC5A4 |
| (-)-Blumealactone C | MYB |
| (-)-Blumealactone C | RELA |
| (-)-Blumealactone C | UBE2D3 |
| (-)-Blumealactone C | ADCY2 |
| (-)-Blumealactone C | NR0B1 |
| (-)-Blumealactone C | ADCY1 |
