# Supplementary material for: Uncovering the Mechanism of the Xingnaojing Injection against Ischemic Stroke Using a Combined Network Pharmacology Approach and Gut Microbiota Analysis
Source: Evid Based Complement Alternat Med. 2022 May 20;2022:5886698. doi: 10.1155/2022/5886698 (PMC9142292; doi:10.1155/2022/5886698)
Supplement: Supplementary Materials — Supplementary Table S1: active ingredients of XNJ identified by UHPLC-MS. Supplementary Table S2: 507 compound-related targets. Supplementary Table S3: 1667 IS-related targets and 2074 IBD-related targets. Supplementary Table S4: 210 shared targets were identified as potential therapeutic targets of XNJ against IS. . [file 5886698.f1.zip › 5886698.f1/S3 disease targets.docx]

| IS targets | IBD targets |
| --- | --- |
| GLA | IL10 |
| APP | TNF |
| COL3A1 | IL23R |
| ITM2B | IRGM |
| F2 | ATG16L1 |
| F5 | NOD2 |
| NOTCH3 | CARD9 |
| ADAMTS13 | TNFSF15 |
| HDAC9 | INAVA |
| CDKN2B-AS1 | IL18RAP |
| LDLR | PTPN22 |
| ALDH2 | MUC19 |
| ABO | XIAP |
| ZFHX3 | ICAM1 |
| ACTA2 | IL6 |
| PROCR | IL10RA |
| PDE3A | PLCG2 |
| SLCO1B1 | PTGS2 |
| COL4A1 | STAT3 |
| LOX | TGFB1 |
| SMAD3 | APC |
| ABCC1 | IL10RB |
| PRKG1 | IL2RA |
| TGFBR2 | SLC11A1 |
| C20orf181 | MAGI2 |
| SH2B3 | DEFA5 |
| PMF1-BGLAP | RASSF1 |
| PATJ | ITGA4 |
| CDK6 | SFRP2 |
| CDKN1A | APC2 |
| CDKN2C | ITGAL |
| CERNA3 | SFRP1 |
| PRPF8 | VNN1 |
| DINOL | HPS1 |
| CHD3 | IL2 |
| FAF1 | ITGB8 |
| PMF1 | SLAMF8 |
| NLRP3 | CUL2 |
| APOA5 | XBP1 |
| AGBL1 | MUC2 |
| CRP | NOS2 |
| CYP2C19 | ESR2 |
| DAB1 | RUNX3 |
| ACE | CD40LG |
| AGT | NFIL3 |
| AGTR1 | SLC6A14 |
| EDN1 | F5 |
| ELN | HLA-B |
| ENG | HLA-DRB1 |
| FBN1 | IGF2 |
| FGB | KLKB1 |
| FOXE3 | PTPN2 |
| LRCH1 | SLC22A4 |
| TSPAN15 | MST1 |
| ALOX5 | NUDT15 |
| ALOX5AP | CCL20 |
| NAALADL2 | STAT4 |
| PCSK9 | IL27 |
| SLC26A11 | LRRK2 |
| F11-AS1 | FUT2 |
| APOB | HLA-DQA1 |
| TSPAN33 | PRDM1 |
| APOE | TYK2 |
| IL1B | TNFRSF6B |
| IL1RN | IL18R1 |
| IL6 | TRAF3IP2 |
| IL10 | FCGR2A |
| ILF3 | NR5A2 |
| KCNK1 | GPR35 |
| KCNK3 | SMAD3 |
| C10orf143 | ITLN1 |
| LPA | SLC22A23 |
| LPL | GPR65 |
| MIR146A | IFNG-AS1 |
| MAT2A | CREM |
| MMP9 | LACC1 |
| MTHFR | PRKCB |
| MYH11 | ZMIZ1 |
| MYLK | CD226 |
| NINJ2 | ADCY3 |
| NOS3 | SP140 |
| SERPINE1 | ADCY7 |
| PCDH7 | CDYL2 |
| PCYT1A | SNX20 |
| PDE4D | MAP3K8 |
| PIK3CA | GPR183 |
| PIK3CB | CELSR3 |
| PIK3CD | ETS1 |
| PIK3CG | IFNGR2 |
| PLAT | NFATC1 |
| PLG | TET2 |
| PON1 | BANK1 |
| PPARG | FERMT1 |
| CASZ1 | TMBIM1 |
| KIF26B | ERAP2 |
| AVP | SHC1 |
| PROC | CRTC3 |
| SLC44A2 | FTO |
| PTGS2 | BSN |
| PTPRD | PYGO2 |
| PTPRG | IL1RL1 |
| NTN4 | LINC00484 |
| BDNF | SPATA48 |
| ATXN2 | SH2B3 |
| SMARCA4 | RPSAP35 |
| FUNDC2 | BOK-AS1 |
| SON | RTEL1-TNFRSF6B |
| STAT3 | SCAMP3 |
| TGFB2 | C5orf66 |
| TGFB3 | CRTC3-AS1 |
| TGFBR1 | LINC00824 |
| TLR4 | LINC02745 |
| TNF | GLYAT |
| VEGFA | NOD1 |
| VWF | TAB2-AS1 |
| MFAP5 | RGS14 |
| SLC2A10 | LINC02128 |
| QRICH2 | CIT |
| RUNX1 | CDC37 |
| ALDH1A2 | ATXN2L |
| HPS4 | PARK7 |
| COX7A2L | NLRP3 |
| ADIPOQ | RMI2 |
| PLAA | CCR5 |
| ACVRL1 | ZNF831 |
| CDC5L | GALM |
| EPO | ZNF300P1 |
| HMGB1 | CCDC26 |
| MMP3 | CRP |
| MIR499A | LINC01620 |
| SELP | CTLA4 |
| PLA2G7 | HORMAD2 |
| CYP4F2 | PUS10 |
| IGF1 | AMZ1 |
| CXCL8 | DAP |
| IL17A | DENND1B |
| AQP4 | FAM171B |
| LTA | DNMT3B |
| NFE2L2 | SLC39A11 |
| P2RY12 | EPHB4 |
| EPHX2 | ALB |
| ESR1 | PTK2B |
| FGFR1 | FEN1 |
| MMRN1 | SBNO2 |
| GABPA | TAB2 |
| HIF1A | TBC1D9 |
| IL1A | FOSL2 |
| MIR126 | IPMK |
| MIR145 | SPDEF |
| OLR1 | GALC |
| CCL2 | PNKD |
| CXCL12 | SH2B1 |
| SELE | GCKR |
| CDKN2A | ACAD8 |
| CETP | FOXP1 |
| CYP11B2 | IL37 |
| AGER | GNA12 |
| SIRT1 | GPR18 |
| ICAM1 | BTBD8 |
| IL18 | HGFAC |
| ITGA2 | HLA-DQA2 |
| JAK2 | HMGB1 |
| MIR223 | IBD2 |
| TNFRSF11B | UBAC2 |
| TREM2 | DUPD1 |
| PTGS1 | C17orf67 |
| TGFB1 | IFNG |
| CASP3 | IL1A |
| PPIG | IL1B |
| SIGMAR1 | IL1RN |
| CPB2 | IL4 |
| ABCA1 | CXCL8 |
| MTOR | IL9 |
| GPX3 | IL12B |
| IL4 | IL17A |
| LGALS3 | IL18 |
| MMP1 | IRF6 |
| MMP2 | ITPA |
| NGF | PSMG4 |
| GP6 | GRID2IP |
| ABCB1 | LCN2 |
| PRKCH | EEF1AKMT2 |
| MAPK3 | C1orf141 |
| RETN | TEX41 |
| BRCA1 | LPP |
| THBD | SMAD7 |
| TP53 | ARSA |
| APLN | C10orf55 |
| KALRN | MEFV |
| KL | IRF1-AS1 |
| HDAC6 | MTHFR |
| CDKN2B | MYO9B |
| MTCO2P12 | NFKB1 |
| COL4A2 | NOTCH1 |
| COX8A | DELEC1 |
| PARP1 | IL22 |
| CSF3 | IMPG2 |
| CST3 | IBD5 |
| CYP4A11 | FOXP3 |
| ENO2 | TUBD1 |
| F3 | IL23A |
| FABP4 | HDAC7 |
| SIRT3 | SUFU |
| ALOX15 | ABCB1 |
| ANGPT1 | PLA2G4A |
| NRG1 | PLAU |
| HMOX1 | TREM1 |
| HSPA4 | PPARG |
| APOC3 | CDKAL1 |
| IFNB1 | INTS11 |
| ITGA2B | LRRC8D |
| ITGB3 | MIR3936HG |
| MALAT1 | CDC42SE2 |
| KLK1 | ERGIC1 |
| KNG1 | SLAIN2 |
| LCN2 | IL21 |
| LEP | RIT1 |
| MIR122 | BRD2 |
| MIR143 | BACH2 |
| MIR149 | S100A12 |
| MMP12 | ATXN2 |
| MPO | THADA |
| COX2 | IFIH1 |
| SERPINC1 | TRAF3IP2-AS1 |
| NEFL | LINC00598 |
| NOS2 | SLC22A5 |
| NPY | TG |
| PON2 | TIMP1 |
| PPARA | TLR2 |
| CHDH | TLR4 |
| MAPK1 | TP53 |
| REN | TPMT |
| SPP1 | UBE2L3 |
| TRAF6 | VDR |
| UCP2 | DAP3 |
| VKORC1 | ANKRD55 |
| CAV1 | SEMA6D |
| CBS | CPEB4 |
| IL33 | NDFIP1 |
| GDF15 | DUSP16 |
| CD40 | CAMK2A |
| DNM1L | TSPAN14 |
| CDK5 | GATD3A |
| TRIB1 | SYN3 |
| KLF2 | KIAA1109 |
| ADAMTS7 | MAML2 |
| SERPINA3 | CUL1 |
| CX3CR1 | ZGPAT |
| CYBA | KSR1 |
| CYP2C9 | NR1I2 |
| CYP2J2 | SKAP2 |
| DPP4 | CCND3 |
| EDNRB | IL33 |
| F2R | PLXNA4 |
| F11 | OSMR |
| F13A1 | ZNF300 |
| FGA | CD6 |
| SIRT2 | DLG5 |
| POLDIP2 | CD14 |
| GRIN2A | CD28 |
| GSK3B | LPXN |
| HFE | FADS2 |
| HLA-DRB1 | LITAF |
| APOA1 | PHACTR2 |
| IL6R | TM9SF4 |
| IL13 | NKX2-3 |
| LGALS1 | AHR |
| LGALS2 | IBD3 |
| LIF | JAK2 |
| LTC4S | MIR155 |
| MIR130A | MMP9 |
| MIR137 | TLR9 |
| MIR155 | SLC15A1 |
| MIR19A | TAC1 |
| MIR21 | CLDN2 |
| MIR210 | CSF2 |
| MIR30A | DPP4 |
| MAP3K5 | NOX1 |
| MIF | HIF1A |
| MMP8 | IL13 |
| MMP10 | IL15 |
| COX1 | IRF5 |
| NOS1 | MIR21 |
| P2RY1 | COX2 |
| ANGPTL4 | DUOX2 |
| TNFRSF12A | HAMP |
| RNLS | MICA |
| MEG3 | PSC |
| SELENOS | IL17F |
| PTX3 | F2 |
| BCL2 | HPGDS |
| S100B | LEP |
| ACSM3 | MPO |
| CCL11 | MAPK1 |
| SELPLG | MYDGF |
| SLC1A2 | CCL2 |
| SLC25A1 | STAT6 |
| BSG | TLR5 |
| CCR2 | CXCR4 |
| XBP1 | MADCAM1 |
| XRCC1 | MTCO2P12 |
| CAD | CYBB |
| SNHG12 | AGT |
| TP63 | NR0B1 |
| SOCS1 | CARD8 |
| KCNK17 | GP2 |
| NTN1 | NR3C1 |
| ROCK2 | GSTM1 |
| PTGES | IGF1 |
| CD40LG | MIR146A |
| CELSR1 | MBL2 |
| ARHGEF10 | MIF |
| MIR874 | MPZ |
| OCLN | P2RX7 |
| MIR499B | IL17D |
| PPIF | PMP22 |
| PGR-AS1 | IMPACT |
| NAMPT | TTC7A |
| CBSL | S100A8 |
| AHSA1 | S100A9 |
| SLC17A3 | BPI |
| HPSE | RIPK1 |
| RUVBL2 | RABEPK |
| CHI3L1 | LANCL1 |
| HSPA12B | CCR9 |
| ADD1 | EBNA1BP2 |
| RBM45 | H3P28 |
| ADM | MTFMT |
| CPOX | CCR6 |
| CRK | ACE |
| MAPK14 | DECR1 |
| CTLA4 | DEFB4A |
| ADRA2B | FCGR3A |
| ADRB2 | SIRT1 |
| CYP1A1 | GH1 |
| CYP2B6 | GSTT1 |
| CYP2C8 | GUCY2C |
| CYP3A4 | HNF4A |
| CYP3A5 | ARMH1 |
| NQO1 | IL7 |
| AGTR2 | CXCL10 |
| APLNR | KRAS |
| ECE1 | KRT8 |
| EDNRA | RPSA |
| EGR1 | MIR223 |
| AHSG | MMP1 |
| MLKL | MUC1 |
| EPHA4 | MYLK |
| EPHB2 | IBD6 |
| ESR2 | PSMD7 |
| ALB | REG1A |
| EZH2 | SLC9A3 |
| F12 | TACR1 |
| FCGR2A | TFF3 |
| FGFR4 | CAT |
| FGG | TP63 |
| DKK1 | ARHGEF2 |
| RCOR1 | ENTPD1 |
| SNHG1 | CD40 |
| ALOX12 | LRBA |
| POU2F3 | NAT2 |
| RNF19A | CD24 |
| GCG | DEFB4B |
| GFAP | EBI3 |
| GJA4 | AGR2 |
| IL37 | FGL2 |
| ANGPTL3 | SLCO6A1 |
| GLP1R | DMBT1 |
| GPER1 | AGER |
| GRIA2 | ESR1 |
| GRIN1 | EZH2 |
| NR3C1 | F2RL1 |
| CXCL2 | FCGR3B |
| HABP2 | FGF7 |
| HDAC2 | NLRP1 |
| CFH | FOLH1 |
| HGF | MTOR |
| HSPA1A | SETD2 |
| HSPA1B | HGF |
| HSPB1 | HSPA4 |
| HSPB2 | IL11 |
| IFNG | IL16 |
| APOH | GSTK1 |
| IL9 | NPSR1 |
| IL15 | LYZ |
| IMPA2 | MLH1 |
| AQP9 | MMP3 |
| IRF4 | NFE2L2 |
| AR | OSM |
| JUN | PDCD1 |
| KCNJ13 | PROC |
| KCNQ1 | PTGER4 |
| ARG1 | RAC1 |
| FADS1 | BCL2 |
| LLGL2 | S100B |
| LTA4H | CCL11 |
| MIR106B | CX3CL1 |
| MIR107 | IL25 |
| MIR125A | SOAT1 |
| MIR132 | TCF4 |
| MIR134 | TLR3 |
| MIR150 | TNFAIP6 |
| MIR15A | VEGFA |
| MIR181C | VIP |
| MIR27B | WAS |
| MIR29B1 | REG4 |
| MIR29B2 | TNFRSF25 |
| MIR17HG | ADIPOQ |
| TNFSF12-TNFSF13 | CD68 |
| ARSA | CDKN2B-AS1 |
| MIP | RAB4B-EGLN2 |
| ACHE | LINC-ROR |
| MMP7 | TLR6 |
| MIR335 | CEACAM5 |
| MTR | CFTR |
| MTRR | IL24 |
| NUBP1 | CHI3L1 |
| ATM | EGLN2 |
| NGFR | LINC02605 |
| NOTCH1 | CNR1 |
| NPPA | CNR2 |
| NPR3 | RBM45 |
| NTF3 | DOCK11 |
| DUOX2 | CSF3 |
| CKLF | DEFB1 |
| CLEC1B | AGA |
| PDE4A | DMD |
| SIRT6 | ATN1 |
| SERPINF1 | DSG2 |
| PIN1 | EGF |
| PLA2G1B | EGFR |
| PLA2G2A | MAST3 |
| PLAU | PANX1 |
| SERPINF2 | GABPA |
| DUOX1 | GBP1 |
| PPARD | GCG |
| PPID | GJB2 |
| TUG1 | CXCR3 |
| MARCHF1 | GPX1 |
| MAPK8 | CD274 |
| PROS1 | GSTM2 |
| PSMA6 | IL19 |
| ACKR3 | HLA-DPB1 |
| PTEN | HMOX1 |
| PTGIS | NR4A1 |
| MIR494 | HSPD1 |
| NGB | IFNA1 |
| CXCL16 | IFNA13 |
| RAC1 | IGFBP3 |
| MOK | IL2RB |
| ACE2 | IL4R |
| RBP4 | IL6ST |
| BCL2A1 | CXCR2 |
| RELA | INSRR |
| HPSE2 | IRF4 |
| GAS5 | ISG20 |
| SGK1 | ITGAE |
| RTN4R | ITGAX |
| SLC5A2 | LGALS3 |
| SLC9A1 | LTA |
| SLC12A2 | MIR31 |
| SST | MMP2 |
| STC1 | MMP13 |
| SULT1E1 | MUC5AC |
| SYK | NM |
| TBXAS1 | TNFRSF11B |
| HNF1A | SERPINE1 |
| TGFA | CUZD1 |
| TSPO | ERAP1 |
| TIMP2 | PHB |
| TLR2 | PI3 |
| TNFSF4 | PLA2G1B |
| TXN | PON1 |
| VCAM1 | IL26 |
| TRPV1 | LANCL2 |
| CACNA1A | MAPK8 |
| CXCR4 | BTNL2 |
| AIMP2 | MUC13 |
| NEIL1 | PTEN |
| NR4A3 | AICDA |
| PDGFD | TRPV4 |
| COASY | CCND1 |
| COL18A1 | REN |
| PLA2G6 | ACTB |
| ULK1 | RORC |
| TSLP | CCL5 |
| BECN1 | CXCL12 |
| RIPK1 | SELENOP |
| TNFSF12 | SFTPD |
| HSPB3 | SOD1 |
| MCU | ADAM17 |
| TIMD4 | TCF7L2 |
| SLC33A1 | TIMP3 |
| CD14 | TLR1 |
| COX5A | TNFRSF1A |
| GRAP2 | TRAF6 |
| ABCG2 | VIPR1 |
| GSTO1 | BEST1 |
| AIM2 | XDH |
| EIF2AK3 | NLRX1 |
| CARTPT | CASP1 |
| ABCG1 | CASP3 |
| CD69 | MBTPS1 |
| SEMA3E | TNFSF14 |
| MAFB | CD1D |
| CDC42 | IL32 |
| ZGLP1 | G6PC3 |
| MIR298 | CD44 |
| MIR922 | NR1I3 |
| KIR2DS2 | ADA |
| DEFB4B | TANK |
| MIR1306 | SMIM25 |
| MIR1203 | KLRC4-KLRK1 |
| MIR664A | NAMPT |
| HOTTIP | CDKN2A |
| MIR4437 | CCL26 |
| MIR4669 | CDX2 |
| MIR4656 | UBD |
| NR1H3 | PERCC1 |
| EDIL3 | CXCR6 |
| PTCSC3 | TNFSF13B |
| CDH13 | LILRB1 |
| EBI3 | IMMT |
| EMSLR | RIPK3 |
| LOC101929707 | CHGA |
| CDKN3 | EGLN3 |
| IRF9 | PRRT2 |
| ERICD | IL22RA2 |
| ATG7 | MRGPRX3 |
| AGR2 | MRGPRX4 |
| GNLY | CMA1 |
| SORBS1 | GPR151 |
| CAMKK2 | ADORA2B |
| CXCR6 | CRH |
| MASP2 | ADORA3 |
| TRAF3IP2 | HT |
| NES | MAPK14 |
| PLK2 | CSF1 |
| ALDH1L1 | CCN2 |
| LILRB1 | GPBAR1 |
| RNPS1 | CX3CR1 |
| FASTK | CYP2D6 |
| PDIA5 | CYP3A4 |
| STIP1 | CBLL2 |
| COPS5 | OXER1 |
| RIPK3 | DNTT |
| LOC110673971 | SLC26A3 |
| DUSP14 | DUSP1 |
| CAPN10 | EDN1 |
| CHIT1 | ELANE |
| TREX1 | EPAS1 |
| PARK7 | AKT1 |
| VSIG4 | F2R |
| ACOT7 | F3 |
| MGLL | GPRC6A |
| CHRNA4 | FHIT |
| TRIM9 | KLRK1 |
| CIRBP | ACSBG1 |
| H3P7 | SATB2 |
| ADCYAP1 | FN1 |
| LRG1 | LPAR3 |
| CYP2R1 | FPR2 |
| ACOT4 | SMUG1 |
| CCR5 | ALOX15 |
| FOPNL | ALPI |
| TAF8 | GAST |
| UBR3 | MRGPRX1 |
| COMT | GAPDH |
| CRHR1 | GC |
| CRMP1 | GDNF |
| DEFB104A | GEM |
| TRPM6 | CBLIF |
| SRXN1 | GJA1 |
| CRYZ | IL17C |
| CSF2 | GLB1 |
| TTC7B | GLI1 |
| ADRA1A | SGSM3 |
| CILP2 | GPR3 |
| NKX2-5 | NEAT1 |
| HJV | FFAR2 |
| CTNNB1 | GPX2 |
| ADRA2A | TBX21 |
| ARL6IP6 | ACACA |
| RMDN2 | HLA-C |
| ADRA2C | CELIAC2 |
| ADRB1 | HP |
| C5orf38 | HSD11B1 |
| CYP2B7P | HSD11B2 |
| CYP2D6 | BIRC3 |
| CYP2E1 | HSPA1A |
| CYP24A1 | HSPA1B |
| DECR1 | HSP90AA1 |
| DEFB1 | APOA4 |
| DEFB4A | IRF8 |
| DLD | IDH1 |
| DLG2 | IFNGR1 |
| DVL1 | LINC01193 |
| EDN2 | IL1R1 |
| EDN3 | IL6R |
| EEF1A1 | IL13RA2 |
| EFNB2 | IDO1 |
| CELSR2 | AQP6 |
| EGR2 | INPP5D |
| EGR4 | ITGAM |
| RMST | JAK3 |
| A2M | KIR2DS1 |
| ELK1 | KIR3DL1 |
| EPAS1 | KIF22 |
| ERCC4 | LGALS9 |
| ETS1 | MIR122 |
| ETV3 | MIR126 |
| ABCD1 | MIR132 |
| F7 | MIR150 |
| F8 | MIR15A |
| F9 | MIR19B1 |
| FBLN1 | MIR200B |
| FCGR3A | MIR224 |
| FCGR3B | MEP1A |
| FCN1 | MICB |
| FGF1 | MMP7 |
| FGF2 | MMP14 |
| ABCD2 | MOG |
| FGF13 | VN1R17P |
| VEGFD | GPR166P |
| MRAS | MIR135B |
| VASH1 | MUC4 |
| NTNG1 | MUC6 |
| CARD8 | MYD88 |
| ATF6 | ATF3 |
| FOXF2 | CEACAM6 |
| FAIM2 | NCF4 |
| FOXO3 | NGF |
| MCF2L2 | NOS3 |
| FLG | SLC11A2 |
| TBC1D9 | MIR375 |
| MLC1 | PAEP |
| ARC | CD207 |
| CLEC16A | PAK1 |
| FMO3 | REG3A |
| CRTC1 | PRKN |
| NCS1 | HSPA14 |
| TRS-AGA2-3 | TLR8 |
| FOLH1 | PDE4A |
| SPIDR | ACP1 |
| FOS | PIM1 |
| MMD | PIK3CD |
| MAPK8IP2 | PKM |
| PADI4 | PLG |
| SMUG1 | POMC |
| DAPK2 | PON2 |
| PLA2G15 | SLC52A1 |
| BCL2L13 | FBXW7 |
| ABCA4 | PRKCA |
| PANX1 | PRTN3 |
| G6PD | RETN |
| PRDX5 | PTGS1 |
| HECTD1 | PTPN11 |
| GALNT2 | RAC2 |
| HSPA12A | RAG2 |
| FAM98A | RARRES2 |
| GAP43 | LGR6 |
| IS1 | REG1B |
| COPD | RELA |
| GATA1 | BCL3 |
| GATA3 | RNASE3 |
| GCH1 | IGAN1 |
| GCKR | GAS5 |
| SLC17A5 | BDNF |
| NPAS4 | S100A1 |
| GEM | S100A4 |
| HAVCR1 | SAA1 |
| GGCX | SCN10A |
| GHRH | CCL25 |
| GJA1 | CXCL11 |
| NAAA | CXCL5 |
| IL17B | SDC1 |
| GCLC | SELE |
| STK39 | SELL |
| GLI2 | SGK1 |
| HTRA2 | CLEC7A |
| GNAS | GORASP1 |
| GNB3 | BMI1 |
| GOT2 | WNK1 |
| GPI | SPP1 |
| H19 | ST14 |
| ANGPT2 | STAT1 |
| SNHG15 | STATH |
| ANK1 | SYT1 |
| XKR6 | TRBV20OR9-2 |
| GPR37 | TFF1 |
| GPR42 | TFRC |
| GPT | TIMP2 |
| GPX1 | TNFAIP3 |
| REM1 | TNFRSF1B |
| GRN | HSP90B1 |
| GRIK2 | VCAM1 |
| ANPEP | VDAC1 |
| GRIN2B | VEGFC |
| GRIN2C | EZR |
| GRINA | VWF |
| SETD2 | MMP28 |
| GRM1 | MUL1 |
| GRM2 | RHBDF2 |
| CXCL1 | CAMP |
| GSTM1 | FZD4 |
| GSTT1 | ULK1 |
| UBQLN1 | CASP8 |
| NOP53 | CASP9 |
| ANXA1 | IL1F10 |
| HADHA | IKBKG |
| HCRT | TCAP |
| HDLBP | TNFSF11 |
| HK2 | ADAM15 |
| ACACA | RIPK2 |
| HLA-B | IL1RL2 |
| HLA-C | HDAC3 |
| HLA-DQB1 | CCK |
| HLA-G | SPHK1 |
| AOC2 | CLDN1 |
| NR4A1 | SLC16A4 |
| HP | SLC16A3 |
| MMAB | MSC |
| SERPINA9 | MS4A1 |
| HES1 | LONP1 |
| BIRC5 | ABCG2 |
| HSPG2 | AIM2 |
| HTR1A | ARHGEF6 |
| HTR2A | TBPL1 |
| FFAR4 | CLOCK |
| C1QTNF9 | SLC23A1 |
| ID2 | CDC42 |
| IFIT3 | MIR665 |
| ACTBL2 | C20orf181 |
| APOD | HDAC6 |
| MAGEB5 | PDCD6IP |
| IGF1R | IBD20 |
| IGFBP3 | IBD15 |
| IKBKB | IBD21 |
| IL2 | H3C9P |
| IL4R | IBD27 |
| IL5 | MIR1246 |
| IL7R | MIR1260A |
| AQP1 | MIR3194 |
| IL10RA | MIR4284 |
| IL10RB | LRRC3C |
| IL16 | CBR3-AS1 |
| TNFRSF9 | OCLN |
| INSRR | IBD11 |
| IRAK1 | FAM24B-CUZD1 |
| IRS1 | ERVK-10 |
| ABCC6 | ERVK-9 |
| ITGB2 | ERVK-21 |
| JAK3 | IL18BP |
| JUNB | PTPRU |
| AREG | ERVK-18 |
| PEAR1 | ERVK-25 |
| KDR | ERVK-24 |
| KIR2DS1 | ARPC2 |
| KIR3DL1 | LRPPRC |
| KIR3DL2 | OPTN |
| KRT18 | ABI2 |
| C11orf96 | SLC25A15 |
| TSPYL6 | POP7 |
| LAD1 | CDK9 |
| LAMA5 | ABCC4 |
| LAMC2 | CDKN1A |
| LAMP2 | IGSF6 |
| RPSA | CDKN1B |
| LCP1 | LOC102724197 |
| LEPR | CBSL |
| LGALS9 | CDKN2B |
| LIG4 | TNIP1 |
| LIMK1 | TRIM22 |
| LOXL1 | NDRG1 |
| LRP1 | RACK1 |
| LRP6 | FST |
| LINC01194 | NXF1 |
| CIMT | APPBP2 |
| MIRLET7B | FBLN5 |
| MIRLET7E | IPO8 |
| MIRLET7I | ERVK-19 |
| MIR106A | OLFM4 |
| MIR125B2 | SCGN |
| MIR129-2 | AHSA1 |
| MIR130B | USP16 |
| MIR140 | PDPN |
| MIR144 | POSTN |
| MIR152 | CES1 |
| MIR17 | AVIL |
| MIR185 | CORIN |
| MIR192 | CERS1 |
| MIR195 | IQGAP2 |
| MIR199B | CGA |
| MIR200C | HPSE |
| MIR206 | MALT1 |
| MIR212 | EHD1 |
| MIR216A | RAB32 |
| MIR219A1 | SDS |
| MIR22 | LILRB4 |
| MIR221 | STIN2-VNTR |
| MIR30D | ADAMTS13 |
| MIR31 | EMILIN1 |
| MIR34A | CD160 |
| MIR93 | WDHD1 |
| ARRB2 | WBP4 |
| MAPT | WIF1 |
| MBL2 | IRAK3 |
| MDK | AKAP13 |
| MDM2 | HPS5 |
| CIITA | CAVIN3 |
| NR3C2 | PACSIN2 |
| MOG | SCN11A |
| MRC1 | SLC52A3 |
| MIAT | CBX3 |
| POTEKP | CHRNA5 |
| ZFAS1 | DBA2 |
| MIR326 | TIRAP |
| MSD | MYSM1 |
| MSR1 | FCRL3 |
| PLF | ADCYAP1 |
| MTAP | LRG1 |
| CYTB | CLCN5 |
| MUSK | NXPE1 |
| MMUT | SLC15A4 |
| MVK | CCR3 |
| ATF3 | LTB4R |
| ATF4 | CMM |
| NDUFC2 | ZPBP2 |
| NFKB1 | CNGA1 |
| NM | COL1A1 |
| NNMT | SGPP2 |
| NPC1 | KLF6 |
| NPPB | SCLT1 |
| NTF4 | ADM |
| MIR422A | TAAR1 |
| MIR424 | COX8A |
| OATP1 | CLDN4 |
| OGG1 | CPOX |
| OPRK1 | CPT1A |
| OPRM1 | RNF183 |
| P2RX4 | ACTRT1 |
| P2RX7 | CRK |
| P2RY2 | MUC17 |
| DEFB104B | CRX |
| PAEP | NEK7 |
| NOX4 | CRYAB |
| SERPINB2 | CSF1R |
| IL20 | CSF2RA |
| PAPPA | LAYN |
| AK3 | CSF3R |
| PC | OR2AG1 |
| PDE11A | SLC2A14 |
| FOXP3 | NRG4 |
| SERPINA5 | CDAN1 |
| GAL | ZFP90 |
| MLXIPL | VCAN |
| RMDN1 | CARMIL2 |
| IRAK4 | IL34 |
| PCSK1 | CTF1 |
| MZB1 | CTNNB1 |
| TLR7 | COMMD1 |
| TLR8 | CTSG |
| SPTBN5 | CCDC80 |
| MS4A4A | CUX1 |
| ZC3HC1 | DAB2IP |
| PDGFRB | AGR3 |
| PECAM1 | CYP27B1 |
| RTEL1 | CD55 |
| STK26 | DBH |
| ASIC5 | DBP |
| SERPINE2 | DCC |
| SERPINI1 | IFNLR1 |
| PIK3R1 | DDX3X |
| PITX2 | DEFA6 |
| PKM | IBD8 |
| PLIN1 | ASXL1 |
| PLP1 | NLRP6 |
| ACP5 | DGUOK |
| PNN | DLD |
| NANS | AFP |
| POR | DMRT1 |
| POU2F1 | DNAH5 |
| TOMM7 | DNMT1 |
| DLL4 | DRD2 |
| EGLN1 | TSC22D3 |
| UGT1A1 | HBEGF |
| PPBP | DVL1 |
| ROPN1 | ECM1 |
| PPIA | S1PR1 |
| TRPM7 | EEF1A1 |
| RASIP1 | EGR1 |
| TMEM132A | AHSG |
| ANO1 | MLKL |
| RMDN3 | AIF1 |
| IMPACT | ELF1 |
| MIR449A | TMEM201 |
| PRH1 | ELK3 |
| PRH2 | ELK4 |
| KIF16B | TIGIT |
| SYBU | CTTN |
| PRKAR1A | ENO1 |
| VPS35 | SLC29A1 |
| PRKCA | EPHB1 |
| PRKCD | EPHB2 |
| RNF130 | EPHX1 |
| PRKD1 | EPHX2 |
| ACSS2 | STX2 |
| APOM | EPO |
| LRRC8A | SLC36A1 |
| MASP1 | ERBB2 |
| MMP26 | ERBB4 |
| B2M | ERF |
| SPHK2 | ERN1 |
| ANKS1B | ESRRB |
| PSMB9 | ETV5 |
| PDXP | ALCAM |
| RTN4 | ACSL1 |
| PTGDR | FASN |
| PTGER2 | LTO1 |
| MRS2 | FBN2 |
| PTGIR | FCER2 |
| MIR363 | FCGR1A |
| PTHLH | FCGR1B |
| MIR451A | FGD2 |
| MIR410 | FCGRT |
| MIR497 | JAZF1 |
| NDRG3 | FCN1 |
| NDRG2 | FCN2 |
| MIR503 | UNC5CL |
| NLN | FER |
| TAOK1 | FGB |
| SEMA6A | FGF2 |
| PTMA | FGFR1 |
| PTMAP4 | FGFR2 |
| RNF213 | ATF6 |
| PRX | DKK1 |
| HAMP | KIF21B |
| BARD1 | FOXO1 |
| NLRC4 | FOXO3 |
| IL21 | FLG |
| RAPSN | MPRIP |
| RARRES2 | FLNB |
| TRPV4 | ARL6IP1 |
| RENBP | MLC1 |
| ACTB | FLT3LG |
| RGS7 | FKBP15 |
| RNASE3 | TPSD1 |
| PROK2 | SF3B1 |
| RPS20 | SLC39A14 |
| RTN1 | CABIN1 |
| SORT1 | ZNF281 |
| S100A9 | SEC14L2 |
| S100A11 | PADI4 |
| SAA1 | AMACR |
| SAA2 | LY96 |
| TSPAN31 | PLA2G15 |
| CEACAM1 | TMEFF2 |
| BHMT | ALOX5 |
| CCL5 | TFIP11 |
| CCL19 | CLDN15 |
| CCL23 | ALPP |
| CX3CL1 | FTL |
| SDC1 | ACKR1 |
| SRR | TAC4 |
| NEUROG2 | PART1 |
| SELL | SUMF2 |
| CRLF2 | RNF19A |
| MOAP1 | CHMP2B |
| POTEM | REXO2 |
| MAP2K4 | AOC1 |
| SNHG6 | MAGI3 |
| ABCG8 | POLDIP2 |
| ITSN1 | AMCN |
| SHC1 | PHGDH |
| GORASP1 | GATA3 |
| SHH | FGF20 |
| SHMT1 | KAT2A |
| SMURF2 | IL36RN |
| NMNAT1 | GDF1 |
| MARCHF7 | GGT1 |
| AGXT2 | RNU1-1 |
| PINK1 | GHSR |
| ACD | DNAI1 |
| NBEAL1 | SND1 |
| MARCKSL1 | B3GAT1 |
| WNK1 | CPAMD8 |
| WNK3 | IL36A |
| SLC6A4 | IL17B |
| SLC6A11 | VENTX |
| SLC6A13 | ADAMDEC1 |
| SLC12A3 | RABGEF1 |
| SLC19A1 | MCAT |
| SLC20A2 | GLI3 |
| SLCO1A2 | GLP1R |
| CYP4F12 | GNAI2 |
| SMN1 | GNAO1 |
| SMN2 | IFNL2 |
| SNCA | GPR4 |
| SNRNP70 | CCDC88B |
| MIR544A | PGP |
| MIR487B | GSDMA |
| SOD2 | CXCL17 |
| SOD3 | ANGPT2 |
| BNIP3L | GPER1 |
| SOX2 | SLC9C1 |
| SREBF2 | KIF9-AS1 |
| SRF | PPP1R42 |
| BRCA2 | GPT |
| SSTR4 | TRIB2 |
| STAR | GRN |
| STAT4 | MCTS1 |
| STAT5A | C1GALT1C1 |
| STAT5B | PYCARD |
| SCARNA6 | CLEC2D |
| STIM1 | UHRF1 |
| BRS3 | GTF2F1 |
| ABCC8 | USP25 |
| SYT1 | GUCA2A |
| TAC1 | GUCA2B |
| ADAM17 | SNX10 |
| BTF3P11 | GUSB |
| MIR532 | CERS2 |
| MIR605 | MDFIC |
| MIR608 | PILRA |
| MIR618 | GZMB |
| MIR638 | GZMM |
| ZEB1 | ANXA1 |
| TCN2 | ANXA2 |
| TEK | HADHA |
| TERC | HDAC2 |
| TERT | HFE |
| TFDP1 | HGD |
| TFPI | CD209 |
| TGM2 | HLA-A |
| THBS1 | HLA-DOA |
| TIMP1 | HLA-DPA1 |
| TLR3 | HLA-DQB1 |
| ACTG1 | HLA-DQB2 |
| SERPING1 | HLA-DRA |
| TLR5 | HLA-DRB5 |
| TM7SF2 | HLA-G |
| CLDN5 | SLC29A2 |
| TNFAIP3 | HPRT1 |
| TNFRSF1A | AIRE |
| TNNI3 | ERAS |
| TRC-GCA24-1 | BIRC2 |
| TRAF3 | HSPA1L |
| ACTG2 | HSPA2 |
| TRH | HSPB1 |
| TRPC6 | HSPB2 |
| TRPM2 | APOA1 |
| TRPS1 | TNC |
| C5 | IAPP |
| TTR | ICA1 |
| UBE2L3 | HCAR2 |
| UCHL1 | ICAM3 |
| UGCG | FAM92B |
| UGT2B4 | SLC6A19 |
| UMOD | STING1 |
| VPS51 | RSPO2 |
| USF1 | IDH2 |
| UTRN | IDUA |
| VASP | IFI16 |
| VDR | AQP8 |
| VEGFB | IFN1@ |
| VIP | IFNAR1 |
| VSNL1 | IFNB1 |
| VTN | APOE |
| WRN | IGF1R |
| CACNA1C | IGFBP4 |
| ZNF208 | DNAAF3 |
| MANF | KLK3 |
| REEP5 | FAS |
| MUL1 | IKBKB |
| ADIPOR2 | IL3 |
| TNFAIP8L2 | IL5 |
| DHX40 | CXCR1 |
| TFPI2 | IL12A |
| EHMT1 | IL12RB1 |
| ARHGEF5 | AQP3 |
| MAP9 | IL15RA |
| ERMP1 | IMPDH1 |
| WLS | AQP5 |
| NAA25 | ING1 |
| CALM1 | IREB2 |
| ZC3H12A | IRF1 |
| FLAD1 | AQP9 |
| KCNIP4 | ITGA2B |
| CALR | ITGB1 |
| CALU | ITGB2 |
| AKAP1 | ITGB3 |
| RNF146 | ITIH1 |
| QTRT1 | ITIH2 |
| BRAP | ITIH3 |
| CASP1 | JAK1 |
| PLVAP | AREG |
| BCO2 | TEPP |
| TSPAN10 | KCNN4 |
| CASP7 | KCNQ2 |
| CASP8 | KIR2DL2 |
| MINDY4 | KIR2DS5 |
| KLF11 | KIR3DL2 |
| NTNG2 | KIR3DS1 |
| KDM2B | KLK1 |
| ACCS | KNG1 |
| CAT | KRT1 |
| PSRC1 | KRT7 |
| GPR65 | IL31 |
| IL17RC | KRT18 |
| ORAI1 | CCL4L1 |
| APOL1 | L1CAM |
| DOCK7 | LGALS1 |
| PIAS1 | LGALS2 |
| DENR | LGALS3BP |
| MADD | LIF |
| TNFSF11 | LMNA |
| PLPP3 | IL17REL |
| PDE5A | LPL |
| TNKS | LINC01194 |
| ABCC3 | LTA4H |
| TNFSF14 | LTB |
| TNFSF10 | SH2D1A |
| TNFRSF11A | MIR106A |
| IL18RAP | MIR10A |
| IL18R1 | MIR124-1 |
| NR1I2 | MIR125A |
| SQSTM1 | MIR141 |
| OTULIN | MIR142 |
| USP14 | MIR148A |
| LPAR2 | MIR149 |
| IL1RL1 | MIR18A |
| XPR1 | MIR185 |
| METTL18 | MIR192 |
| NOG | MIR196A2 |
| MSC | MIR204 |
| SPECC1 | MIR22 |
| DNER | MIR29A |
| ASIC3 | MIR301A |
| CD163 | MIR320A |
| ZFYVE9 | MIR34C |
| CD28 | MIR7-1 |
| MUC16 | MIR7-2 |
| FADS2 | MIR7-3 |
| HOMER2 | SMAD4 |
| HOMER1 | ARRB2 |
| FHL5 | SMAD5 |
| CD34 | STS |
| CD36 | MAN2A1 |
| ADAMTS4 | DEFB103A |
| ADAMTS3 | PIM3 |
| GTPBP1 | ARSF |
| SNCAIP | MCC |
| PDE4DIP | MDK |
| CD74 | MGAT1 |
| HDAC4 | MGAT3 |
| ACYP2 | MGMT |
| XYLB | CIITA |
| SLC23A2 | CD99 |
| CCS | MIP |
| MT-TL1 | MLN |
| GP1BA | ACHE |
| ACSL4 | ASCL2 |
| MT-ND1 | FOXO4 |
| ADA2 | MMP8 |
| HTRA1 | MMP10 |
| MT-ND6 | MMP12 |
| MT-CO1 | MMP19 |
| MT-CO2 | MNAT1 |
| KAT6B | MPG |
| DUSP29 | MRC1 |
| GUCY1A1 | MIR148B |
| MT-CO3 | MIR346 |
| PSEN1 | MIR374A |
| PROZ | MSD |
| LMNA | ERVK-7 |
| HMGCR | COX1 |
| F10 | MTR |
| SCN5A | RNR1 |
| GSR | MTX1 |
| ENPP1 | MUC3A |
| LOC102723566 | MUSK |
| HEY2 | MYC |
| MAP2 | MYH10 |
| SMAD4 | ATF4 |
| PF4 | NCAM1 |
| PIK3C2A | NCF2 |
| INS | NEDD8 |
| SMAD2 | NELL1 |
| CYCS | NFKBIA |
| ADORA1 | NOS1 |
| MBP | NPY |
| MPL | NPR1 |
| CREB1 | NPR3 |
| HBB | NPY2R |
| EPOR | NTRK1 |
| MB | NTS |
| TET2 | NTSR1 |
| MT-CYB | MIR425 |
| MROS | ODC1 |
| GATA4 | OPRM1 |
| SOD1 | P2RX5 |
| RNF213-AS1 | P4HB |
| SULT1A3 | PAFAH1B1 |
| AKT1 | IL20 |
| TBXA2R | IBD4 |
| AOC3 | IL21R |
| AIF1 | PAPPA |
| CCL3 | F11R |
| THPO | PC |
| BMP7 | PCBD1 |
| ANXA5 | SOST |
| GDNF | EXOSC1 |
| HBA1 | ADIPOR1 |
| ENTPD1 | NT5C3A |
| ACTC1 | TLR7 |
| TNNT2 | PDC |
| RPL36A-HNRNPH2 | TNFRSF12A |
| ACTA2-AS1 | CRNKL1 |
| ADORA3 | POP5 |
| DARS2 | COMMD10 |
| DCX | ISYNA1 |
| MYH7 | SCLY |
| SMARCAL1 | PDGFB |
| BACE1 | GDE1 |
| SERPIND1 | KLF13 |
| PRNP | PDYN |
| HHT4 | PDZK1 |
| BRCC3 | PECAM1 |
| CCM2 | PEPD |
| CASP9 | PF4 |
| MYH6 | CFP |
| NDE1 | PGA5 |
| PMM2 | PGGT1B |
| POLG | SLC25A3 |
| LIPC | SERPINE2 |
| ZMPSTE24 | PIGA |
| GATA6 | PIK3C3 |
| PKD1 | PIK3CA |
| MYBPC3 | PIK3CB |
| BGN | PIK3CG |
| CALCA | PLAT |
| CP | PLEK |
| RBFOX3 | DUOX1 |
| KRAS | EIM |
| AMPD1 | TREM2 |
| TH | TERF2IP |
| MYMY3 | PON3 |
| MYMY1 | TOLLIP |
| KCNJ5 | KRT20 |
| NF1 | SMOX |
| NCF1 | POU2F1 |
| LOC113939944 | RNF186 |
| TGFBR3 | EGLN1 |
| KRIT1 | PPARA |
| LOC107133510 | UGT1A1 |
| LOC106099062 | PPIA |
| FLVCR2 | AFTPH |
| RGS5 | MS4A12 |
| TBX20 | MARCHF1 |
| GNAQ | AVP |
| SLC1A3 | ANKZF1 |
| CAPN3 | PBRM1 |
| KLKB1 | QRSL1 |
| FLT1 | KIR2DL5B |
| MTX2 | BATF3 |
| MEN1 | PRF1 |
| AAT1 | PRKAA1 |
| RPS27A | AMBRA1 |
| FLNA | PRKAA2 |
| DPM3 | PRKAB1 |
| GJA5 | ATF7IP |
| TF | IFT122 |
| MMACHC | SELENOS |
| CTF1 | USE1 |
| DES | GSDMB |
| CTSA | DEFB103B |
| TCF7L2 | ACSS2 |
| SRFBP1 | MAPK3 |
| AAT2 | NSFL1C |
| ACAN | EIF2AK2 |
| PDGFRA | PRNP |
| INSL6 | PSPN |
| LOC110006319 | PARD3 |
| PRL | HTRA1 |
| CITED2 | PSAP |
| COL4A5 | ASAH2 |
| CUX2 | TRIM39 |
| JAG1 | PSG5 |
| COL2A1 | PSMA7 |
| PKD2 | DUSP22 |
| ATP1B1 | FAM20C |
| MYMY4 | IBD7 |
| ITIH4 | PSMD4 |
| SLC34A1 | RTN4 |
| FKRP | PSMD10 |
| FLNC | PTAFR |
| XDH | PTCH1 |
| CPS1 | KIR2DL5A |
| PTPN11 | PTGDS |
| CCND1 | SENP7 |
| GHRL | MIR146B |
| CELA2A | MIR495 |
| GNB1 | MIR505 |
| ATP1A2 | TAOK1 |
| PVALB | MRTFA |
| GBA | PRX |
| C3 | ZNF410 |
| HYT1 | MUC3B |
| TTN | PTPRC |
| CS | PTPRH |
| HYT8 | PTPRS |
| HYT7 | PTPRZ1 |
| HYT6 | CXCL16 |
| HYT5 | SCAF1 |
| HYT4 | PRM3 |
| HYT3 | MOK |
| HYT2 | RAP1A |
| MC4R | RAPSN |
| MIR9-1 | RBM3 |
| POU4F1 | NPS |
| NSD1 | REL |
| SCNN1A | RNASE2 |
| TLL1 | SLC25A19 |
| VHL | RNF5 |
| BGLAP | RNU1-4 |
| MYL4 | PROK2 |
| HSPA5 | TNFRSF17 |
| CHAT | ROS1 |
| BRAF | RPLP0 |
| PPOX | RPLP2 |
| PCNT | SCART1 |
| FN1 | ERVK-8 |
| FAS | BDKRB2 |
| POMC | S100A10 |
| OTC | SAA2 |
| SERPINA1 | CFB |
| IL1R1 | SAA3P |
| SLC1A1 | CLEC11A |
| TBX5 | CCL4 |
| VCP | CCL7 |
| MYH9 | CCL14 |
| CASP2 | CCL15 |
| COX5B | CCL17 |
| FABP2 | CCL22 |
| MGP | SELP |
| OPN4 | SLC28A3 |
| COG2 | SLC39A8 |
| MTERF1 | DIO3OS |
| ITGAM | SRSF1 |
| NDUFS4 | CRNDE |
| DPAGT1 | DCLRE1C |
| PHACTR1 | SGCG |
| ADA | SHBG |
| KCNE2 | SI |
| PCCA | FNDC4 |
| EPRS1 | BMP1 |
| BCL2L1 | BCL11B |
| EGF | BMP2 |
| ANGPTL6 | NDRG4 |
| ARRDC4 | RAPH1 |
| COL1A1 | SLC2A3 |
| ABCC9 | SLC2A5 |
| ADRB3 | SLC3A2 |
| ELANE | UBE2Z |
| CLCN1 | SLC6A2 |
| CDH5 | SLC6A4 |
| RYR1 | NCF1 |
| MAN2B1 | SFTPA1 |
| KCNA5 | GGTLC5P |
| PTPN22 | SLC6A9 |
| ITGB1 | SLC7A2 |
| NTS | SLC9A1 |
| BMP6 | SLC9A2 |
| ERCC6 | BMP7 |
| KIF20A | SLC16A1 |
| CKB | SLCO2A1 |
| CORIN | SLC22A1 |
| KCNJ2 | SMS |
| IL5RA | SUMO3 |
| ASS1 | SUMO2 |
| PGF | SNCA |
| IRF5 | H3P37 |
| GGT1 | SOD3 |
| MFN2 | BRCA1 |
| MIR29A | BRAF |
| SAMHD1 | TRIM21 |
| LOC110806262 | RO60 |
| TIMP3 | SST |
| PTH | ST2 |
| MGR6 | STAT5A |
| MGR5 | STAT5B |
| XIAP | SULT1E1 |
| TSPAN2 | SYK |
| CD68 | TACR2 |
| GDF2 | TAL1 |
| HTR3A | TBP |
| CLU | TCEA1 |
| SDHB | MIR595 |
| ASTN2 | BTG1 |
| TARDBP | TCF21 |
| F13B | TCOF1 |
| SLC2A1 | PRDX2 |
| CPT2 | TEK |
| ODC1 | TERF2 |
| ASIC1 | TERT |
| MEFV | TFF2 |
| GH1 | TGFA |
| NRP1 | TGFB2 |
| TNFRSF1B | TGFBR2 |
| SCN2A | TSPO |
| LIPA | TIAL1 |
| KMO | TJP1 |
| SRC | TP73 |
| TFAM | TPH1 |
| MGR12 | C3 |
| MGR8 | TRAF1 |
| MGR3 | TRAF2 |
| MGR2 | TRAF3 |
| MGR11 | TRAF5 |
| MGR10 | C4B |
| MGR1 | TST |
| MYD88 | MUC5B |
| THSD1 | GGTLC3 |
| MT-ND2 | TUFM |
| CCN2 | GGT2 |
| PNP | TNFSF4 |
| WDR45 | CCR2 |
| ITLN1 | TNFRSF4 |
| CD63 | TXK |
| CNR1 | GGTLC4P |
| NPPA-AS1 | TYROBP |
| KITLG | TYRP1 |
| CCR6 | UBC |
| DRD1 | UBE2I |
| LOC106627981 | UCP2 |
| SCNN1B | VPS51 |
| FGF23 | VCL |
| PPARGC1A | VIM |
| PLOD1 | TRPV1 |
| KCNJ11 | WARS1 |
| IGF2BP2 | WNT1 |
| ATFB5 | YWHAE |
| UQCRFS1 | ZFP36 |
| KIT | CA2 |
| UCP3 | ZNF133 |
| COX4I1 | SLC25A20 |
| PON3 | CAD |
| KCNMA1 | ATG9A |
| ADAMTSL1 | CARD14 |
| SCN1A | LST1 |
| TYMP | CALCA |
| GYS1 | AIMP2 |
| PCCB | STEAP4 |
| HSD11B2 | MORC4 |
| IL3 | SNIP1 |
| BMP2 | GSDMD |
| BAD | CALCR |
| LOC106029312 | ELOVL7 |
| SCGB1A1 | MINDY3 |
| WARS2 | CCDC6 |
| TFB1M | FLAD1 |
| NAGS | PPP1R2C |
| CMA1 | PNPLA3 |
| HSPA8 | FOSL1 |
| S100A1 | NPL |
| DNAH8 | CALR |
| PRKN | GPR68 |
| CYP17A1 | CAMK4 |
| KYNU | SLC7A5 |
| FLNC-AS1 | SGPP1 |
| ADAMTS1 | CDR3 |
| DRD2 | MAP1LC3B |
| CYP11B1 | VMP1 |
| DMD | MIA |
| SLC2A4 | NRIP1 |
| FMR1 | BAS |
| NUP155 | CAST |
| JPH3 | AXIN1 |
| IGFBP1 | EOMES |
| FOXP2 | ARHGAP24 |
| NT5E | BCL2L12 |
| ALG13 | EIF2A |
| CCDC88C | PLA2G6 |
| SLC6A3 | CASP7 |
| GC | MAGT1 |
| FABP3 | STK24 |
| INSR | CARD11 |
| CCR3 | DHX16 |
| PRKAG2 | CASR |
| MTTP | TRIM63 |
| MIR196A2 | ACCS |
| ITGAL | HAVCR2 |
| MME | RSPO3 |
| COL1A2 | PPM1D |
| GHR | HAT1 |
| C4B | SHANK3 |
| FTO | MYLK2 |
| DDAH1 | PIR |
| NR4A2 | TSLP |
| DNAH5 | PIAS1 |
| PDGFB | USO1 |
| PTK2B | CBFA2T3 |
| GAA | RNASET2 |
| ROCK1 | HYAL2 |
| MIR199A1 | S1PR4 |
| DLG4 | TNK1 |
| CNTF | CBR1 |
| SHBG | CBS |
| MUC1 | TNFRSF14 |
| C4A | CES2 |
| GATA5 | KYAT1 |
| SLC8A1 | GMPS |
| FURIN | SOCS2 |
| FHOD3 | CCN4 |
| CBL | KAT2B |
| ADORA2A | APLN |
| SLCO1C1 | PER3 |
| NEFH | PER2 |
| TWIST1 | MTMR3 |
| BCHE | PHOX2B |
| SOST | CCNE1 |
| HTT | ATG16L2 |
| DCN | HSPB3 |
| IRS2 | PGLYRP1 |
| IL2RA | NOL3 |
| SUOX | NAT1 |
| KIF6 | SOCS3 |
| GCK | PSTPIP1 |
| GAPDH | CLDN8 |
| NOTCH4 | LATS1 |
| PDCD1 | NLRP12 |
| CSTB | CD3G |
| CTSB | MYLK3 |
| CYP1B1 | GGTLC1 |
| IAPP | MTA2 |
| PSEN2 | MAGI1 |
| LEPQTL1 | REEP6 |
| SPTB | GPR55 |
| AIFM1 | S1PR2 |
| BDKRB2 | CD19 |
| MS4A2 | PGAP3 |
| RBPJ | CD163 |
| NOTCH2 | SLIT2 |
| SLC6A2 | SLC9A3R1 |
| CFLAR | KIF3B |
| CHGA | PLAA |
| APOA4 | COX5A |
| DLL1 | CD101 |
| PTPN1 | GRAP2 |
| FNDC5 | MUC16 |
| HNF1B | CD80 |
| TIE1 | ORMDL3 |
| VIPR2 | CD86 |
| PIGQ | NTN1 |
| KIAA0319L | TNFRSF8 |
| CLCN6 | TNFSF8 |
| SCN8A | GSTO1 |
| TCF7 | EIF2AK3 |
| GRIA1 | ROCK2 |
| CYBB | CD36 |
| PTGDS | CD38 |
| CAMK2G | PTGES |
| H2AC18 | CXCL14 |
| RYR2 | CCL4L2 |
| JAG2 | PDIA4 |
| DLL3 | CD47 |
| SLC2A3 | SOCS5 |
| MYC | CD59 |
| EPHB4 | CD70 |
| ATP1A3 | HDAC4 |
| IDO1 | ZEB2 |
| CD79A | ZBTB24 |
| SRRT | FCHSD2 |
| HEY1 | WDR1 |
| PMM1 | USP15 |
| SOCS3 | NR1H4 |
| AKR1B1 | CDH1 |
| BAX | GAA |
| TAGLN | PKHD1 |
| STX1A | PKD1 |
| NOD2 | GBA |
| TRIM2 | APP |
| DBH | RET |
| GRM5 | PSEN1 |
| LTB | IBD9 |
| STIN2-VNTR | IBD16 |
| ADH1C | IBD18 |
| IFNA1 | IBD12 |
| IFIH1 | IBD22 |
| SIL1 | IBD23 |
| AHCY | IBD24 |
| MAOA | IBD26 |
| GRM7 | NPC1 |
| SCNN1G | IAH1 |
| MIR124-1 | PKD2 |
| RUNX2 | SQSTM1 |
| MAOB | SMPD1 |
| CSF1R | VCP |
| CELF2 | PRKCQ |
| DENND11 | MAPT |
| ST2 | MFN2 |
| KCNMB1 | ATP7B |
| GAS6 | GJB1 |
| HSD11B1 | EDNRB |
| APEX1 | CYBA |
| LBP | NEFL |
| PIGA | FLNA |
| CALCRL | RYR1 |
| AKT2 | GBE1 |
| CHRNA7 | G6PC1 |
| HRAS | CCL3 |
| MIR125B1 | SOX10 |
| TNXB | TTR |
| LCAT | AGL |
| MMP13 | GARS1 |
| STK11 | ABCA1 |
| CDH11 | SERPINA1 |
| KCNN4 | SLC37A4 |
| ERAP1 | GLA |
| CD177 | PSEN2 |
| SP1 | GFAP |
| BMPR1A | INS |
| IDH1 | SLC17A5 |
| IL12A | EDN3 |
| CSF1 | FBN1 |
| EIF2AK2 | HEXA |
| SPG7 | KIT |
| WNT2B | DNM2 |
| ITPR3 | IGHMBP2 |
| STN1 | NAGLU |
| MTHFD1 | AR |
| ABCB7 | POLG |
| MIR24-1 | MT-ND1 |
| CCR1 | HBB |
| MEF2D | EGR2 |
| ALPL | PYGM |
| DEFA1 | PLP1 |
| YY1AP1 | PINK1 |
| NTRK1 | NPC2 |
| NTRK2 | ATP7A |
| HSPD1 | CD4 |
| ROS1 | COL2A1 |
| DNAH11 | ATM |
| GSN | MIR34A |
| COL6A3 | HNF1B |
| VAMP8 | PDGFRA |
| PALLD | MIR17 |
| LOC114827827 | TNFRSF11A |
| IRF1 | HTT |
| SNX19 | ABCD1 |
| MYH15 | TSC2 |
| ICAM4 | BRCA2 |
| GSTO2 | F8 |
| CRH | CCR1 |
| SERPINA12 | ALK |
| MYOCD | COL4A1 |
| NPR1 | KIF1B |
| IL1F10 | MTMR2 |
| SYP | MME |
| CRELD1 | ARPC1B |
| FIP1L1 | ELN |
| BLK | UCHL1 |
| LEMD3 | LAMP2 |
| LRP5 | APOB |
| APOA2 | SERPINA3 |
| KLC1 | IBD19 |
|  | ICOSLG |
|  | ACTA2 |
|  | IBD25 |
|  | UMOD |
|  | CEP290 |
|  | C4A |
|  | SREBF1 |
|  | LRP5 |
|  | MSH2 |
|  | TTN |
|  | RNF213 |
|  | JAG1 |
|  | LTF |
|  | MIR145 |
|  | MORC2 |
|  | TGFBR1 |
|  | CFH |
|  | PLA2G2A |
|  | NF1 |
|  | VHL |
|  | JUP |
|  | CLN3 |
|  | FGFR3 |
|  | CYBC1 |
|  | SAG |
|  | F9 |
|  | COMT |
|  | COL3A1 |
|  | PRKAG2 |
|  | SERPINC1 |
|  | HEXB |
|  | ECE1 |
|  | LDLR |
|  | LIPA |
|  | CP |
|  | MSH6 |
|  | PROM1 |
|  | F13A1 |
|  | IL7R |
|  | MT-ATP6 |
|  | PHKA2 |
|  | ENG |
|  | ZAP70 |
|  | DSP |
|  | GHRL |
|  | FASLG |
|  | ABCB4 |
|  | SCN5A |
|  | THBD |
|  | PFKM |
|  | PMS2 |
|  | C9orf72 |
|  | CD79A |
|  | MIR143 |
|  | SMN1 |
|  | ALG9 |
|  | B2M |
|  | ITCH |
|  | ADA2 |
|  | EDNRA |
|  | GATA4 |
|  | SMAD6 |
|  | MT-CO1 |
|  | SFTPC |
|  | EPCAM |
|  | GNAS |
|  | NKX2-5 |
|  | CTSD |
|  | CPT2 |
|  | CD8A |
|  | CC2D2A |
|  | ERBB3 |
|  | MYH11 |
|  | BGLAP |
|  | HRAS |
|  | COL17A1 |
|  | PLA2G7 |
|  | MT-CYB |
|  | GYS1 |
|  | MIR140 |
|  | KRT5 |
|  | AGTR1 |
|  | HNRNPA1 |
|  | ADAM10 |
|  | NRTN |
|  | SMIM35 |
|  | ATP2A2 |
|  | TREX1 |
|  | MT-TL1 |
|  | MVK |
|  | TPM3 |
|  | JUN |
|  | LCAT |
|  | MIR210 |
|  | CYP27A1 |
|  | TBX5 |
|  | DES |
|  | SMAD2 |
|  | ACTC1 |
|  | ENPP1 |
|  | CST3 |
|  | SOCS1 |
|  | CXCL2 |
|  | GM2A |
|  | FOS |
|  | CAV3 |
|  | BMPR2 |
|  | MECP2 |
|  | TBK1 |
|  | PRKAR1A |
|  | NCF4-AS1 |
|  | CAV1 |
|  | CELIAC5 |
|  | ACTA1 |
|  | STK11 |
|  | TSC1 |
|  | GP1BA |
|  | MEN1 |
|  | CELIAC6 |
|  | TH |
|  | CELIAC10 |
|  | CELIAC11 |
|  | CELIAC12 |
|  | CELIAC13 |
|  | CELIAC7 |
|  | CELIAC8 |
|  | CELIAC9 |
|  | IL1RAPL2 |
|  | SP110 |
|  | FUS |
|  | SDHD |
|  | MIR221 |
|  | NRAS |
|  | MIR29B1 |
|  | CHEK2 |
|  | IRAK1 |
|  | CHUK |
|  | CD27 |
|  | SRC |
|  | FCGR2B |
|  | CHAT |
|  | MIR106B |
|  | PI4KA |
|  | CAPN3 |
|  | COL1A2 |
|  | CXCL1 |
|  | ADRB2 |
|  | CXCL9 |
|  | MT-ND5 |
|  | COL7A1 |
|  | GIGYF2 |
|  | PDGFRB |
|  | HNRNPA2B1 |
|  | DYNC2H1 |
|  | MIR203A |
|  | LMNB1 |
|  | MTM1 |
|  | CDH23 |
|  | RRM2B |
|  | ACP5 |
|  | G6PD |
|  | VPS35 |
|  | ABCB11 |
|  | MT-ND4 |
|  | LOX |
|  | NPHP1 |
|  | GUCY2D |
|  | TMEM67 |
|  | RAG1 |
|  | MET |
|  | PIK3R1 |
|  | SCN4A |
|  | KITLG |
|  | INPP5E |
|  | TSHR |
|  | GATA6 |
|  | RHO |
|  | WT1 |
|  | DNASE1 |
|  | TGM2 |
|  | VPS13C |
|  | MAP2K1 |
|  | CDSN |
|  | BAX |
|  | EP300 |
|  | KCNQ1 |
|  | CRB1 |
|  | ATRX |
|  | CLCN1 |
|  | GSTP1 |
|  | IFT140 |
|  | GATA1 |
|  | AIFM1 |
|  | MT-CO2 |
|  | MT-ND6 |
|  | RANBP2 |
|  | TF |
|  | CTSB |
|  | DNAJC6 |
|  | MIR144 |
|  | SBF1 |
|  | CACNA1A |
|  | SDHB |
|  | MKS1 |
|  | CR2 |
|  | SGSH |
|  | HTRA2 |
|  | MUTYH |
|  | PAX2 |
|  | SCN9A |
|  | LDHA |
|  | PELATON |
|  | ALMS1 |
|  | ACVRL1 |
|  | MBTPS2 |
|  | ACTG2 |
|  | AHI1 |
|  | NPPB |
|  | BCL10 |
|  | SOD2 |
|  | SHH |
|  | MITF |
|  | MYH9 |
|  | BMP6 |
|  | MIR222 |
|  | ZFYVE26 |
|  | TGFB3 |
|  | NOTCH3 |
|  | RBP4 |
|  | RUNX1 |
|  | MBP |
|  | HTR2A |
|  | FMR1 |
|  | ERCC6 |
|  | SLC34A1 |
|  | GJC2 |
|  | SOS1 |
|  | COL11A1 |
|  | HMGCR |
|  | CREB1 |
|  | STXBP2 |
|  | CREBBP |
|  | FGF23 |
|  | BTK |
|  | CLU |
|  | DCTN1 |
|  | CD34 |
|  | USH2A |
|  | CHD7 |
|  | GNB3 |
|  | PHKB |
|  | TNNI3 |
|  | CR1 |
|  | COL5A1 |
|  | ABL1 |
|  | SOX9 |
|  | SMO |
|  | IFT172 |
|  | WFS1 |
|  | SYP |
|  | SMN2 |
|  | PTH |
|  | LOC106029312 |
|  | BBS1 |
|  | TTN-AS1 |
|  | NPHP4 |
|  | TPO |
|  | CYP19A1 |
|  | MIR20A |
|  | EMD |
|  | EPX |
|  | STX3 |
|  | MYBPC3 |
|  | GYG1 |
|  | RYR2 |
|  | RAF1 |
|  | WDR19 |
|  | TTC21B |
|  | RTEL1 |
|  | GREM1 |
|  | ENO2 |
|  | SEMA3C |
|  | MYO5B |
|  | TYR |
|  | CYP2C9 |
|  | FBXO7 |
|  | PLEC |
|  | RBCK1 |
|  | PEX7 |
|  | PSMB9 |
|  | ERCC2 |
|  | ADH1C |
|  | FLT4 |
|  | IKZF1 |
|  | PYY |
|  | BACE1 |
|  | SCT |
|  | SLC2A1 |
|  | APOH |
|  | NBN |
|  | LBR |
|  | NPHS1 |
|  | PPARGC1A |
|  | H2AC18 |
|  | MIR182 |
|  | F12 |
|  | NR1H2 |
|  | CLTC |
|  | SMARCA4 |
|  | SFTPB |
|  | PDE6B |
|  | PRKCD |
|  | LRP1 |
|  | PSMB8 |
|  | MIR127 |
|  | BCS1L |
|  | THPO |
|  | NFAT5 |
|  | NPPA |
|  | FKRP |
|  | EIF4G1 |
|  | MT-CO3 |
|  | ATP8B1 |
|  | STX11 |
|  | LMX1B |
|  | PALB2 |
|  | LCK |
|  | INSR |
|  | ABCC6 |
|  | THBS1 |
|  | ARSB |
|  | POGLUT1 |
|  | SLC6A3 |
|  | IL12RB2 |
|  | RPS27A |
|  | MIR328 |
|  | SPG7 |
|  | C5 |
|  | CETP |
|  | GSK3B |
|  | SDHA |
|  | MIR200A |
|  | KDR |
|  | MIR29C |
|  | KCNH2 |
|  | PAX6 |
|  | SLPI |
|  | KL |
|  | PSORS1C1 |
|  | TYMP |
|  | MPV17 |
|  | NR3C2 |
|  | FLT1 |
|  | SEMA3D |
|  | BCL2L1 |
|  | IL21-AS1 |
|  | CRYAA |
|  | MDM2 |
|  | DCDC2 |
|  | MIR342 |
|  | SPAST |
|  | SPINK1 |
|  | CCL18 |
|  | TBX1 |
|  | KAT6B |
|  | WIPF1 |
|  | PMM2 |
|  | BCHE |
|  | IRAK4 |
|  | CD247 |
|  | C1S |
|  | MIR214 |
|  | PRKG1 |
|  | PANK2 |
|  | GDF15 |
|  | F10 |
|  | ASAH1 |
|  | MIR30A |
|  | SAMHD1 |
|  | MIR200C |
|  | POFUT1 |
|  | BMP4 |
|  | PRL |
|  | RB1 |
|  | KLRC4 |
|  | SLC12A3 |
|  | CYP1B1 |
|  | AIP |
|  | F7 |
|  | MIR27A |
|  | H19 |
|  | CLCNKB |
|  | SERPINF1 |
|  | NFKBIL1 |
|  | CEACAM3 |
|  | CSTB |
|  | IFNA2 |
|  | NOTCH2 |
|  | CYCS |
|  | MRE11 |
|  | PGR |
|  | COL5A2 |
|  | SEMA4D |
|  | RUNX2 |
|  | COL9A2 |
|  | NCSTN |
|  | HAVCR1 |
|  | ALPL |
