# Supplementary material for: Uncovering the Mechanism of the Xingnaojing Injection against Ischemic Stroke Using a Combined Network Pharmacology Approach and Gut Microbiota Analysis
Source: Evid Based Complement Alternat Med. 2022 May 20;2022:5886698. doi: 10.1155/2022/5886698 (PMC9142292; doi:10.1155/2022/5886698)
Supplement: Supplementary Materials — Supplementary Table S1: active ingredients of XNJ identified by UHPLC-MS. Supplementary Table S2: 507 compound-related targets. Supplementary Table S3: 1667 IS-related targets and 2074 IBD-related targets. Supplementary Table S4: 210 shared targets were identified as potential therapeutic targets of XNJ against IS. . [file 5886698.f1.zip › 5886698.f1/S4 shared targets.docx]

| IS shared targets | IBD shared targets |
| --- | --- |
| ABCB1 | ABCB1 |
| ABCC1 | ABCG2 |
| ABCC9 | ACE |
| ABCG2 | ACHE |
| ACE | ACP1 |
| ACE2 | ADA |
| ACHE | ADAM10 |
| ADA | ADAM17 |
| ADAM17 | ADORA2B |
| ADORA1 | ADORA3 |
| ADORA2A | AGTR1 |
| ADORA3 | AKT1 |
| ADRA1A | ALB |
| ADRA2A | ALOX5 |
| ADRA2B | ALPL |
| ADRA2C | APP |
| ADRB1 | AR |
| AGTR1 | BACE1 |
| AHCY | BCHE |
| AKR1B1 | BCL2 |
| AKT1 | BCL2L1 |
| ALB | BMP1 |
| ALDH2 | BRAF |
| ALOX5 | BRD2 |
| ALOX5AP | CA2 |
| ALPL | CCND1 |
| AMPD1 | CCR5 |
| ANPEP | CCR9 |
| AOC3 | CD38 |
| APP | CES1 |
| AR | CES2 |
| BACE1 | CHUK |
| BCHE | CNR1 |
| BCL2 | CNR2 |
| BCL2L1 | COMT |
| BRAF | CPT1A |
| CCND1 | CREBBP |
| CCR5 | CTNNB1 |
| CDK5 | CTSB |
| CHRNA4 | CX3CR1 |
| CNR1 | CXCR2 |
| COMT | CYP19A1 |
| CTNNB1 | CYP2C9 |
| CTSA | CYP2D6 |
| CTSB | DNMT1 |
| CX3CR1 | DPP4 |
| CYP11B1 | DRD2 |
| CYP11B2 | ECE1 |
| CYP17A1 | EDNRA |
| CYP2C19 | EDNRB |
| CYP2C9 | EGFR |
| CYP2D6 | ELANE |
| DPP4 | EP300 |
| DRD1 | EPAS1 |
| DRD2 | EPHX1 |
| ECE1 | EPHX2 |
| EDNRA | ERN1 |
| EDNRB | ESR1 |
| ELANE | ESR2 |
| EPAS1 | F2 |
| EPHX2 | F2R |
| ESR1 | FGF2 |
| ESR2 | FLT1 |
| F2 | FLT4 |
| F2R | FOLH1 |
| FABP3 | G6PD |
| FABP4 | GAA |
| FGF1 | GAPDH |
| FGF2 | GBA |
| FLT1 | GLB1 |
| FOLH1 | GLI1 |
| G6PD | GPBAR1 |
| GAA | GSK3B |
| GAPDH | GSTM2 |
| GBA | GSTP1 |
| GRM5 | HDAC6 |
| GSK3B | HMGCR |
| GSR | HMOX1 |
| HDAC6 | HPRT1 |
| HK2 | HPSE |
| HMGCR | HSD11B1 |
| HMOX1 | HSD11B2 |
| HPSE | HSP90AA1 |
| HSD11B1 | HTR2A |
| HSD11B2 | ICAM1 |
| HSPA5 | IDH1 |
| HSPA8 | IDO1 |
| HTR1A | IGF1R |
| HTR2A | IGFBP3 |
| HTR3A | IKBKB |
| ICAM1 | IKBKG |
| IDH1 | IL2 |
| IDO1 | IL6 |
| IGF1R | IL6ST |
| IGFBP3 | IMPDH1 |
| IKBKB | ITGA4 |
| IL2 | ITGAL |
| IL6 | ITGB1 |
| ITGAL | ITGB2 |
| ITGB1 | JAK1 |
| ITGB2 | JAK2 |
| JAK2 | JAK3 |
| JAK3 | JUN |
| JUN | KAT2B |
| KCNA5 | KCNH2 |
| KCNJ11 | KDR |
| KCNMA1 | KIT |
| KDR | LANCL2 |
| KIT | LCK |
| LGALS3 | LDHA |
| LGALS9 | LGALS3 |
| LIPA | LGALS9 |
| LTA4H | LIPA |
| MAOA | LRRK2 |
| MAOB | LTA4H |
| MAPK1 | LTB4R |
| MAPK14 | MALT1 |
| MAPK8 | MAPK1 |
| MC4R | MAPK14 |
| MDM2 | MAPK8 |
| MGLL | MDM2 |
| MME | MME |
| MMP1 | MMP1 |
| MMP12 | MMP12 |
| MMP13 | MMP13 |
| MMP2 | MMP14 |
| MMP3 | MMP2 |
| MMP7 | MMP3 |
| MMP8 | MMP7 |
| MMP9 | MMP8 |
| MPO | MMP9 |
| NAAA | MPO |
| NFE2L2 | NCSTN |
| NLRP3 | NFE2L2 |
| NOS1 | NLRP3 |
| NOS2 | NOS1 |
| NOS3 | NOS2 |
| NOX4 | NOS3 |
| NR1H3 | NR0B1 |
| NR1I2 | NR1H2 |
| NR3C1 | NR1H4 |
| NR3C2 | NR1I2 |
| NTRK1 | NR1I3 |
| OPRK1 | NR3C1 |
| OPRM1 | NR3C2 |
| P2RX7 | NTRK1 |
| PARP1 | OPRM1 |
| PDE4D | P2RX7 |
| PDE5A | PER2 |
| PIK3CA | PGR |
| PIK3CD | PIK3CA |
| PIK3CG | PIK3CD |
| PIK3R1 | PIK3CG |
| PLA2G1B | PIK3R1 |
| PLA2G2A | PIM1 |
| PLA2G6 | PIM3 |
| PNP | PLA2G1B |
| PPARA | PLA2G2A |
| PPARD | PLA2G6 |
| PPARG | PPARA |
| PRKCA | PPARG |
| PRKCD | PRKCA |
| PRKCH | PRKCB |
| PSEN1 | PRKCD |
| PSEN2 | PRKCQ |
| PTGER2 | PSEN1 |
| PTGES | PSEN2 |
| PTGIR | PTAFR |
| PTGS1 | PTGER4 |
| PTGS2 | PTGES |
| PTPN1 | PTGS1 |
| PTPN11 | PTGS2 |
| RBP4 | PTPN11 |
| RELA | PTPN2 |
| ROCK1 | PYGM |
| ROCK2 | RAF1 |
| SELE | RBP4 |
| SELL | RELA |
| SELP | ROCK2 |
| SERPINE1 | RORC |
| SGK1 | S1PR1 |
| SHBG | SCN9A |
| SIGMAR1 | SELE |
| SLC2A1 | SELL |
| SLC5A2 | SELP |
| SLC6A11 | SERPINE1 |
| SLC6A13 | SGK1 |
| SLC6A2 | SHBG |
| SLC6A3 | SI |
| SLC6A4 | SLC29A1 |
| SRC | SLC2A1 |
| STAT3 | SLC6A2 |
| STAT5A | SLC6A3 |
| STK26 | SLC6A4 |
| SYK | SRC |
| TBXAS1 | STAT3 |
| TEK | STAT5A |
| TERT | SYK |
| TGFBR1 | TAAR1 |
| TNF | TEK |
| TNNI3 | TERT |
| TNNT2 | TGFBR1 |
| TRPV1 | TLR9 |
| TRPV4 | TNF |
| TTR | TNNI3 |
| TYMP | TRPV1 |
| VDR | TRPV4 |
| VEGFA | TTR |
| XDH | TYK2 |
| XIAP | TYMP |
|  | TYR |
|  | VDR |
|  | VEGFA |
|  | XDH |
|  | XIAP |
|  | ZAP70 |
